# A Versatile Sample Processing Workflow for Metagenomic Pathogen Detection

**DOI:** 10.1038/s41598-018-31496-1

**Published:** 2018-08-30

**Authors:** Claudia Wylezich, Anna Papa, Martin Beer, Dirk Höper

**Affiliations:** 1 Institute of Diagnostic Virology, Friedrich-Loeffler-Institut (FLI), 17493 Greifswald-Insel Riems, Germany; 2 Department of Microbiology, Medical School, Aristotle University of Thessaloniki, 54124 Thessaloniki, Greece

## Abstract

Metagenomics is currently the only generic method for pathogen detection. Starting from RNA allows the assessment of the whole sample community including RNA viruses. Here we present our modular concerted protocol for sample processing for diagnostic metagenomics analysis of human, animal, and food samples. The workflow does not rely on dedicated amplification steps at any stage in the process and, in contrast to published methods, libraries prepared accordingly will yield only minute amounts of unclassifiable reads. We confirmed the performance of the approach using a spectrum of pathogen/matrix-combinations showing it has the potential to become a commonly usable analytical framework.

## Introduction

**Figure 1:**
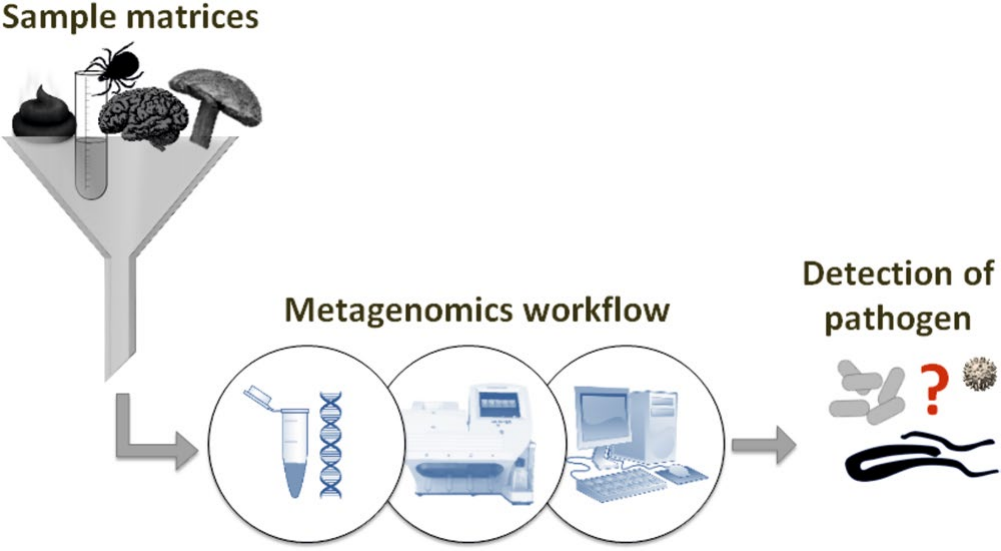
Sketch of the basic idea of a “one serves all” analytical framework^20^. Images used as symbols were obtained from the free websites https://pixabay.com/.

Diagnostic metagenomics with high-throughput sequencing (HTS) techniques continuously gains importance for broad and swift identification of pathogens in human, animal, and food samples^1^. While for known pathogens, highly sensitive and specific diagnostic methods like real-time quantitative PCR (qPCR) are in routine use and deliver reliable results, the identification of unrecognized pathogens, meaning unexpected or newly emerging pathogens or pathogens that are only distantly related with known ones, can be very difficult. In this respect, metagenomics using HTS are much more promising than routine diagnostics. Unrecognized pathogens, especially newly emerging zoonoses, may cause serious infectious diseases, and a delay of medical treatment or development of vaccines might have fatal consequences for the affected patients and animal stocks. Such delays can be caused by performing numerous laborious screening tests until the potential pathogen is found instead of a single comprehensive screening test. Prominent cases of emerging infectious diseases caused by novel or varying viruses for instance are the discovery of the Middle East respiratory syndrome (MERS) coronavirus in 2012^2^, the tremendous Ebola outbreak in 2014^3^, the report on a novel zoonotic bornavirus^4^, or the detection of the new Schmallenberg virus^5^ affecting domestic and wild ruminants. However, infectious diseases with clinical signs like high fever, diarrhoea, or encephalitis − often life-threatening − can be caused by very different infectious agents^6,7^, not only viruses. In such puzzling cases, a generic approach that works likewise successful and efficient for all pathogen groups, as sketched in Fig. 1, is essential. In a number of review articles^1,8–12^, valuable considerations for this approach have been summarized. In addition to the overall workflow outlined in Fig. 1, after metagenomic analysis, it is desirable to confirm the initial sequencing-based suspicion by other methods and in the ideal case by fulfilling the Henle–Loeffler–Koch postulates^13^ as for example done in case of the Schmallenberg virus^5^.

Metagenomics for generic pathogen detection, so-called diagnostic metagenomics, in its pure form is a broad and undirected approach to find gene sequences or sequence fragments of infectious agents within sequence data sets of the whole community of a sample generated by high-throughput sequencing. A crucial point when handling diagnostic sequencing approaches is to explicitly distinguish between i) high-throughput well-standardisable routine diagnostics for expected known pathogens and ii) diagnostic metagenomics for all including unrecognized pathogens. The first approach is easier to design based on the spectrum of known sequences, testing with specific oligonucleotide primers for pathogens of known identity^14,15^, known tropism, and maybe also of a known proportion within a sample. The latter one − used for unrecognized pathogens like in the case of Schmallenberg virus^5^ − should be a generic approach that can ideally be applied to all samples and pathogens. This approach can indeed be developed and tested using samples of known origin and pathogen content. In case of an emergency, however, there might be no information about nature and proportion of the pathogen and only clinical data could be available, and the sample is a closed book. For these cases, metagenomics is optimally applicable as a sophisticated all-in-one solution even in cases where the nature of the pathogen is not known. If sample preparation is designed to be as unspecific as possible to capture all nucleic acids regardless of their source, this approach is applicable simultaneously for viruses, bacteria, and parasites since all three pathogen groups retain their genetic information in form of nucleic acids. Depending on the sample type, the anticipated pathogen, and the research question, one could extract either DNA or RNA. DNA is suitable for most purposes. However, when metagenomics is used for the detection of unrecognized pathogens, it is recommendable to use RNA as initial template to avoid a priori exclusion of RNA viruses. Targeting RNA will not only capture all cellular organisms but also many relevant RNA viruses, e.g., *Coronaviridae* (SARS and MERS coronavirus), *Filoviridae* (Ebola virus), *Flaviviridae* (Zika virus, hepatitis C virus, tick-borne encephalitis virus), *Orthomyxoviridae* (Influenza A virus), or *Paramyxoviridae* (measles virus).

However, for various matrices and matrix-pathogen combinations, established and validated protocols are missing. Previous diagnostic metagenomics studies dealt with selected sample types (e.g., stool^16,17^; intraocular fluids^18^) or were focused to specific pathogen groups (viruses, bacteria, or parasites). Therefore, the improvement and harmonization of pathogen-independent metagenomics to be used in human and animal health and food safety^19,20^ is necessary. To apply our metagenomics workflow that was originally developed for the detection of viruses^4,5^ to other pathogen groups, namely bacteria and parasites, we tested, refined, and verified the protocols. For that purpose, as much as feasible, different sample types were tested using virus-, bacteria-, or parasite-containing as well as uninfected control samples. We also included conventional food samples − untreated and highly processed − since they were seldom handled and evaluated for metagenomics before^9^. As a result of this effort, we present here in detail a well-harmonized sample processing workflow for diagnostic metagenomics without dedicated amplification steps enabling the detection of diverse pathogens in a broad range of different matrices, applicable with both Illumina or Ion Torrent platforms.

## Results

### Important characteristics of the procedure

**Figure 2:**
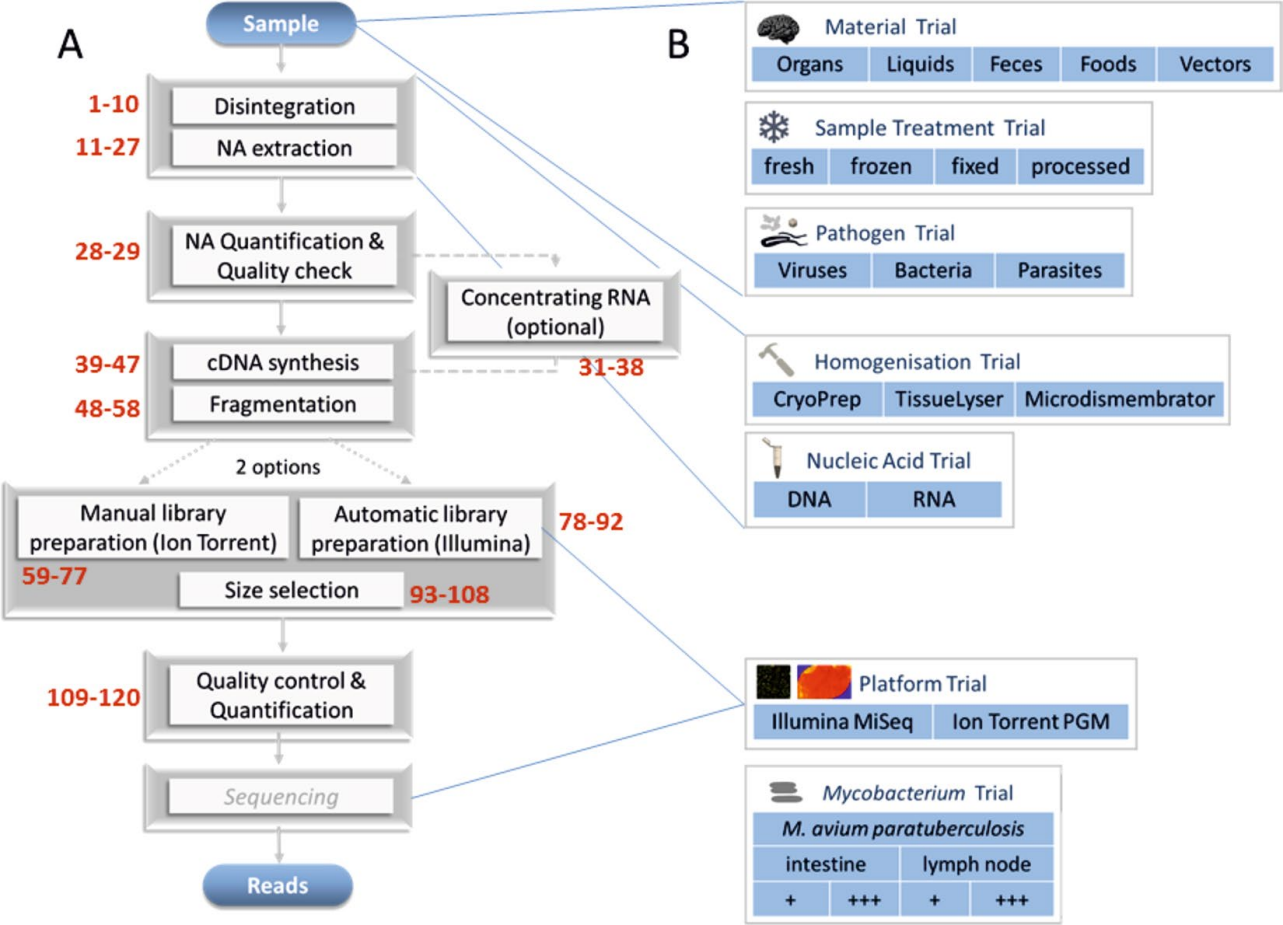
Overview of the workflow and trials for its improvement. (**A**) Overview of the main steps of sample processing. The red numbers refer to the steps given in the procedure (Supplementary File 1). (**B**) Summary of different trials conducted to improve the workflow. NA, nucleic acid. Images used as symbols were obtained from the free websites https://pixabay.com/.

The sample processing workflow as depicted in Fig. 2 and described in detail in Supplementary File 1, is applicable with RNA or DNA as input and has been proven with respect to diagnostic metagenomics in veterinary medicine^4,5,21–27^. Here, the workflow was further tested for different sample types and pathogens as described below.

The workflow starts with a sample disintegration followed by RNA extraction. With only a few exceptions, which are discussed later, the provided protocol is suitable for the extraction of RNA from a broad range of sample types. The protocol proceeds from purified RNA to the final sequencing library with only a single intermediate purification step. This ensures maximum preservation of the information content of the sample.

Routinely, 500 ng (100–1,000 ng) purified total RNA are used for the synthesis of double stranded cDNA in a one-tube reaction, but the protocol is also suitable for extremely low input of RNA, even if the amount cannot be determined. Preferably, RNA solutions with concentrations lower than 10 ng/µl should be concentrated (option in Fig. 2.; Supplementary File 1, Procedure, optional steps 31–38 and Troubleshooting). After cDNA synthesis, the DNA is fragmented without prior purification to avoid loss of material.

Depending on the selected sequencing platform, we provide two possibilities for library preparation (Fig. 2A), one detailed manual procedure for sequencing with Ion Torrent (Supplementary File 1, Procedure, steps 59–77) and one automated procedure for sequencing with Illumina MiSeq (Supplementary File 1, Procedure, steps 78–92). For optimal sequencing results, the library fragment size should be within the specified range of the used sequencing platform and protocols. For both presented sequencing platforms, we apply a target peak size of 550 bp with a size range of 300–1,000 bp. This is achieved with a single two-step size selection procedure using solid-phase paramagnetic bead technology. Because the size of the bound DNA depends on the buffer concentration, calibration of the paramagnetic beads (Supplementary File 1, Reagent setup) is a prerequisite for a reproducible size selection.

### Sample disintegration to extract high quality nucleic acids

**Figure 3:**
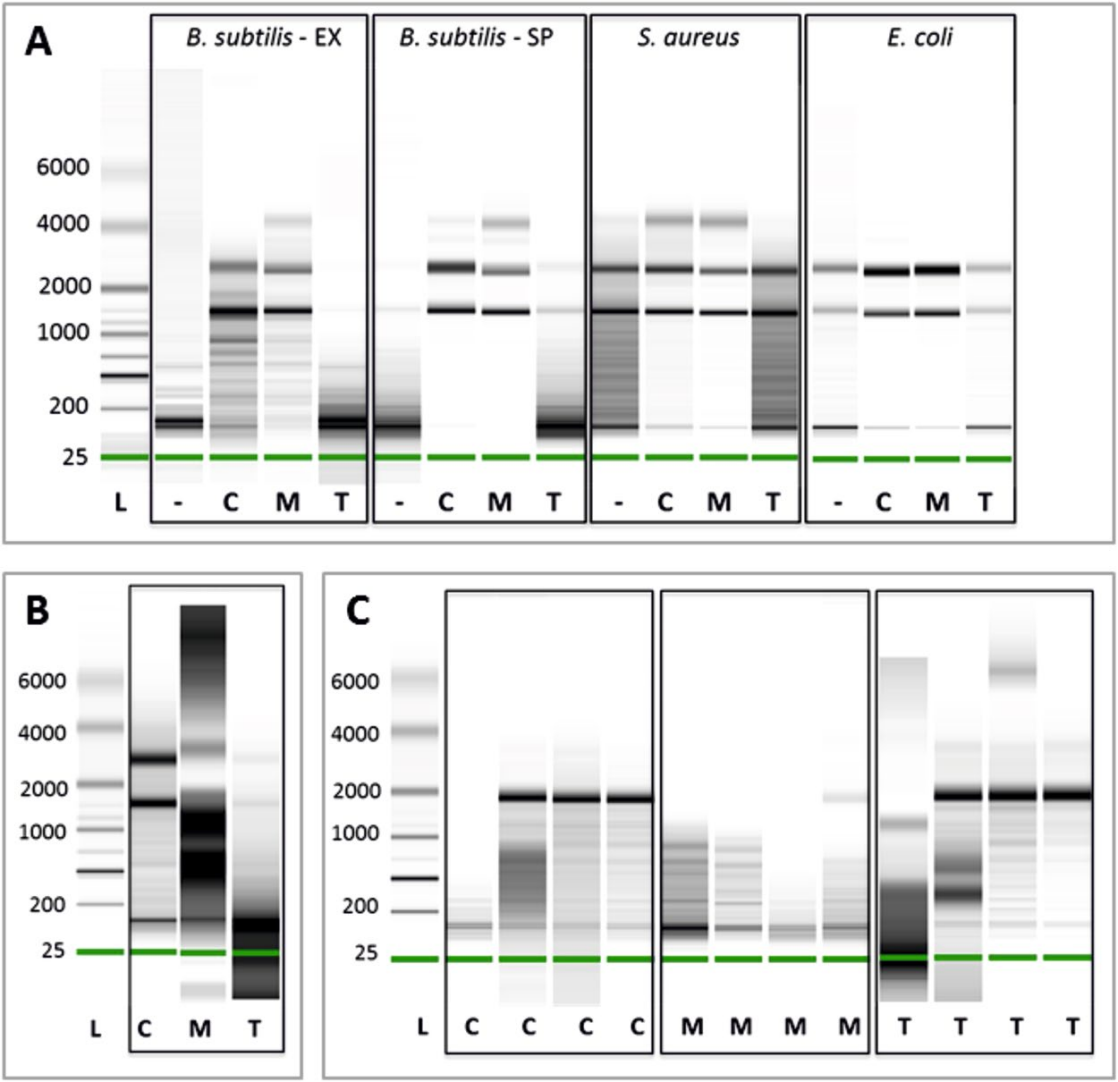
Comparison of different disintegration methods. Shown is the RNA quality analysed using RNA 6000 Pico Chip (Bioanalyzer, Agilent). (**A**) Bacterial suspensions of exponentially growing *Bacillus subtilis* (*B*. *subtilis* – EX) or endospores (*B*. *subtilis* – SP), exponentially growing *Staphylococcus aureus*, or *Escherichia coli*; (**B**) samples of swine faeces; (**C**) pools of midges. Legend: L, ladder; (−), without disintegration step; C, cryoPREP; M, Micro-Dismembrator; T, TissueLyser. The labelling for the ladder (fragment lengths, nucleotides) is given on the left side of each image.

Since the availability of the nucleic acids for library preparation is *the* determinant for the prospect of success of the effort, we compared three different sample disintegration techniques for their suitability to ensure the release of the nucleic acids from the sample material. The applied methods were two bead-beating techniques, one usually conducted in lysis buffer at room temperature, here represented by the TissueLyser, and one conducted with deep-frozen samples, here represented by the Micro-Dismembrator. The third applied technique was cryofracturing using the cryoPREP device. These techniques were tested using a number of different sample matrices and hard-to-break target species. Figure 3A shows the RNA quality achieved with the three techniques in disintegrating suspensions of exponentially growing bacterial cells or hard-shelled Gram-positive endospores (Fig. 3A, see also Supplementary File 1, Fig. A1). Clear bands of small and large subunits of ribosomal RNA, suggestive of high quality nucleic acid, were observed using the cryoPREP impactor or the Micro-Dismembrator grinding mill. In addition, we found a statistically significant (Fisher’s Exact Test, p ≤ 2.2E-16) increase in the proportions of mycobacterial reads in datasets derived from one tap water sample processed with cryoPREP compared to the dataset for the same sample without cryoPREP treatment (compare graphs for library IDs 2093 and 2094 in Supplementary File 2). Likewise, comparing the same datasets, we found statistically significant increases of obligate intracellular *Coxiella* species (3-fold, p ≤ 2.2E-16), of *Parachlamydia*-related species of amoebae (2-fold, p ≤ 2.39E-12), of *Legionella* species (7-fold, p ≤ 2.2E-16), and of Gram-positive Bacillaceae (5-fold, p ≤ 2.2E-16). Like for pure bacterial suspensions shown above, in case of pig faeces, the TissueLyser-disintegrated sample also showed the strongest degradation of RNA i.e. very short RNA fragments (Fig. 3B). In contrast, when pools of midges (insect vectors of orthobunyaviruses like Schmallenberg virus or orbiviruses like bluetongue virus) were disintegrated, cryoPREP and TissueLyser resulted in high quality RNA but not the Micro-Dismembrator (Fig. 3C). Moreover, *Mycobacterium*-containing tissues (lymph nodes and intestine) were used to assess the effectiveness of disintegration using the cryoPREP in comparison with the TissueLyser. The Cq values obtained with DNA extracted after cryoPREP disintegration were for a number of samples substantially lower than those after TissueLyser treatment (Table 1). In summary, generally the best results were achieved for all tested matrices and pathogens with deep-frozen samples using either the Micro-Dismembrator or the cryoPREP device.

**Table 1:** Tested *Mycobacterium*-containing tissues, the grade of infection as investigated by histology and cultivation (given for high-infected (+++) and low-infected (+) samples) and corresponding Cq values. MAP, *Mycobacterium avium paratuberculosis*; Cq, quantification cycle; C, cryoPREP; T, TissueLyser.

Animal	Goat tissue	MAP infection	Cq value for C	Cq value for T
13	Lymph node	+++	**26**.**7**	**40**.**7**
14	Lymph node	+++	**27**.**9**	**30**.**3**
15	Lymph node	+++	**23**.**1**	**32**.**7**
8	Ileum (Peyer’s patches)	+++	**36**.**8**	no Cq
21	Lymph node	+	**31**.**1**	**31**.**8**
23	Lymph node	+	no Cq	no Cq
3	Ileum (Peyer’s patches)	+	no Cq	no Cq

### Workflow verification with samples containing known verified pathogens

For the verification of the workflow, various routine diagnostic samples with pre-diagnosed pathogens were analysed. These samples comprised liquids, tissues, faeces, and foods. Table 2 summarizes results from 15 previously published and 12 new samples. We observed a substantial variation regarding the portion of reads representing the respective expected pathogen. Clearly, the observed variation is mainly caused by the strong background (see Supplementary File 2 for the 100 most abundant families found in each data set) naturally comprised in organ material and other samples (e.g., faeces or different foods) that reduces the pathogen signal in the dataset. Despite of this background, using the provided metagenomics sample processing workflow and a subsequent RIEMS^28^ analysis, it was in all but two cases possible to detect the expected pathogens even if the pathogen load was rather low like in case of chest cavity fluid from a VSBV-1-infected squirrel or for the MAP infected lymph node (Table 2). In the former case, the Cq for VSBV-1 was around 30^4^ and 2 reads representing VSBV-1 were detected. In the latter, the Cq was around 27 after cryoPREP disintegration or nearly 41 after TissueLyser treatment and 0.001% of the reads represented MAP. The workflow works well not only for viral and bacterial pathogens but was also suitable for parasite detection (stool and wild boar in Table 2), albeit the proportion of parasite reads was very low. In case of ethanol-fixed stool samples, *Blastocystis* (0.04%) and *Giardia* (0.0009%) could be detected in a library generated from RNA template yielding little more than 230,000 reads. For the mycobacteria-containing samples (compare Table 1), we detected *Mycobacterium* reads only in the higher laded lymph node sample (Cq 26.7; 23 *Mycobacterium* reads in a total of 2.36E + 6 reads) in contrast to the intestine sample (Cq 36.8; 0 Mycobacterium reads in a total of 1.68E + 6 reads). For two samples presented in Table 2 (chicken liver with Sendai virus, library IDs 1949, 1950, 1951 and wild boar muscle with liver fluke, library IDs 2019 and 2043), technical replicates were processed and sequenced using the present workflow. In both cases, the results are congruent with regard to both the portion of pathogen and unclassified reads. Noteworthy, in all analysed samples, the proportion of unclassifiable reads was very low (Table 2).

**Table 2:** Overview of pre-diagnosed samples containing known verified pathogens.

Category	Sample	Host Species	Known Verified Pathogen	Pathogen Genome	Library ID	DNA/RNA	Total Number Reads	% Pathogen Reads	% Unclassified Reads	Platform	Accession numbers and reference
Liquid	Cell-culture supernatant	Mouse	Rabies virus	ssRNA	1343	RNA	60,275	17.0	0.64	PGM	PRJEB21530^26^
	Cell-culture supernatant	Mouse	Rabies virus	ssRNA	1233	RNA	145,158	84.9	0.62	PGM	PRJEB21530^26^
	Cell-culture supernatant	Sheep	Betacoronavirus	ssRNA	2172	RNA	652,693	57.7	0.39	MiSeq	PRJEB27711, https://www.european-virus-archive.com/virus/betacoronavirus-1-bovines-coronavirus
	Cell-culture supernatant	Green monkey	Rotavirus A	dsRNA	2173	RNA	360,375	24.4	0.42	MiSeq	PRJEB27711, https://www.european-virus-archive.com/virus/rotavirus-rr-1877-bovin
	Cell-culture supernatant	Green monkey	Arrabida virus	ssRNA	921	RNA	911,414	37.7	0.046	MiSeq	KP863799-801^53^
Faeces	Faeces	Pig	PEDV	ssRNA	721	RNA	2,224,040	0.77	1.02	MiSeq	PRJEB19039^24^
	Faeces	Pig	PEDV	ssRNA	799	RNA	2,670,508	10.8	2.68	MiSeq	PRJEB19039^24^
	Faeces	Pig	PEDV	ssRNA	1012	RNA	1,831,855	5.4	1.84	MiSeq	PRJEB19039^24^
	Faeces	Pig	PEDV	ssRNA	1060	RNA	2,020,926	0.28	0.79	MiSeq	PRJEB19039^24^
	Faeces	Pig	PEDV	ssRNA	1420	RNA	1,282,824	19.5	0.38	MiSeq	PRJEB19039^24^
	Stool (ethanol-fixed)	Human	Giardia	DNA	2178	RNA	232,189	0.0009	0.33	PGM	This study
	Stool (ethanol-fixed)	Human	Blastocystis	DNA	2178	RNA	232,189	0.04	0.33	PGM	This study
Tissue	Brain	Dog	Rabies virus	ssRNA	417	RNA	1,257,233	0.52	0.39	MiSeq	LM645022^27^
	Brain	Red fox	Rabies virus	ssRNA	1188	RNA	2,551,046	0.0014	0.98	PGM	PRJEB27711
	Brain	Arctic fox	Rabies virus	ssRNA	985	RNA	329,625	2.8	1.01	MiSeq	LT598540^27^
	Brain	Cat	Rabies virus	ssRNA	325	RNA	1,117,539	0.087	0.068	MiSeq	LM645046^27^
	Brain	Sheep	Rabies virus	ssRNA	300	RNA	2,457,633	0.16	0.059	MiSeq	LM645044^27^
	Brain	Arctic fox	Rabies virus	ssRNA	455	RNA	1,507,356	0.22	1.66	MiSeq	LM645019^27^
	Liver	Chicken	Sendai virus	ssRNA	2019#	RNA	1,249,386	0.017	0.53	PGM	PRJEB27711
	Liver	Chicken	Sendai virus	ssRNA	2043#	RNA	2,521,313	0.017	0.25	PGM	PRJEB27711
	Lymph node	Goat	MAP	DNA	2099	DNA	2,356,712	0.001	0.17	PGM	PRJEB27711
	Intestine	Goat	MAP	DNA	2100	DNA	1,677,552	0.0	0.17	PGM	PRJEB27711
Food	Frozen berries	Strawberries	Norovirus	ssRNA	1962	RNA	933,881	0.0	0.33	PGM	This study
	Muscle	Wild boar	Trichina	DNA	1806	RNA	1,627,079	0.17	0.38	PGM	PRJEB27711
	Muscle	Wild boar	Liver fluke	DNA	1949$	RNA	568,673	0.0065	0.68	PGM	PRJEB27711
	Muscle	Wild boar	Liver fluke	DNA	1950$	RNA	491,774	0.0037	0.20	PGM	PRJEB27711
	Muscle	Wild boar	Liver fluke	DNA	1951$	RNA	555,425	0.0074	0.25	PGM	PRJEB27711

A graphical representation of read counts for the 100 most abundant families for each of the samples is provided in Supplementary File 2. Some datasets are not publicly available due to the EU General Data Protection Regulation and the Nagoya Protocol but are available from the corresponding authors on reasonable request.

^#^ and ^$^ technical replicates.

Abbreviations: MAP, *Mycobacterium avium paratuberculosis*; PEDV, porcine epidemic diarrhoea virus; PGM, Ion Torrent Personal Genome Machine; MiSeq, Illumina MiSeq.

### Determination of the reagent specific background

**Figure 4:**
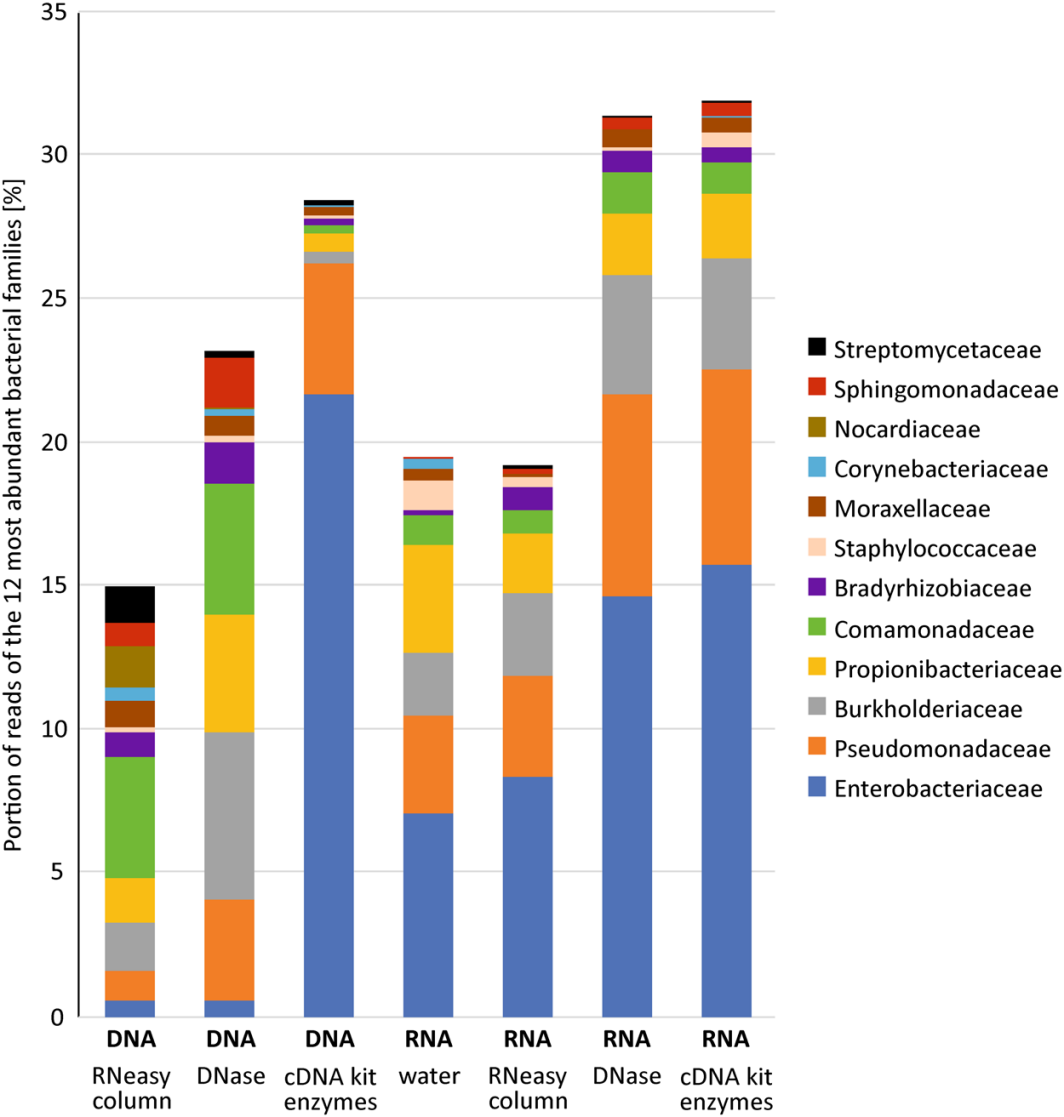
Proportion of bacterial reads found in datasets generated from selected consumables. The samples were processed with the present workflow starting from RNA or from DNA. The read abundance of bacterial reads (top twelve) is given as the percentage of the total dataset.

In order to determine the inherent background of the workflow originating from the used consumables, we extracted both DNA and RNA from selected consumables and prepared and sequenced libraries. With a single exception (library generated from DNA extracted from pooled enzymes of the cDNA synthesis kit, 2.2E +6 reads), sequencing of the libraries generated from the selected consumables resulted in only a few reads (428–4,777 reads) by sequencing the complete extracted material. In the RIEMS^28^ analysed data sets, viral, prokaryotic, and eukaryotic reads were detected (compare Supplementary File 2) with the most frequently detected viral sequences belonging to the Retroviridae. All RNA-derived bacterial profiles were rather similar (Fig. 4). Bacterial groups with the highest read abundances were Enterobacteriaceae (7–21%) and Pseudomonadaceae (3–7%), followed by Burkholderiaceae (2–6%), Propionibacteriaceae (2–4%), Comamonadaceae (1–4%), Bradyrhizobiaceae (about 1%), and Staphylococcaceae (Fig. 4). In contrast, the profiles obtained for the DNA datasets differed substantially between the different reagents. Moreover, the bacterial profiles determined for the DNA and RNA derived data for both the DNase and the RNeasy column were clearly distinguishable. While in the RNA datasets sequences related to the Enterobacteriaceae and the Pseudomonadaceae clearly dominated, in both mentioned DNA samples, the highest proportion was found to be related to the Comamonadaceae (about 4%). Contrarily to the situation in DNase and RNeasy column, the DNA and RNA based bacterial profiles obtained for the pooled enzymes from the cDNA synthesis kit were similar, also resembling the RNA derived profiles obtained for the DNase and the RNeasy column, especially with regard to Enterobacteriaceae and Pseudomonadaceae. All datasets contained eukaryotic reads, mostly mammalian sequences indicating contamination probably from the production process and/or laboratory handling.

### Workflow assessment with various matrices with initially unknown pathogen content

Table 3 lists results obtained from various sample matrices with unrecognized pathogen content from 13 published and 15 new samples. A major group of samples are typical diagnostic materials like different tissues, faeces, and liquids. In a number of the presented examples, novel pathogens were detected using the presented workflow and subsequently confirmed by other methods. In the other cases, sequences putatively representing pathogens were detected (see Table 3); however, these were not confirmed yet.

**Table 3:** Overview of analysed samples of initially unknown pathogen content.

Category	Sample	Host Species	Library ID	DNA/ RNA	Total Number Reads	% Archaeal Reads	% Bacterial Reads	% Eukaryotic Reads	% Viral Reads	% Unclassified Reads	Confirmed Pathogen	Platform	Accession numbers and reference
Liquid	Cell-culture supernatant	Penguin	1003	DNA	982,590	0.014	0.041	72.9	0.61	25.3	Alphaherpesvirus	PGM	LT608136^21^
	Cell-culture supernatant	Penguin	1004	DNA	1,413,128	0.041	0.046	74.1	0.74	23.8	Alphaherpesvirus	PGM	LT608135^21^
	Serum	Cattle	126/11	RNA	26,749	0.022	26.7	69.8	0.42	2.7	Schmallenberg virus	GS FLX	HE649912-14^5^
	Serum	Cattle	127/11	RNA	15,738	0.076	30.3	65.0	0.43	3.6	ND	GS FLX	HE649912-14^5^
	Serum	Cattle	128/11	DNA	75,124	0.0013	0.091	98.0	0.004	1.7	ND	GS FLX	HE649912-14^5^
	Serum	Cattle	129/11	DNA	83,988	0.0024	0.014	98.5	ND	1.3	ND	GS FLX	HE649912-14^5^
	Chest-cavity fluid	Squirrel	651	RNA + DNA	37,816	ND	0.26	78.0	0.67	20.8	VSBV-1	MiSeq	PRJEB27711^4^
	Oropharyngeal swab	Squirrel	648	RNA + DNA	1,000,000#	0.0069	0.70	76.3	0.081	22.9	VSBV-1	MiSeq	PRJEB27711^4^
	Tap water^$^	NA	2091	RNA	50,602	0.012	51.6	44.1	0.6	3.1	ND	PGM	This study
	Tap water	NA	2092	RNA	73,681	0.012	71.5	26.0	0.77	1.7	ND	PGM	This study
	Tap water^$^	NA	2093	DNA	2,277,162	0.12	54.9	2.2	0.12	38.5	ND	PGM	This study
	Tap water	NA	2094	DNA	1,864,200	0.13	58.4	2.8	0.054	35.0	ND	PGM	This study
	Rumen	Cattle	1005	DNA	917,596	1.1	33.5	6.3	0.19	56.1	ND	PGM	PRJEB27711
Faeces	Bird faeces	NA	2177	RNA	2,264,941	0.0015	51.0	36.8	0.44	10.2	ND	PGM	This study
Tissue	Organ-pool (kidney, liver lung)	Squirrel	652	RNA + DNA	349,819	0.0034	0.15	71.1	0.14	28.7	VSBV-1	MiSeq	PRJEB27711^4^
	Organ-pool (Heart, brain)	Squirrel	653	RNA + DNA	367,299	0.0035	0.31	76.8	0.27	22.6	VSBV-1	MiSeq	PRJEB27711^4^
	Brain	Cattle	852	RNA	388,206	0.0093	49.4	44.2	0.47	5.8	Bovine astrovirus	MiSeq	LN879482^23^
	Organ-pool (brain, spinal cord, spleen)	Sheep	1454	RNA	971,433	0.0001	0.005	95.8	0.13	0.66	Ovine astrovirus	PGM	LT706531^22^
	Organ-pool (brain, spinal cord, spleen)	Sheep	1455	RNA	993,038	ND	0.004	95.1	0.91	0.67	Ovine astrovirus	PGM	LT706530^22^
Vector	Pooled ticks	NA	1163	RNA	192,549	0.001	8.4	89.4	0.009	0.59	ND	PGM	This study
	Pooled ticks	NA	1164	RNA	2,210,546	0.0005	6.4	91.0	0.0005	0.58	ND	PGM	This study
	Pooled midges	NA	1081	RNA	2,545,182	0.047	7.1	74.5	0.18	18.2	ND	MiSeq	This study
	Pooled midges	NA	1082	RNA	1,429,726	0.013	3.1	91.4	0.023	5.5	ND	MiSeq	This study
Food	Leaf	Rocket	1497	RNA	439,328	ND	0.064	97.6	0.15	0.32	ND	PGM	PRJEB27711
	Fruiting body	Mushroom	1469	RNA	2,826,378	ND	0.42	92.9	0.094	0.32	ND	MiSeq	PRJEB27711
	Pizza with mushrooms	NA	1960	RNA	427,509	0.0002	0.55	94.5	0.054	1.5	ND	PGM	PRJEB27711
	Crude ham	Pig	1496	RNA	582,692	ND	0.16	96.7	0.063	0.73	ND	PGM	PRJEB27711
	Meat loaf	NA	1488	RNA	360,151	0.0006	3.6	93.3	0.11	0.76	ND	PGM	PRJEB27711

A graphical representation of read counts for the 100 most abundant families for each of the samples is provided in Supplementary File 2. Some datasets are not publicly available due to the EU General Data Protection Regulation and the Nagoya Protocol but are available from the corresponding authors on reasonable request.

^#^partial random subset of the original published dataset.

^$^w/o CryoPrep treatment prior to nucleic acid extraction; the tap water libraries are derived from one sample split into four subsamples.

Abbreviations: VSBV-1 Variegated squirrel bornavirus 1; PGM, Ion Torrent Personal Genome Machine; MiSeq, Illumina MiSeq; GS FLX, Genome Sequencer FLX; ND, none detected.

Arthropod vectors represent an individual type of sample matrix that might require a special treatment to ensure successful analysis (compare Fig. 3). Therefore, alternative homogenization and extraction options are provided here for ticks and midges (see options A and B of the Procedure). Different tick species (*Ixodes ricinus*, *Ornithodoros porcinus*, and *Rhipicephalus bursa*) were subjected to the described procedure and in the generated datasets, *Rickettsia* spp. were re-detected (previously detected via PCR^29^). In addition, the known tick-transmitted bacterial human and animal pathogens *Anaplasma* spp., *Francisella* spp. and *Mycobacterium* spp. were found (compare results for library IDs 1163 and 1164 in Supplementary File 2).

In addition to the aforementioned specimens, we also tested a number of highly processed food samples (Table 3). In all cases (meat loaf, pizza, crude ham), the resulting DNA libraries were of high quality, allowing the taxonomic classification of the vast majority (>98.5%) of the obtained reads. The proportion of unclassified reads ranged between 0.7% and 1.5%. As expected, in case of crude ham (see Table 3) we did not detect any sequences potentially representing pathogens within a dataset of roughly 600,000 reads (Supplementary File 2). Of these reads, RIEMS^28^ classified the vast majority of the reads as mammalian sequences and most of the remainder (558 reads) as *Lactobacillus* spp. Roughly 99% of the viral sequences detected in the crude ham were eukaryotic rRNA sequences misclassified as Arenavirus sequences. Further putative viral sequences represented phages.

## Discussion

Pathogen detection via metagenomics comprises the general steps sampling, sample processing, sequencing, and data analysis. Since the sequencing itself is highly standardized by the suppliers, this is not part of the presented protocol. We also do not cover data analyses here, although this ultimately is an important determinant of the sensitivity and especially the specificity of the overall effort. However, various tools for data analyses together with evaluations of their sensitivity and specificity are available; therefore, we do not cover this part. Here, we focus on sample processing since improper sample processing can lead to loss of information before sequencing.

The presented metagenomics workflow was already proven suitable for the detection of new viral pathogens in both animal and human samples (see Table 3). Examples of new animal RNA viruses are Schmallenberg virus^5^ and novel bovine and ovine astroviruses that caused different neurological symptoms including unusual behaviour and encephalitis^22,23^. A new Alphaherpesvirus of penguins, the most likely causative agent of diphtheria-like disease of banded penguins, represents the group of the DNA viruses that were detected and characterized using the presented workflow^21^. The protocol was also successfully applied to human samples and with the variegated squirrel bornavirus 1 (VSBV-1), a novel zoonotic bornavirus was identified^4^. Besides the beforementioned discovery of RNA viruses with single-stranded genomes^4,5,22–24^, also double-strand RNA viruses like bluetongue virus^25^ or a Rotavirus A (strain RR 18/77 (bovin); see https://www.european-virus-archive.com/virus/rotavirus-rr-1877-bovin) have been detected and fully sequenced. In all aforementioned cases, either of three different sequencing platforms (compare Table 3) was used, showing that the presented workflow is platform independent.

In the examples above, novel viruses were identified to be the infectious agents indicating that virus discovery is a key aspect of pathogen detection by metagenomics. Because the versatility is a great benefit of metagenomics, we assessed the suitability of our sample processing procedures with various sample matrices and pathogens representing bacteria and parasites. The results compiled in Tables 2 and 3 and in the supplement (Supplementary File 2) clearly show that the workflow is indeed suitable for their identification. In addition, it is not only possible to identify and characterize a single pathogen, but also coinfecting pathogens contained in a certain sample. In this way, coinfections of viruses and bacteria, as for instance in cases of porcine epidemic diarrhoea virus (PEDV) infected pigs^24^, or different parasites, e.g. a human co-infection with *Blastocystis* and *Giardia* (see Table 2), could be detected. This underlines the additional ability of diagnostic metagenomics to find secondary infections^30^ that could mutually intensify their pathogenic effect. Altogether, the presented examples suggest that the introduced workflow is suitable for a wide variety of sample matrices in combination with various pathogens and for use with different sequencing platforms.

Release of nucleic acids from the sample is an important prerequisite for successful pathogen detection since nucleic acids enclosed within bacterial or parasite cells or host tissues will not be accessible for sequencing. Therefore, three disintegration techniques were compared with regard to handling and performance. The applied methods were two bead-beating techniques (TissueLyser, Micro-Dismembrator) and cryofracturing using the cryoPREP device. Noteworthy, only the TissueLyser is suitable for high-throughput applications; the Micro-Dismembrator bears a relatively high risk of cross-contamination due to the necessity of re-using the grinding vessels and balls. Like the Micro-Dismembrator, the cryoPREP is not suitable for high-throughput but contrarily bears a very low risk of cross-contamination. The cryoPREP as well as the Micro-Dismembrator procedures include a deep-freezing step preventing degradation and shearing of the nucleic acids that seems to be crucial to obtain high quality RNA (compare Fig. 3). According to our experience (Fig. 3, Table 1), the cryoPREP technique has the highest reliability in making nucleic acids accessible in a gentle manner especially in comparison with the often-used bead-beating of unfrozen samples. Even with *Mycobacterium* species known to be highly resistant against many disintegration and nucleic extraction procedures^31^, the proposed procedure was successfully applied (see Table 1 and library ID 2099 in Table 2). Likewise, high-quality RNA could be extracted from Gram-positive bacteria and their endospores (shown in Fig. 3A) and improved RNA release from pathogens enclosed in host cells like Chlamydiae, *Legionella*, and *Coxiella* made them more readily detectable after cryoPREP treatment. The same was found for *Blastocystis* and *Giardia* which could be detected in a RNA-based sequence data set generated from ethanol-fixed stool samples (see library ID 2178 in Table 2). Both parasites could not be detected using a universal metagenomics approach that started with a bead-beating step but without freezing or cooling the samples during this step^16^. The presented examples corroborate (i) the necessity to apply a gentle yet efficient sample disintegration for metagenomics in cases where the nature of the pathogen is not yet known and (ii) the notion that cryoPREP processing apparently makes the nucleic acids of complex samples accessible even if enclosed in solid host tissues like lymph nodes or by robust cell or cyst walls (bacteria, parasites) or in bacterial endospores. This is especially important for metagenomics intended to be applicable for generic pathogen discovery as described here.

Most material can be processed with the present workflow to allow for pathogen detection. Within this study, we used the presented protocols to sequence different sample materials representing original specimens (Tables 2 and 3). For example, in the crude ham sample, which contained mostly mammalian reads, most of the non-mammalian reads were classified as *Lactobacillus* spp., which are frequently used as food additives^32^ highlighting the potential to find meaningful bacterial reads in RNA-based data sets. Interestingly, identical virus sequences belonging to Narnaviridae were detected in both the pizza with mushroom and the mushroom sample. Even though these detected viruses are no severe pathogens for humans and animals, their detection still shows the fidelity of the present sample processing workflow.

In cases of unrecognized pathogens with hence unknown tissue tropism, the choice of proper sample materials often is a challenge since detectable pathogen loads may be restricted to a special organ or fluid, depending on the pathogen’s tissue tropism. Sequencing of DNA and RNA in parallel^5^ might be recommendable in emergency cases when the pathogen nature is not clear. Therefore, it can be advantageous to use pooled samples (different organs/sample types) to enable a successful detection^8^, but sample pooling might also have adverse effects as for instance for microarray experiments (reviewed in ref.^33^). Moreover, sample pooling can help increase the throughput and enable an efficient screening of samples with a higher pathogen load. However, for samples with low pathogen loads, care must be taken since pooling can have a significant impact, namely by loss of pathogen information due to dilution, further enhancing the unfavourable pathogen-host ratio.

When searching for unrecognized infectious agents via untargeted metagenomics, no pathogen groups should be excluded a priori from sequencing by the applied sample preparation. In the presented workflow, in order to avoid significant distortions of the original composition of the sample’s microbial community and hence loss of information, we deliberately excluded steps intended to introduce bias in a certain direction. More precisely, the workflow completely excludes manipulations like enrichment of target or depletion of supposed non-target molecules for instance by filtration or centrifugation, PCR amplifications and any other manipulation. As shown, manipulation can work well but can have different effects on different viruses^34,35^, and pathogens other than those targeted may be completely lost^34^. In another example, a comparison of metagenomics with a respiratory virus PCR panel resulted in a 53% higher success rate for the metagenomics approach^36^. By targeting certain taxa or genotypes, PCR self-evidently influences quality and quantity of the sequencing outcome and hence reduces the fidelity to reflect the complexity of the original sample in the final data set^37,38^. Especially random PCR can bias the sequencing^39^ and can lead to the generation of a high proportion of unclassifiable sequences^40^. This was also shown for serum samples and faeces suspensions resulting in 10% and 80% unclassifiable reads, respectively^41^. In contrary, applying the presented workflow for metagenomics of swine faeces^24^, on average only 1.8% (median 1.1% for 41 datasets) of the generated reads were unclassifiable (compare Library IDs 721, 799, 1012, 1060, 1420 in Table 2). Likewise, in other examples shown in Tables 2 and 3 only a minor fraction of the obtained reads could not be classified, for instance for pizza, meat loaf, wild boar with liver fluke, and for the MAP datasets. The samples in Table 3 for which high proportions of reads remained unclassified all represent underexplored taxa like *Sciurus variegatoides* or environments like rumen, for which the published analyses were mainly 16 S rRNA-based. Therefore, the results clearly demonstrate the high quality of the libraries generated with the present workflow.

### Current limitations

Here, a wide array of matrices was processed successfully, as proven by the presented results. Nevertheless, challenges for sampling and sample processing remain. Materials that change their physical condition upon deep freezing, like e.g. gummy bears that become glass-like and spawn sharp-edged shivers when being cryofractured, ultimately destroying the Covaris TissueTUBE. Also, samples with a low pH that interferes with nucleic acid extraction (compare Table 2, norovirus polluted frozen berries, library ID 1962), appeared to pose a problem without pH adjustment, although failure to detect norovirus might have had other reasons as it was already reported that noroviruses are hard to detect by metagenomic sequencing^42^.

Two main areas generally impose problems for metagenomics pathogen detection; namely, the necessary reference sequences available in public databases (see next paragraph) and the available sample materials. While the former needs a concerted action of the scientific community to improve, the latter is in the hands of the individual labs. Usually, the best-suited raw material for metagenomic analysis is fresh or fresh-frozen and untreated, since nucleic acid integrity is compromised by prolonged storage or fixation. Although pathogen detection may still be possible despite fixation (compare library ID 2178 in Table 2), awareness needs to be raised that untreated aliquots of samples should be stored deep-frozen when intending metagenomic analysis. Highly processed food samples may likewise be difficult since their RNA content and integrity seems to be inherently low, probably due to the processing. Irrespective of processing, some foods impose difficulties as for instance fatty matrices like cheese or milk, or fruits with low pH (see above). If compatible with the respective workflow, countermeasures like defatting or pH adjustment may be introduced. Hard-to-break matrices like feathers, skin, or plant materials with high fibre content (e.g., oat-flakes) may need a dedicated assessment of the suitability of disintegration procedures. Applying our workflow, the detection of yet unconfirmed plant pathogens in the obtained datasets from both untreated and processed foods (compare Table 3) was possible. Regardless of sample type and workflow, problems can arise when faced with low pathogen loads and hence disadvantageous pathogen-host ratio, like in the MAP samples (Table 2, library IDs 2099 and 2100) and the fresh-frozen liver contaminated with liver fluke (Table 2, library IDs 1949-50). In the latter case, the problem was potentially caused by the patchy distribution of this relatively large parasite, resulting in samples with varying pathogen load. The problem of unfavourable pathogen/host ratios might be compensated by enhanced sequencing depth (see^8^) or host depletion/target enrichment. As pointed out already, the latter may lead to loss of information. In case of low total sample input, the DNA and/or RNA potentially contaminating the used consumables can significantly outcompete the target nucleic acids. Sequencing of kit components used in our workflow revealed retroviral sequences from the cDNA synthesis kit and bacteria also previously found in blank controls^43^ (Fig. 4). Moreover, cross-contamination of libraries due to adapter swapping^44^ or carry-over between runs^8,45^ has to be considered.

For comprehensive and reliable metagenomic analysis, reliable reference sequences are required. However, according to Klimke *et al*.^46^, “different annotation procedures, numerous databases, and a diminishing percentage of experimentally determined gene functions have resulted in a spectrum of annotation quality”. Many organisms (hosts, symbionts, and pathogens) have not yet been sequenced and hence no reference sequences are available. Furthermore, the taxonomic identity associated with some sequences in public repositories^47,48^ have been found to be questionable. This appears to be also the case with the Arenavirus reference (Accession KF478765) that leads to frequent false positive detection of Areanviruses (compare Supplementary File 2). This is likely caused by an extension of the viral genome with a ribosomal sequence. These problems need to be solved in the future to further improve the use of metagenomics for pathogen detection.

## Conclusion

Building on the previous experience from virus discovery, we extended the use of the presented workflow for the detection of pathogens other than viruses and tested a broad range of (diagnostic) sample materials. The resulting workflow we present is largely pathogen- and matrix-independent, i.e. it is applicable to at least the tested sample matrices and can potentially be used for all pathogen groups. It is important to mention that a key issue in sample preparation is to make all nucleic acids accessible to sequencing, here tried to achieve by using an efficient but gentle disintegration method. We routinely use this approach as “one serves all” analytical framework^20^ in cases where causative agents of animal diseases and zoonotic infections are unrecognized.

## Methods

### Samples

The performance of the overall workflow or of its individual modules was assessed using a spectrum of different matrices that can be grouped into the five categories liquids, faeces, tissue, vectors, and food. The processed samples were mostly diagnostic specimens representating liquids (serum, cell-culture supernatant, bacterial suspensions, swab samples, tap water, and rumen); faeces (pig, bird and human) as example of a complex inhibitor-rich matrix; organs like brain, heart, liver, lymph nodes, kidney, lung, and intestine to test the efficiency of the protocol on tissue; pools of midges and ticks, respectively, representing arthropod vectors; rocket, mushrooms, ham, meat loaf, pizza, strawberries as examples for different foods. In addition, TissueLyser and cryoPREP disintegrations were compared using goat lymph nodes and intestine from animals infected with *Mycobacterium avium paratuberculosis* (MAP; lymph nodes and intestine) that was available from an approved (Committee on the Ethics of Animal Experiments and the Protection of Animals of the State of Thuringia, Germany; Permit Number: 04-002/12) and previously published animal trial carried out in accordance with relevant guidelines and regulations^49^.

We used samples containing a pre-diagnosed pathogen (see Table 2) and samples with unrecognized pathogen content (see Table 3). The known pathogens comprised in the samples represented the groups eukaryotic parasites, bacterial pathogens, and viruses. In addition, bacterial suspensions of exponentially growing *Bacillus subtilis*, *Staphylococcus aureus*, and *Escherichia coli*, representing Gram-positive and Gram-negative bacteria, respectively, and an endospore suspension of *B*. *subtilis* as example of nucleic acids protected by highly resistant envelopes were processed. For selected samples, a sequencing library was generated according to the Supplementary File 1 (Procedure, steps 48–120) and sequenced following the respective manufacturer’s instructions.

In addition, we sequenced selected consumables used in our workflow to investigate their impact on the final sequencing outcome. The samples are an RNeasy column taken from the RNeasy Kit (Qiagen), the DNase (Qiagen) as used for the workflow, and the enzymes from the cDNA synthesis kit (Roche). The latter are the components “vial 2” (AMV RT), “vial 4” (Protector RNase Inhibitor), “vial 10” (2^nd^ strand enzyme) and “vial 11” (T4 DNA Polymerase). For all samples, we extracted RNA as described and DNA using the QIAamp DNA Mini Kit (Qiagen) and prepared libraries as described in the Supplementary File 1. In addition, as a blank control, an 800-µl water sample (Roth) was processed with the present workflow.

### Sample processing procedure

Detailed easy-to-follow single protocols (modules) for all steps depicted in Fig. 2A including necessary chemicals and important remarks (reagent setup, troubleshooting, anticipated results) are given as Supplementary File 1. In the following, only procedures supplementing the detailed protocol for comparisons are outlined.

### Sample disintegration

We compared different sample disintegration techniques, namely the laboratory grinding mill Micro-Dismembrator (Sartorius, Göttingen, Germany), the TissueLyser (Qiagen, Hilden, Germany), and the cryoPREP impactor (Covaris, Brighton, UK). Sample disintegration using the Micro-Dismembrator was essentially performed as described^27^. Using the TissueLyser, tubes prepared with the sample material, a steel grinding ball and 200–1000 µl AL buffer (Qiagen, Hilden, Germany) were shaken for 150 s with a frequency of 30 Hz as previously described^50,51^. With a Micro-Dismembrator (Sartorius, Göttingen, Germany), the samples were ground frozen in liquid nitrogen for 2 min at 2000 rpm in a 3 ml PTFE shaking flask with a 10 mm stainless steel ball and the frozen homogenate was further processed according to the detailed protocol (Supplementary File 1, from step 9). The cryoPREP protocol is given in detail in Supplementary File 1 (Procedure, steps 1–10). RNA was extracted following the detailed protocol and quality was checked with a Bioanalyzer (Agilent) using a RNA 6000 pico assay according to the manufacturer’s instructions. DNA from *Mycobacteria* containing samples was extracted using the QIAamp DNA Mini Kit (Qiagen, Hilden, Germany) and quantified regarding the content of mycobacterial DNA via real-time PCR (insertions element IS900^52^).

### Bioinformatic analysis of metagenomic datasets

Obtained raw reads were analysed using the software RIEMS^28^ to get an overview of the taxonomic composition of reads.

## Electronic supplementary material

Supplementary files

## Data Availability

Datasets generated and analysed during the current study are available in the European Nucleotide Archive (ENA) under the study accession number PRJEB27711. Some datasets are not publicly available due to the General Data Protection Regulation (regulation (EU) No 2016/679) and the Nagoya Protocol (regulation (EU) No 511/2014) but are available from the corresponding authors on reasonable request.
