# Supplementary material for: A Versatile Sample Processing Workflow for Metagenomic Pathogen Detection
Source: Sci Rep. 2018 Aug 30;8:13108. doi: 10.1038/s41598-018-31496-1 (PMC6117295; doi:10.1038/s41598-018-31496-1)
Supplement: Supplementary file 1 — Supplementary files [file 41598_2018_31496_MOESM1_ESM.pdf]

A Versatile Sample Processing Workflow for Metagenomic Pathogen Detection

Claudia Wylezich[1*], Anna Papa[2], Martin Beer[1], Dirk Höper[1*]

[1]Institute of Diagnostic Virology, Friedrich-Loeffler-Institut (FLI), 17493 Greifswald-Insel Riems, Germany.

[2]Department of Microbiology, Medical School, Aristotle University of Thessaloniki, 54124 Thessaloniki, Greece.

Supplementary file 1: Metagenomics sample processing protocols consisting of reagents, equipment, reagent setup, and the procedure (steps 1-120, with options for RNA extractions from ticks or mosquitoes) with timing specifications. In addition, a troubleshooting table is given for selected steps as well as some examples for anticipated results with Figures A1, A2, and A3.

Protocols for Metagenomics Sample Processing

CAUTION Successful RNA extraction demands an RNase-free environment, therefore only RNase-free tubes, aerosol-free pipette tips, and only DEPC-treated (nuclease-free) water must be used. Gloves have to be worn throughout the procedure and changed regularly in order to avoid RNase contamination. In general, only use reagents that do not exceed the expiration date. When working with chemicals, always wear a suitable lab coat, disposable gloves, and protective goggles. For more information, consult the appropriate material safety data sheets (MSDSs), available from the manufacturer.

Reagents

•Lysis buffer AL (Qiagen; cat. no. 19075)
•PBS (Gibco, cat. no. 18912-014):
•Disinfectant for dissection instruments (e.g., gigasept®, Schülke)
•TRIzol® LS Reagent (Life Technologies; cat. no. 10296-028) **! CAUTION** Trizol contains high amounts of chaotropic salts and phenol. Consult the appropriate material safety data sheets.
•RNAlater RNA Stabilization Reagent (Qiagen, cat. no. 1017980)
•QIAamp® RNeasy Mini Kit (Qiagen, cat. no. 74106)
•RNase-Free DNase Set (Qiagen cat. no. 79254)
•Ethanol (96 – 100%, undenatured)
•Ethanol (75%, undenatured, diluted in DEPC-treated water)
•Chloroform
•RNA 6000 Pico Kit (Agilent, cat. no. 5067-1513)
•High Sensitivity DNA Kit (Agilent, cat. no. 5067-4626)
•Roche cDNA-Synthesis System (cat. no. 11 117 831 001)
•Roche 400 µM Random-Hexamer-Primer (cat. no. 11277081001)
•GeneRead DNA Library L Core Kit (Qiagen, cat. no. 180432)
•Barcode Adapters (e.g., Life Technologies, Ion Xpress Barcode Adapters 1-16, cat. no. 4471250)
•Barcode Adapters (25 µM, e.g., Bioo Scientific NEXTFlex 96 DNA Adapters, cat. no. 514106)
•Cartridges, tubes and tips: provided with SPRIworks System II for Roche GS FLX DNA Sequencer (Beckman Coulter, cat. no A84806)
•Agencourt® RNAClean XP beads (Beckman Coulter, cat. no. A63987)
•Agencourt® AMPure XP beads (Beckman Coulter, cat. no. A63881)
•KAPA Library Quantification Kit Ion Torrent (e.g., Roche, cat. no. 07960212001)
•KAPA Library Quantification Kit Illumina® Platforms (e.g., Roche, cat. no. 07960204001)
•0.2 M EDTA pH 8.0
•AFA-grade water (Covaris, cat. no. 520101) or highly purified water (at least ASTM Type III or ISO grade 3) for the M220 Focused-ultrasonicator
•RNase-free water (DEPC-treated)

Equipment

•Covaris cryoPREP CP02
•TissueTUBE TT1 Extra Thick & Plug (e.g., cat no. 520044) and special handle (PN 500231) (both Covaris)
•Thermo Savant FastPrep Bio 101 Fp120 Cell Disrupter Homogenizer
•NanoDrop 1000 Spectrophotometer connected with a computer running the NanoDrop control software
•Bioanalyzer 2100 (incl. Chip Priming Station, IKA Vortexer Model MS3 with chip adapter, Agilent Technologies)
•SPRI-TE Nucleic Acid Extractor (Beckman Coulter) (optional, needed for automated library preparation for Illumina platforms)
•SPRIworks Method Card for Instrument Library II (REF A85410)
•Liquid nitrogen
•Container suitable for safe transport of liquid nitrogen
•Ice pans suitable for liquid nitrogen (e.g., Magic Touch Ice pans; Thomas Scientific, cat no. 1224J40)
•Laminar flow hood
•Chemical fume hood
•two PCR workstations (one master mix box, one template box)
•Thermal block for 1.5-ml tubes
•Dissection instruments (e.g., forceps, scissors, petri dishes)
•Refrigerated centrifuge (for 1.5 - and 2-ml tubes)
•Microcentrifuge (for 1.5- and 2-ml tubes)
•Centrifuge for microtiter plates (e.g., Hettich, Rotina 380R)
•M220 Focused-ultrasonicator with M220 microTUBE holder (Covaris. cat. no. 500301) and fragmentation tubes (MicroTube AFA Fiber Screw Cap 6 x 16 mm, 130 µl; Covaris, cat. no. 520096)
•Thermal shaker (e.g., Eppendorf, Thermomixer Comfort)
•Vortex mixer (e.g., Scientific Industries, Vortex Genie 2)
•Thermal Cycler
•Real-time cycler (e.g., Biorad, CFX96 Real-Time System)
•Magnetic particle concentrator (MPC; e.g., DynaMag™-2, Life Technologies)
•Pipettors: 2.5, 10, 20, 100, 200, 1,000 µl
•Plastic lab ware (0.2-, 1.5- and 2-ml tubes and RNase-free pipette filter tips)
•1.5-ml low-bind tubes (sterile, Nuclease-free)
•96-well Microtiter plates, white, suitable for the used real-time PCR cycler, with suitable sealing film
•Disposable gloves
•Cotton gloves to handle liquid nitrogen
•Personal protective clothing (protective goggles)
•Soft laboratory wipes for cleaning

Reagent setup

Calibration of AMPure beads (adapted from Refs 1, 2) Let the bead suspension equilibrate to room temperature; resuspend thoroughly via vortexing. Prepare a 900 µl aliquot of vigorously mixed Agencourt AMPure XP beads in a 1.5 µl tube. Using this aliquot, pipet AMPure XP beads into 1.5 ml tubes for the Bead:DNA ratios as given in the Table below (vortex the bead aliquot and change pipet tips between each sample; pipet and dispense slowly without aspirating any air and without having beads on the outside of the tip):

Bead:DNA ratio (by volume)	AMPure XP Beads (µl)
0.40:1	40
0.45:1	45
0.50:1	50
0.55:1	55
0.60:1	60
0.65:1	65
0.70:1	70
0.75:1	75
0.80:1	80
0.85:1	85
0.90:1	90

Prepare sample and control DNA Ladder: **Sample DNA Ladder**: Pipet 48 µl of DNA Ladder to a new tube and dilute with 1152 µl Nuclease-free water; thoroughly mix by vortexing and spin down briefly. **Control DNA Ladder**: Dilute 4 µl DNA Ladder with 6 µl Nuclease-free water; thoroughly mix and spin down briefly. Pipet 100 µl of diluted **sample DNA Ladder** to each bead aliquot from step above; mix by pipetting 15 times and incubate 5 min at room temperature. Place the reaction tube onto the MPC for 5 min to separate the beads from supernatant (until the beads have concentrated on the tube wall). Slowly aspirate the cleared solution from the reaction tube without disturbing the beads and discard the solution; keep the reaction tube in the MPC while aspirating the solution. Dispense 500 µl 70% ethanol to each reaction tube and incubate 30 s at room temperature. Slowly aspirate the ethanol from the reaction tube and discard. Repeat the ethanol step. Air-dry the pellet for 10 min with the cap open (keeping on MPC). Take reaction tube from MPC, elute DNA with 10 µl Tris-HCl by careful pipette mixing and incubate for 2 min. Place the reaction tube onto the MPC, incubate for 2 min (until the beads have concentrated on the tube wall) and transfer the supernatant containing DNA Ladder into the respectively labelled clean reaction tube. Use 1 µl of supernatant of each size-selected **sample DNA Ladder** and 1 µl of **control DNA Ladder** on a single Agilent DNA 7500 chip. When the Bioanalyzer analysis is finished, choose the “All Samples” view from the current chip; in the right-hand global settings tab, open “Integrator” in “Sample Setpoints” and set “Height Threshold [FU]” to 5. Copy the DNA concentrations (in ng/µl) for the 200, 300, 400, 500, and 900 bp peaks from each sample’s peak table to an Excel table and calculate the concentration ratios of the individual peaks and the 900 bp peak that serves for normalization. Make sure that the ratios for the control sample fit those given in the table below, if not repeat the experiment. Select the AMPure XP volume per sample volume that best fits the optimal values as per the Table below.

Peak (bp)	Optimal Ratio (Peak/900 bp)	Control Ratio (Peak/900 bp)
200	< 0.1	0.7
300	0.6	1.1
400	1.0	1.4
500	3.0	3.4

Apply the selected Bead:Sample ratio for all future size selections using the calibrated lot. Aliquot the AMPure XP beads in order to prevent repeated warming and cooling as this will degrade the buffer and render the beads non-functional.

DNaseI working solution (Qiagen, prepare on ice, RNase-Free DNase Set) Add 10 µl DNase I stock solution to 70 µl Buffer RDD; mix by gently inverting the tube and centrifuge briefly (do not vortex as DNase I is sensitive to physical denaturation);

Buffer RPE (Qiagen, before first use) From the QIAamp RNeasy Mini Kit: add the appropriate amount of ethanol (96 – 100%) to the buffer concentrate as indicated on the bottle; stable for 1 year when stored closed at RT; shake before use

PBS Each 5 g Gibco® PBS tablet should be dissolved in 500 ml of distilled water. The pH will be 7.45 and requires no adjustment.

DNA Dilution buffer 10 mM Tris-HCl, pH 8.0 + 0.05% Tween 20 (needed for qPCR)

Procedure

CAUTION During both Illumina and Ion Torrent sequencing sample preparation, a clonal amplification step is performed. The amplified DNA is released into buffer before starting the sequencing run. To avoid carry-over of the amplified library DNA to original sample material or sequencing ready libraries, use a one-way facility setup with separate areas for sample processing/library construction and run preparation and never carry back materials from the sequencing to the library preparation area.

Disintegration of sample material • TIMING 30 min per 2 samples

CAUTION Before handling liquid nitrogen, read the appropriate material safety data sheet and operating instructions! Only use the Covaris cryoPREP impactor after having been instructed by trained personnel.

1| Laminar flow (step 1-4): Prepare approximately 20 mg solid or 200 µl liquid sample, place the sample to the centre of the tissueTUBE (TT1) through the top opening using forceps or tweezers.

2| After the sample is loaded, seal the tissueTUBE by screwing the lid on the top of the tissueTUBE but do not screw tightly.

3| While holding the tissueTUBE with the special handle, snap-freeze the sample by immersing the flexible pouch into liquid nitrogen within an ice pan; avoid dipping the cap or transfer tube.

4| Verify that the pouch is not swelled (a sign of trapped air) and that the sample remains centred in the pouch.

5 | Open the cryoPREP lid and use the special handle to insert the frozen tissueTUBE, close the cover, select IMPACT LEVEL 6, and press green ACTIVATE button.

6| Remove tissueTUBE from the cryoPREP impactor using the special handle.

7| Repeat steps 3-6 once.

8| <u>Laminar flow (steps 8-10):</u> Immediately re-chill the tissueTUBE by immersing it in liquid nitrogen.

9| Unscrew the tissueTUBE and resuspend the sample in 1-2 ml lysis buffer AL preheated at 56°C. Do not introduce air to avoid foaming of the sample.

10| Transfer the sample solution into two fresh 2-ml tubes.

> PAUSE POINT *The disintegrated sample in AL buffer can be stored at 4°C over night. We observed integrity of RNA after the disintegrated sample was stored about 3 months at 4°C.*

Extraction of RNA • TIMING approximately 2 h per 2 samples
This protocol (QIAamp RNeasy Mini Kit) is suitable for the purification of RNA from 250 µl sample volume. For higher volumes, the sample can be divided in several subsamples of 250 µl to be process in parallel (steps 11-17) and to load successively on one column (step 18). Sample material can be liquid samples (serum/plasma, cell culture supernatant, EDTA-blood diluted 1:2 in PBS, supernatant from swabs, liquor) or samples homogenized and lysed (tissue and stool homogenates). Alternatively, for ticks and mosquitoes option A and B, respectively, can also be applied.

> CRITICAL STEP Successful RNA extraction demands an RNase-free environment, therefore only RNase-free tubes, aerosol-free pipette tips, and Nuclease-free water must be used. Gloves have to be worn throughout the procedure and changed regularly in order to avoid RNase contamination.

11| Pre-cool the refrigerated centrifuge at 4°C.

12| <u>Chemical fume hood:</u> Pipette 750 µl Trizol LS Reagent into a 2-ml tube and add 250 µl sample and thoroughly shake manually for 15 s.

13| Briefly centrifuge for 5 s at 8,000 r.p.m. and Incubate 5 min at room temperature.

14| <u>Chemical fume hood:</u> Add 200 µl chloroform.

15| Thoroughly shake manually for 15 s and incubate 10 min at room temperature. Centrifuge 10 min at 13,000 r.p.m. at 4°C.

Note All subsequent centrifugations are performed at room temperature.

16| <u>Chemical fume hood:</u> Carefully transfer the upper aqueous phase without disturbing the DNA-containing interphase into a new sterile 2-ml tube.

17| Add 600 µl ethanol (75%), mix by pulse-vortexing and briefly centrifuge for 5 s at 8,000 r.p.m.

18| Apply 600 µl of the sample from step 17 to the column, and centrifuge at 10,000 r.p.m. for 20 s.

19| Discard the collection tube and place the column into a clean 2-ml collection tube, apply residual sample from step 17 to the column and centrifuge at 10,000 r.p.m. for 20 s.

20| Discard the collection tube and place the column into a clean 2-ml collection tube, add 350 µl buffer RW1 to the column and centrifuge at 10,000 r.p.m. for 20 s.

21| Discard the collection tube and place the column into a clean 2-ml collection tube, apply 80 µl DNase working solution (10 µl DNase plus 70 µl RD buffer) to the center of the membrane without touching the membrane.

22| Incubate for 15 min at room temperature, add 350 µl buffer RW1 to the column and centrifuge at 10,000 r.p.m. for 20 s.

23| Discard the collection tube and place the column into a clean 2-ml collection tube, add 500 µl buffer RPE and centrifuge at 10,000 r.p.m. for 20 s.

24| Discard the collection tube and place the column into a clean 2-ml collection tube, add 500 µl buffer RPE and centrifuge at 13,000 r.p.m. for 1 min.

25| Discard the collection tube and place the column into a clean 2-ml collection tube and centrifuge 2 min at 13,000 r.p.m. (to let the column dry).

26| Discard the collection tube and place the column into a clean 1.5-ml tube, add 50 µl RNase-free water to the center of the membrane, incubate 1 min at room temperature and centrifuge 1 min at 10,000 r.p.m.

CAUTION Apply the water provided with the kit to avoid inefficient RNA elution due to inappropriate pH value.

27| Repeat step 26 twice and elute into the same tube.

> PAUSE POINT *RNA is stable for up to one year when stored at -80°C avoiding freeze-thaw cycles. When the RNA will be used for metagenomics, we recommend to subsequently continue with quantification of RNA and ds cDNA synthesis.*

Alternatively, steps 11-17 can be exchanged by option A (for ticks) or option B (for mosquitoes) depending on sampling material.

(A) RNA extraction of ticks.

(i) Take out of the -80°C freezer one tick at the time and proceed with it fast, avoiding thawing. Cut the tick in half vertically.
(ii) Transfer one half of the tick immediately in a 1.7 ml tube containing RNAlater (10 µl RNAlater per 1 mg of tissue) for stabilization and storage.
(iii) Store initially at 4°C overnight, then transfer and store at -20°C.
(iv) Add 180 µl PBS in a 2-ml micro tube and transfer the other half of the tick and one 3-mm glass bead.
(v) Homogenize the tissue by centrifuging 3-4 times at full speed for 20 s each at a FP120 FastPrep Cell Disrupter. Wait 1 min between the centrifuge steps to avoid overheating of the samples.
(vi) Centrifuge homogenized tissue for 3 min at 14,000 r.p.m.
(vii) Transfer 100 µl of supernatant in 250 µl RLT buffer (and store the rest of supernatant in tube

at -80°C)

(viii) Incubate at RT for 10 min and centrifuge for 3 min at 14,000 r.p.m.

(ix) Transfer the supernatant (about 350 µl) to a new microcentrifuge tube by pipetting and add 350 µl of 70% ethanol to the cleared lysate. Mix immediately by pipetting. Do not centrifuge.

(x) Proceed with step 18 of the RNA extraction protocol above.

(B) RNA extraction of mosquitoes.

Number of mosquitoes	PBS
≤40	400 µl
40-70	500 µl
≥70 - 100	600 µl

Volume of PBS used for homogenization according to the number of mosquitoes.

(i) Add the appropriate volume of PBS to the homogenization tubes according to the Table above.

(ii) Add to the homogenization tube glass beads.

(iii) Rinse the mosquitoes with nuclease-free water. Discard carefully the water.

(iv) Add mosquitoes in the homogenization tube containing the culture medium and one 3-mm glass bead.

(v) Homogenize the tissue by centrifuging 3-4 times at full speed for 20 s each at FastPrep centrifuge. Wait 1 min between the centrifuge steps to avoid overheating of the samples.

(vi) Centrifuge homogenized tissue for 5 min at 14,000 r.p.m. and then transfer 100 µl of homogenized tissue in 250 µl RLT.

(vii) Transfer the remaining 300 µl or 400 µl or 500 µl, depending on the sample volume (see also Table above) of the supernatant to a new microcentrifuge tube for stock storage at -80°C.

(viii) Incubate at RT for 10 min and centrifuge for 3 min at 14,000 r.p.m.

(ix) Transfer the supernatant (about 350 µl) to a new microcentrifuge tube by pipetting and add 350 µl of 70% ethanol to the cleared lysate. Mix immediately by pipetting. Do not centrifuge.

(x) Proceed with step 18 of the RNA extraction protocol above.

Quantification and integrity check of RNA • TIMING approximately 1 h per 2 samples

CAUTION Since the NanoDrop quantification can be unreliable [3], quality-checked RNA can be accurately quantified using the Quant-iT RiboGreen RNA reagent.

28| Quantify the purified RNA on a NanoDrop 1000 Spectrophotometer.

29| Confirm RNA integrity using an Agilent Bioanalyzer RNA assay (with the Eukaryote Total RNA Series II.xsy assay). If necessary, adjust the RNA concentration to fit the requirements of the selected assay. From the generated electropherogram, the software calculates an RNA Integrity Number (RIN) that can help in assessing RNA quality.

cDNA synthesis • TIMING approximately 4 h per 2 samples

30| Approximately 500 ng high quality RNA in a maximum volume of 17 µl is needed for cDNA synthesis. In case the concentration is too low, RNA needs to be concentrated with Agencourt RNAClean XP beads (steps 31-38).

CAUTION To avoid contaminations: Tubes containing purified template RNA/DNA must exclusively be opened and handled in a template PCR workstation. Buffers and reagents for preparing mastermixes must exclusively be opened and handled in a clean mastermix box. Change gloves after handling template RNA before working in the mastermix box. Buffers and reagents once opened in the template box must never be used and opened in the mastermix box.

Concentrating RNA before cDNA synthesis (optional) • TIMING approximately 45 min per 2 samples

31| Template box: (steps 30-38): Pipette 1.8 volumes of well-mixed Agencourt RNAClean XP beads to RNA (within low-bind tube) and mix the DNA-bead-solution thoroughly by pipette mixing (15x). Incubate 10 min at room temperature on a shaker at 500 r.p.m.

32| Place the reaction tube onto the MPC for 5 min to separate the beads from supernatant (until the beads have concentrated on the tube wall).

33| Leaving the tube in the MPC, slowly aspirate the supernatant from the tube without disturbing the beads and discard the solution.

34| Leaving the tube in the MPC, dispense 1 ml 75% ethanol to reaction tube and incubate 30 sec at room temperature and slowly aspirate the ethanol from the reaction tube and discard.

35| Repeat step 34 while the reaction tube keeps on MPC.

36| Air-dry the pellet for 10 min with the cap open (keeping on MPC); do not over-dry!

37| Take reaction tube from MPC, elute DNA with 20 µl Nuclease-free water by careful pipette mixing and incubate for 2 min.

38| Place the reaction tube onto the MPC, incubate for 2 min, and pipette 17 µl of the concentrated DNA solution into a clean reaction tube.

cDNA synthesis • TIMING approximately 4 h per 2 samples

39| Mastermix box: Prepare a mastermix for the first strand synthesis using the Roche cDNA-Synthesis System:

**Components**	**1 x**		**x**	
**Vial 1** (5× RT-buffer AMV)	8.0	µl		µl
**Vial 3** (DTT, 0.1 M)	4.0	µl		µl
**Vial 7** (dNTPs, 10 mM)	4.0	µl		µl
**Vial 4** (Protector RNase Inhibitor, 25 U/µl)	1.0	µl		µl
**Vial 2** (AMV RT, 25 U/µl)	2.0	µl		µl
**Total volume**	**19.0**	**µl**		**µl**

40| Mastermix box: Pipette 4 µl of the 400 µM Random-Hexamer-Primer in a 0.2-ml PCR tube.

41| Template box: Add a maximum of 500 ng template RNA in a total volume of 17 µl (from step 27 or optionally from step 38) to the Random Hexamer-Primers, briefly vortex and centrifuge.

42| Incubate 2 min at 95°C in a thermal cycler with hot-lid on and subsequently cool down the reaction for 2 min on ice. Add 19 µl of the first-strand mastermix.

43| Incubate 10 min at 25°C, 60 min at 42°C with hot-lid on (110°C). After the incubation is finished, keep on ice.

Note Briefly before the incubation is finished, start preparing the mastermix for the second-strand synthesis (see below). Before proceeding to the mastermix box, change gloves.

44| Mastermix box: Prepare mastermix for the second strand synthesis using the Roche cDNA-Synthesis System:

**Components**	**1 x**		**x**	
**Vial 9** (5× 2nd strand synthesis buffer)	30.0	µl		µl
**Vial 12** (Water, PCR Grade)	72.0	µl		µl
**Vial 7** (dNTPs, 10 mM)	1.5	µl		µl
**Vial 10** (2nd strand enzyme)	6.5	µl		µl
**Total volume**	**110.0**	**µl**		**µl**

45| Template box: Add 110 µl second strand synthesis mastermix to the first-strand reaction.

46| Briefly mix and centrifuge. Incubate 2 h at 16°C (hot-lid off) in a thermal cycler.

47| Add 20 µl of vial 11 (T4 DNA Polymerase), carefully mix and centrifuge briefly. Incubate 5 min at 16°C. Stop reaction by adding 17 µl 0.2 M EDTA, pH 8.0.

*> **PAUSE POINT** Store cDNA at -20°C or continue directly with the DNA fragmentation.*

Library preparation: fragmentation • TIMING approximately 30 min per 2 samples

CAUTION Never run a method without AFA-grade water in the acoustic assembly; this could damage the transducer. Do not leave water in the water bath for an extended time. Empty the water bath and wipe it dry after use with a lint-free cloth. Do not employ isopropyl alcohol, ammonia-based or abrasive cleaners on the acoustic assembly. Store the tube holder in a dry place.

48| Pipette 130 µl sample in the sample tube (if the sample volume is less than 130 µl fill up to 130 µl with water). Place the tube in the microTube holder and fix it with the sample weight.

49| Start protocol via the start button, repeat fragmentation once by pressing the repeat button. Use the pre-installed DNA shearing protocol DNA_0500_bp_130_ul_Snap_Cap_microTUBE.

50| Pipette the fragmented sample into a clean 1.5 ml tube. If the sample volume is greater than 130 µl, repeat steps 48-49 using the same microTube.

Purification and concentration of fragmented cDNA synthesis products for samples to be sequenced with the Ion Torrent platform • TIMING approximately 45 min per 2 samples

CAUTION When opening a new lot of AMPure XP beads, perform a bead calibration before first use. Aliquot the AMPure XP beads in order to prevent repeated warming and cooling as this will degrade

the buffer and render the beads non-functional. Make sure the Agencourt AMPure XP beads are at room temperature and are thoroughly mixed. The reagent (magnetic particle solution) should appear homogenous and consistent in colour. Do not freeze.

51| Add 1.8 volumes of well-mixed Agencourt AMPure XP beads to each cDNA sample and mix the DNA-bead-solution thoroughly by pipette mixing (15x) and incubate 10 min at room temperature on a shaker at 550 r.p.m.

52| Place the reaction tube onto the MPC for 5 min to separate the beads from supernatant (until the beads have concentrated on the tube wall).

53| Slowly aspirate the cleared solution from the reaction tube without disturbing the beads and discard the solution; keep the reaction tube in the MPC while aspirating the solution.

54| Leaving the tube in the MPC, dispense 1 ml 75% ethanol to the reaction tube and incubate 30 s at room temperature; slowly aspirate the ethanol from the reaction tube and discard.

55| Repeat step 54 while the reaction tube keeps on MPC.

56| Air-dry the pellet for 10 min with the cap open (keeping on MPC); do not over-dry!

57| Take reaction tube from MPC, elute DNA with 27 µl Nuclease-free water by careful pipette mixing and incubate for 2 min.

58| Place the reaction tube onto the MPC, incubate for 2 min. Keeping the tube on the MPC, pipette 25 µl of the concentrated DNA solution into a clean reaction tube.

Library preparation

There are 2 possibilities for library preparation, one manual preparation to be sequenced with the Ion Torrent PGM (Steps 59-77) and one automated preparation to be sequenced with the Illumina MiSeq (Steps 78-92).

CAUTION To avoid contaminations: Tubes containing fragmented and concentrated DNA must exclusively be opened and handled in a template box. Buffers and reagents for preparing mastermixes must exclusively be opened and handled in a clean mastermix box. **Do not use the same barcode as in the sequencing run before.** Change gloves after handling template DNA before working in the mastermix box.

Library preparation for sequencing with Ion Torrent PGM: end-repair • TIMING approximately 45 min per 2 samples

59| Thaw, mix, and shortly centrifuge both end repair and ligation buffer, adapters, and dNTPs and keep them on ice.

60| Mastermix box: Pipette 2.5 µl of End-Repair Buffer in one 0.2 ml PCR tube per DNA sample. Add 2 µl End-Repair Enzyme Mix and keep the PCR tube on ice

61| Template box: Keep DNA samples on MPC some minutes before pipetting to avoid transfer of residual AMPure beads. Add 20.5 µl DNA sample to buffer and enzyme mix for a final total volume of 25 µl. Briefly vortex and centrifuge. *Optionally, save 2 µl of fragmented DNA for troubleshooting.*

62| Incubate in the thermocycler with following temperature profile:

Temperature	Time
25°C	20 min
70°C	10 min
8°C	∞

63| Briefly before the incubation is finished, start preparing the mastermix for the adapter ligation (see step 64). Before proceeding to the mastermix box, change gloves.

Library preparation for sequencing with Ion Torrent PGM: adapter ligation • TIMING approximately 30 min per 2 samples

64| Mastermix box: Prepare mastermix for the adapter ligation:

Components	1 x		x	
**RNase-free water**	8.0	µl		µl
**Ligation Buffer** (2x)	40.0	µl		µl
**dNTP Mix** (10 mM)	1.0	µl		µl
**Universal adapter P1** (to 0.5 µM final conc.)	1.0	µl		µl
**Ligation and Nick Repair Mix**	4.0	µl		µl
**Total volume**	**54.0**	**µl**		**µl**

65| Briefly vortex and centrifuge the mastermix.

66| Mastermix box: Pipette 54 µl of mastermix in one 0.2 ml PCR tube per DNA sample.

67| Mastermix box: Add 1 µl of the chosen barcode adapter to each mastermix aliquot.

CAUTION To avoid contaminations, **open only one barcode adapter tube at a time and change gloves between pipetting the different adapters to avoid cross-contamination**.

68| Template box: Add 25 µl end-repaired DNA (from part End-repair, above), briefly mix and centrifuge. Incubate in the thermocycler with pre-set temperature profile:

Temperature	Time
25°C	10 min
72°C	5 min
8°C	∞

Purification and concentration of IonTorrent Library • TIMING approximately 45 min per 2 samples

CAUTION The end-repaired and adapter-ligated library has to be purified to remove excess of polyethylene glycol (PEG), salts, and enzymes. Otherwise, the high concentration of PEG in the ligation mix will interfere with proper size selection.

69| Template box: (steps 69-77): Pipette 144 µl (1.8 volumes) of well-mixed Agencourt AMPure XP beads to 80 µl of end-repaired and adapter-ligated DNA library and mix the DNA-bead-solution thoroughly by pipette mixing (15x). Incubate 10 min at room temperature on a shaker at 500 r.p.m.

70| Place the reaction tube onto the MPC for 5 min to separate the beads from supernatant (until the beads have concentrated on the tube wall).

71| Leaving the tube in the MPC, slowly aspirate the supernatant from the tube without disturbing the beads and discard the solution.

72| Leaving the tube in the MPC, dispense 1 ml 75% ethanol to reaction tube and incubate 30 sec at room temperature and slowly aspirate the ethanol from the reaction tube and discard.

73| Repeat step 72 while the reaction tube keeps on MPC.

74| Air-dry the pellet for 10 min with the cap open (keeping on MPC); do not over-dry!

75| Take reaction tube from MPC, elute DNA with 27 µl Nuclease-free water by careful pipette mixing and incubate for 2 min.

76| Place the reaction tube onto the MPC, incubate for 2 min, and pipette 25 µl of the concentrated DNA solution into a clean reaction tube.

77| Adding 25 µl Nuclease-free water, repeat the elution (steps 75-76) to increase the yield of library, pool both elutions. *Optionally, save 2 µl of the library for troubleshooting.*

Automated library preparation with the SPRIworks Fragment Library System II for sequencing with Illumina MiSeq: • TIMING approximately 4 hours per 2 samples (including pre-arrangements and running time).

CAUTION Blunt end adapters are not compatible with SPRIworks Fragment Library System II. Do not change the method card while instrument is powered on. <u>To avoid contaminations,</u> **do not use the same barcode as in the sequencing run before.**

78| Completely thaw, mix, and briefly centrifuge all components.

79| <u>Mastermix box</u>: Prepare 3 labelled screw cap tubes provided with the kit for each library: sample tube, adapter tube, library tube. Take off all caps of the tubes; keep the caps of the library tubes.

80| Pipet 2,5 µl of one of the different barcode adapters to the adapter tube and dilute with 2,5 µl Nuclease-free water.

CAUTION <u>To avoid contaminations</u>, **open only one barcode adapter tube at a time and change gloves between pipetting the different adapters to avoid cross-contamination.**

81| <u>Template box:</u> Transfer 500 ng fragmented DNA in a maximum of 400 µl to the **sample** tube, fill up to 400 µl with Nuclease-free water.

82| <u>SPRI-TE Nucleic Acid Extractor (Steps 82-92)</u>: Set up the instrument: Turn off the instrument - Press the EJECT button, remove any method card if necessary and carefully insert the SPRIworks method card for Fragment Library System II (REF A85410) - Close the instrument door - Turn the power on.

83| Open the door only when the screen displays the TOP MENU.

84| When cartridges have completely thawed, ensure that all solutions including magnetic beads are at the bottom of the wells by gently tapping on the bench top.

85| Remove tip/tube rack and reagent rack (in this order) from the instrument.

86| Insert one cartridge per library into the grooved channel of the reagent rack by grasping the labelled lip and sliding it to the back of the channel.

87| Arrange the tubes and tips provided with the kit as displayed in the Table below:

Position in the tip tube rack	Consumables
**1**	Sheath with **Piercing Tip**
**2**	Sheath with **1 ml Tip**
**3**	Sheath with **200 µl Tip**
**4**	Uncapped 2 ml **Adapter Tube** with Adapters
**5**	Uncapped 2 ml **Library Tube**

88| Load reagent rack and tip/tube rack (in this order) into the instrument; the back edge of the tip/tube rack overlaps the reagent rack when loaded correctly.

89| Load the uncapped sample tube containing the fragmented DNA into the circular hole in position 13 of the reagent rack.

90| Start the run: Close the instrument door - Select the green START button - Specify 1 = no size selection and select ENTER to confirm - After a heat block test, the run begins automatically.

91| The run is finished when the instrument displays "Run Complete" - Open the door - Retrieve and cap the library tubes - Discard used sheaths, tips and cartridges.

92| To clean the instrument, select UV-Clean from the TOP MENU.

Size selection of library • TIMING approximately 45 min per 2 samples

93| <u>Template box:</u> (steps 93-108): Resuspend an aliquot of the magnetic particle solution via vortexing. For the upper size selection, dilute Agencourt AMPure XP beads per the Table:

	1 x		x	
**AMPure XP beads**	104.0	µl		µl
**Water** (PCR grade)	80.0	µl		µl
**Sum**	**184.0**	**µl**		**µl**

94| Fill the generated library (from step 77 or 91 depending on sequencing platform) up to 100 µl with Nuclease-free water.

95| Add 160 µl **diluted** Agencourt AMPure XP beads per DNA library to remove large fragments and mix the DNA-bead-solution thoroughly by pipette mixing (15x). Incubate 5 min at room temperature on a shaker at 550 r.p.m.

96| Place the reaction tube onto the MPC for 5 min to separate the beads from supernatant (until the beads have concentrated on the tube wall).

97| Slowly aspirate the cleared solution from the reaction tube without disturbing the beads and pipette the solution into a clean 1.5 ml tube in two steps á 125 µl (exact final volume 250 µl).

98| Add 30 µl **undiluted** Agencourt AMPure XP beads per 250 µl supernatant from step 97 to remove small fragments and mix the DNA-bead-solution thoroughly by pipette mixing (15x). Incubate 5 min at room temperature on a shaker at 550 r.p.m.

99| Place the reaction tube onto the MPC for 5 min to separate the beads from supernatant (until the beads have concentrated on the tube wall).

100| Leaving the tube in the MPC, slowly aspirate the supernatant in two steps á 138 µl from the tube without disturbing the beads and discard the solution.

101| Leaving the tube in the MPC, dispense 200 µl 75% ethanol to reaction tube and incubate 30 sec at room temperature.

102| Slowly aspirate the ethanol from the reaction tube and discard.

103| Repeat step 101-102 while the reaction tube keeps on MPC.

104| Air-dry the pellet for 10 min with the cap open (keeping on MPC); do not over-dry!

105| Take reaction tube from MPC, add 18 µl Nuclease-free water by careful pipette mixing and incubate for 2 min.

106| Place the reaction tube onto the MPC, incubate for 2 min and pipette 15 µl of the DNA solution into a clean reaction tube.

107| Take reaction tube from MPC, add 15 µl Nuclease-free water by careful pipette mixing and incubate for 2 min.

108| Place the reaction tube onto the MPC, incubate for 2 min and pipette 15 µl of the DNA solution into the same reaction tube.

> PAUSE POINT *Store the completed library in a safe-lock tube at 4°C or continue directly with quality check.*

Quality check and quantification of library • TIMING approximately 3 h per 2 samples

109| Ensure the absence of too short or too long fragments, and of primer dimers using an Agilent Bioanalyzer High Sensitivity DNA assay (with the High Sensitivity DNA.xsy assay).

110| Continue directly with quantification of the library using the KAPA Library Quantification Kit for Illumina or Ion Torrent sequencing.

CAUTION Make sure to select the appropriate kit for the used sequencing platform and the real-time PCR cycler you use!

111| Completely thaw, mix, and briefly centrifuge all kit components.

112| Mastermix box: Before first use, prepare buffer qPCR/Primer mix from the Kit: Add 1 ml of Primer Premix (10×) to 5 ml bottle of KAPA SYBR FAST qPCR Master Mix (2×) and mix thoroughly. This mix is stable for 1 year when stored closed at -20°C.

113| Mastermix box: Prepare a mastermix using the following Table; calculate 6 reactions per DNA library plus 12 reactions for standards plus additional positive controls, if used, plus 2 no-template-controls (NTC) plus 0.5 reactions in excess.

**Component**	**1 x**	**x**
**Nuclease-free water**	4.0 µl	µl
**KAPA SYBR FAST qPCR Mastermix (including Primer mix)**	12.0 µl	µl
**Total volume**	16.0 µl	µl

114| Vortex and briefly centrifuge.

115| Mastermix box: Pipette 16 µl mastermix in each used well of a 96-well microtiter plate.

116| Template box: Dilute sample DNA only with dilution buffer: 3 dilutions with duplicates are needed. Keep sample DNA on MPC during pipetting to avoid transferring AMPure XP beads. Based on the Bioanalyzer data (see step 109), select adequate library dilutions (each 3 dilutions); use the following values as guidance:

- No detection with Bioanalyzer DNA HS Chip → use dilutions 1:20; 1:40; 1:80
- Weak signal with Bioanalyzer DNA HS Chip → use dilutions 1:200; 1:400; 1:800
- High signal with Bioanalyzer DNA HS Chip → use dilutions 1:2,000; 1:4,000; 1:8,000
- Amplified Libraries → use dilutions 1:20,000; 1:40,000; 1:80,000

117| Template box: Add 4 µl of diluted DNA library or standard to the respective wells, final reaction volume 20 µl.

118| Seal the plate with sealing film, press well on. Briefly centrifuge the plate using a microtiter plates centrifuge.

119| Insert the plate into the cycler and wipe the seal clean before closing the lid. Enter parameter, sample names and dilutions in the cycler software and start the KAPA PCR program.

	Step	Temperature	Time
1x	1	95°C	5 min
35x	2	95°C	30 sec
	3	60°C	45 sec
1x	4	4°C	∞
1x	Melting curve analysis		

120| After completion of the run, check the integrity and plausibility of the PCR results, e.g. fluorescence intensity, amplification curves, melting curves, R^2 of the standard curve ≥ 0.95, PCR efficiency ≥ 0.95, efficiencies between 0.90 and 0.95 are acceptable if R^2 is ≥ 0.99, Cq values of different sample dilutions and the positive control (Illumina).

Calculate DNA library molarities from the qPCR results and optionally pool several libraries with different barcode adapters for one sequencing run.

Timing

The time is calculated for 2 samples.

Steps 1-10, disintegration of sample material: 30 min

Steps 11-27, extraction of RNA: 2 h

Option A, RNA extraction of ticks (*optional to steps 1-17*): 1-2 h

Option B, RNA extraction of mosquitoes (*optional to steps 1-17*): 1-2 h

Steps 28-29, quantification and integrity check of RNA: 1 h

Steps 30-38, concentrating RNA (*optional, if RNA concentration is too low*): 45 min

Steps 39-47, cDNA synthesis: 4 h

Steps 48-50, library preparation: fragmentation: 30 min

Steps 51-77, (*optional to steps 78-92*), **manual library preparation for sequencing with Ion Torrent PGM: 3 h**

Steps 51-58, purification and concentration of fragmented cDNA synthesis products: 45 min

Steps 59-63, library preparation for sequencing with Ion Torrent PGM, end-repair: 45 min

Steps 64-68, library preparation for sequencing with Ion Torrent PGM, adapter ligation: 30 min

Steps 69-77, purification and concentration of Ion Torrent Library: 45 min

Steps 78-92 (*optional to steps 51-77*), **automated library preparation for sequencing with Illumina MiSeq: 4 hours**

Steps 93-108, size selection of library: 45 min

Steps 109-120, quality check and quantification of library: 3 h

Troubleshooting

Step	Problem	Possible reason	Solution
28	Very low RNA concentration is measured	Depends on the sample	Repeat RNA extraction with sufficient material if possible and eluate with nuclease-free water supplied with the kit,<br>Concentrate RNA (steps 31-38) before cDNA synthesis
29	RNA is fragmented and RIN value is low or not calculated	Depends on the sample and its pre-treatment (e.g., processing, fixation)	Proceed with the protocol
109	Size distribution is out of the suitable range	AMPure beads are not previously calibrated	Carefully repeat the size selection procedure with calibrated beads,<br>Check residual Ion Torrent library from step 61 and 77 using Agilent HS chip to follow the library status

			Check sample before (step 61) and after library preparation (step 77) using HS chip to find the possible error source
109	No DNA detected	Very low initial input for library preparation	Proceed with library quantification by qPCR. Use expected fragment size for KAPA calculation
120	No library determined	Improper library dilutions used for qPCR or very low initial input for library preparation	Repeat KAPA qPCR with appropriate dilutions, use DNA dilution buffer
120	Lower amount of library determined than expected	Improper library dilutions used for qPCR or very low initial input for library preparation	If measurable amount of library is detected, proceed with sequencing

Anticipated results

Step 29. Typical results of the Bioanalyzer 2100 RNA pico assay are shown in **Figure 3**. In lanes marked with C intact total RNA is evident from rRNA peaks. In lanes marked with T (**Fig. 3, A+B**) and M (**Fig. 3, C**), rRNA peaks are partly or completely missing because of degradation of RNA. The *Bacillus subtilis* samples (spore) are also shown as electropherograms (**Fig. A1**). The cryoPrep sample shows the most intact RNA whereas the TissueLyser sample shows very degraded RNA. From the generated electropherogram, the software calculates an RNA Integrity Number (RIN) that can help in assessing RNA quality. According to our experience, however, there is no correlation between pathogen detection or sequence data output and the RIN value.

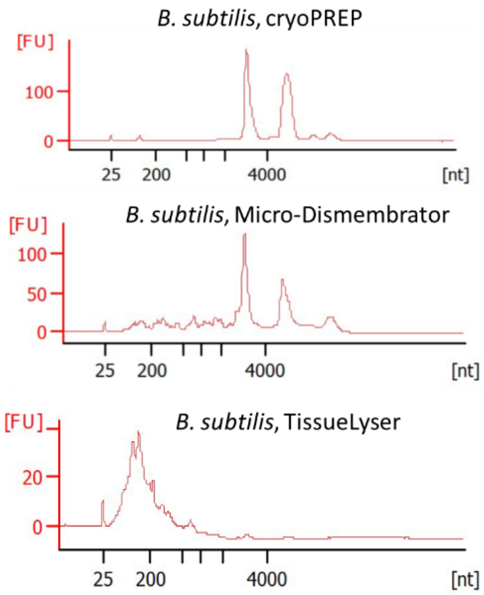

Figure A1 RNA quality after RNA extraction on RNA 6000 Pico Assay (Agilent) for the *Bacillus subtilis* spore sample using different disintegration tools. nt, nucleotides.

In some cases, the extracted RNA had only a low concentration and needed to be concentrated (using steps 31-38 of the protocol) before cDNA synthesis. This was done for the bird feces, tap water and the pizza samples. Compare Fig. A3 for resulting libraries.

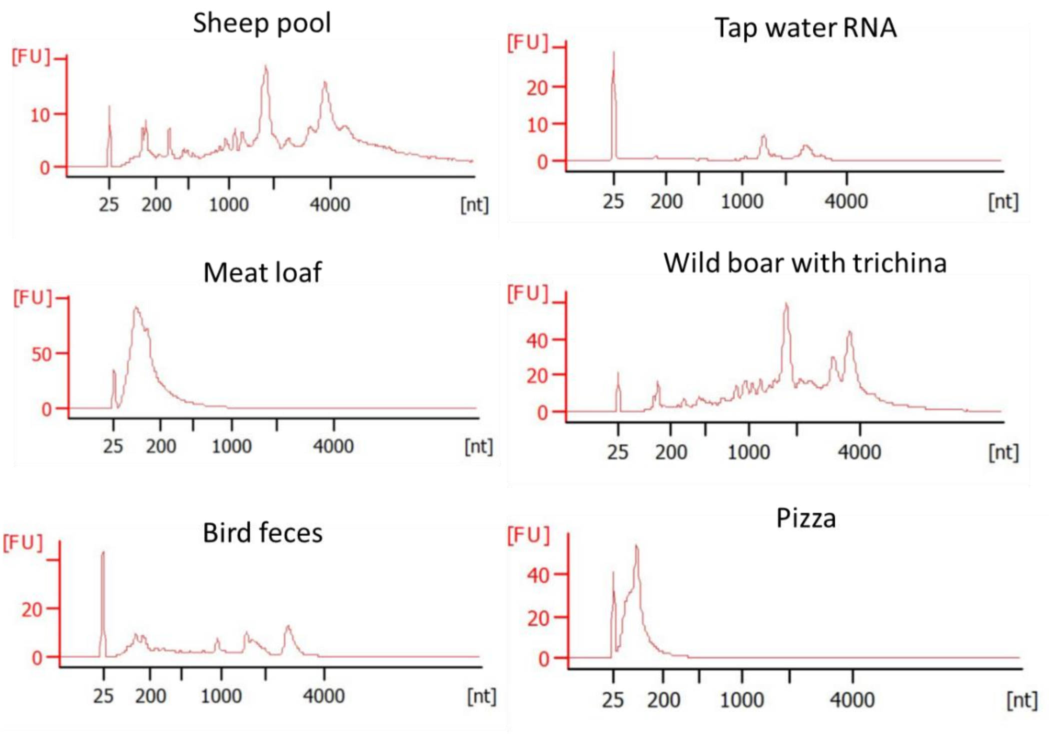

Figure A2 RNA quality after RNA extraction on RNA 6000 Pico Assay (Agilent) for different samples disintegrated with the cryoPREP before the RNA concentration step. Compare Fig. A3 for the resulting DNA libraries. nt, nucleotides.

Step 109. The size-selected library is composed of fragments ranging from 300 to 1,000 bp with a peak at about 500-550 bp. Ideally, the library should show a representative or weak peak (**Fig. A3**, sheep pool ID 1454, containing an astrovirus[4], or wild boar with trichina ID 1806, respectively). For the trichina-sample, we obtained 0.02% trichinellid reads (data set of 1,627,077 reads). But also in cases without a peak, virus reads can be detected depending on the viral load. This was for example the case in the meat loaf sample ID 1488 (**Fig. A3**) that contained 319 reads (0.1%) of a pepper mild mottle virus 6.36 kb genome (99% identity, RNA virus) representing a plant pathogen (total data set 360,148 reads).

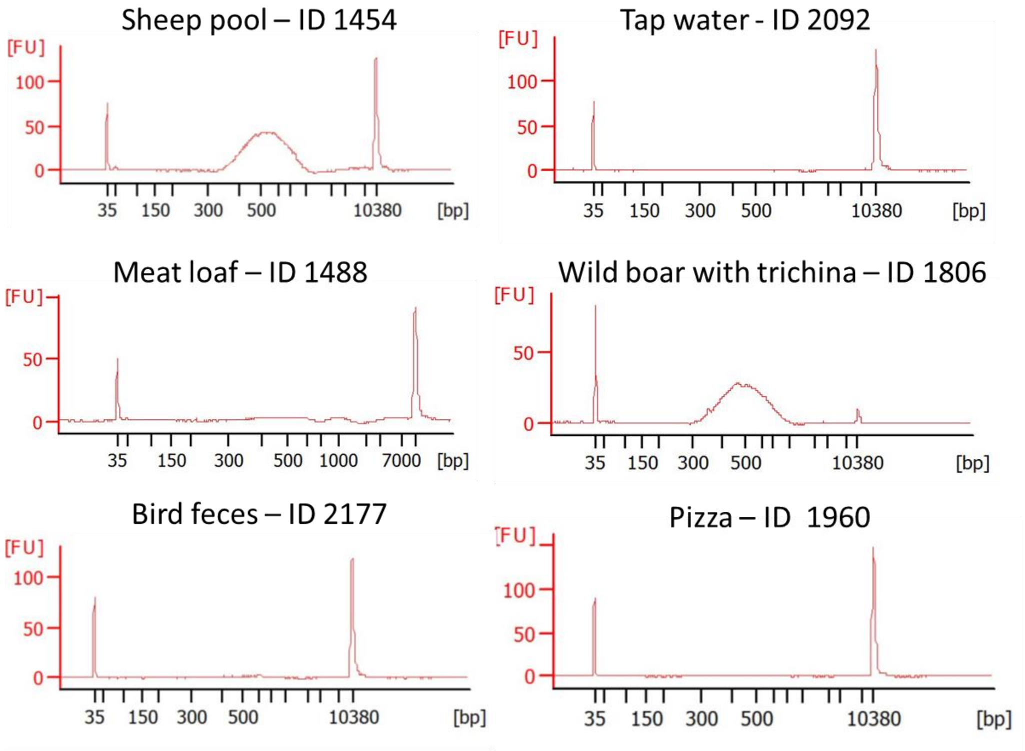

Figure A2 Sequencing libraries visualized with a High Sensitivity DNA Assay (Agilent) for sequencing with Illumina (squirrel transudate[5]) and Ion Torrent PGM (sheep pool[4], meat loaf, meat with trichina). Compare Fig. A2 for the quality of RNA. bp, base pairs.

Step 120. After completion of the run, check fluorescence intensity, amplification curves, melting curves for integrity and plausibility of the PCR results. The relative standard deviation of replicates should be < 10% and R^2 of the standard curve ≥ 0.95, PCR efficiency ≥ 0.95, efficiencies between 0.90 and 0.95 are acceptable if R^2 is ≥ 0.99. Check also the Cq values of different sample dilutions for plausibility.

Additional references

1. GS FLX Titanium General Library Preparation Method Manual, Document USM-00048.B, Roche Diagnostics, April 2009.
2. GS FLX System Technical Bulletin TCB No. 017-2009, Roche Diagnostics, November 2009.
3. Troubleshooting. In: ND-1000 Spectrophotometer V3.5 User's Manual. NanoDrop Technologies, Inc. Rev 6/2007
4. Pfaff F, Schlottau K, Scholes S, Courtenay A, Hoffmann B, Höper D, Beer M. A novel astrovirus associated with encephalitis and ganglionitis in domestic sheep. Transbound Emerg 2017; 64:677–682.

Supplementary file 2: Extractions of the RIEMS result protocols graphically summarizing the 100 most abundant families found in the sequencing data sets of samples given in Tables 2 and 3 of the main document, ordered by library IDs. In order to represent all detected superkingdoms, the displayed families are selected from the complete result applying the HighestAverages algorithm from the R package SciencesPo. Families are sorted alphabetically, color-coded according to superkingdoms. Note that the graphs are in log-scale. The original sample name created by the sequencing platform is included for each data set that refers to the Library IDs given in Table 2 and 3. For example, the name "lib126-11-reg2-RL2-run2011-11-02-resultprotocol.pdf" refers to library ID 126.

Known false positive viral families in the graphs are listed in the legends. Causes for the occurrence of false positives may be (i) reagent contamination and taxonomic misclassification (Retroviridae, Arenaviridae) and (ii) missorting of reads during data deconvolution or run-to-run carry-over. The former workflow-specific false positives are labelled WF (workflow), the latter where the taxonomic families are known to emerge from other samples in the same run/previous run are labeled RS (run specific). For details on the possible causes of false positives, please refer to the main text.

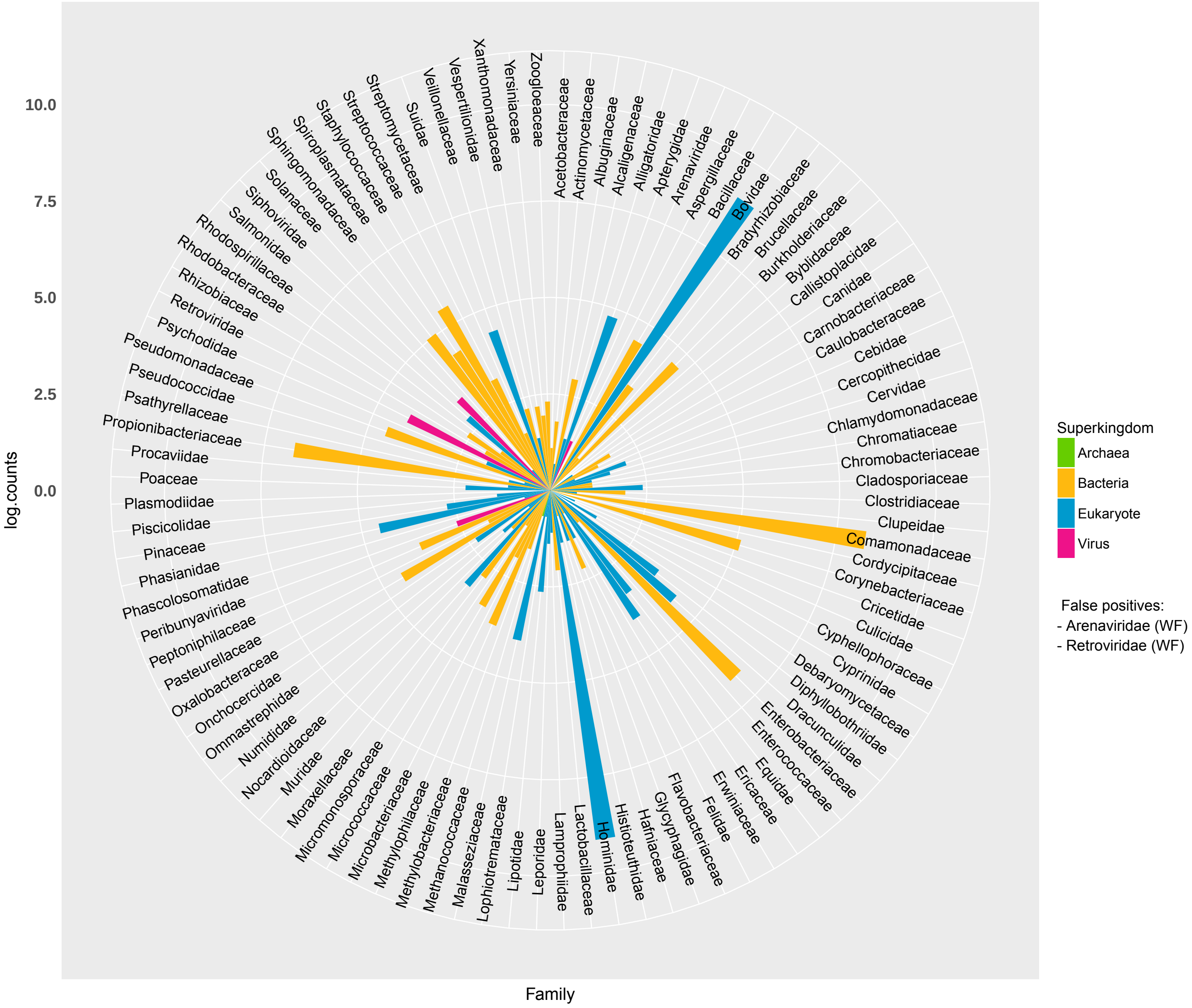

Figure 3: Graphical representation of read counts for the 100 most abundant families. In order to represent all detected superkingdoms, the displayed families are selected from the complete result applying the HighestAverages algorithm from the R package SciencesPo. Families are sorted alphabetically, color-coded according to superkingdoms. Note that the graph is in log-scale.

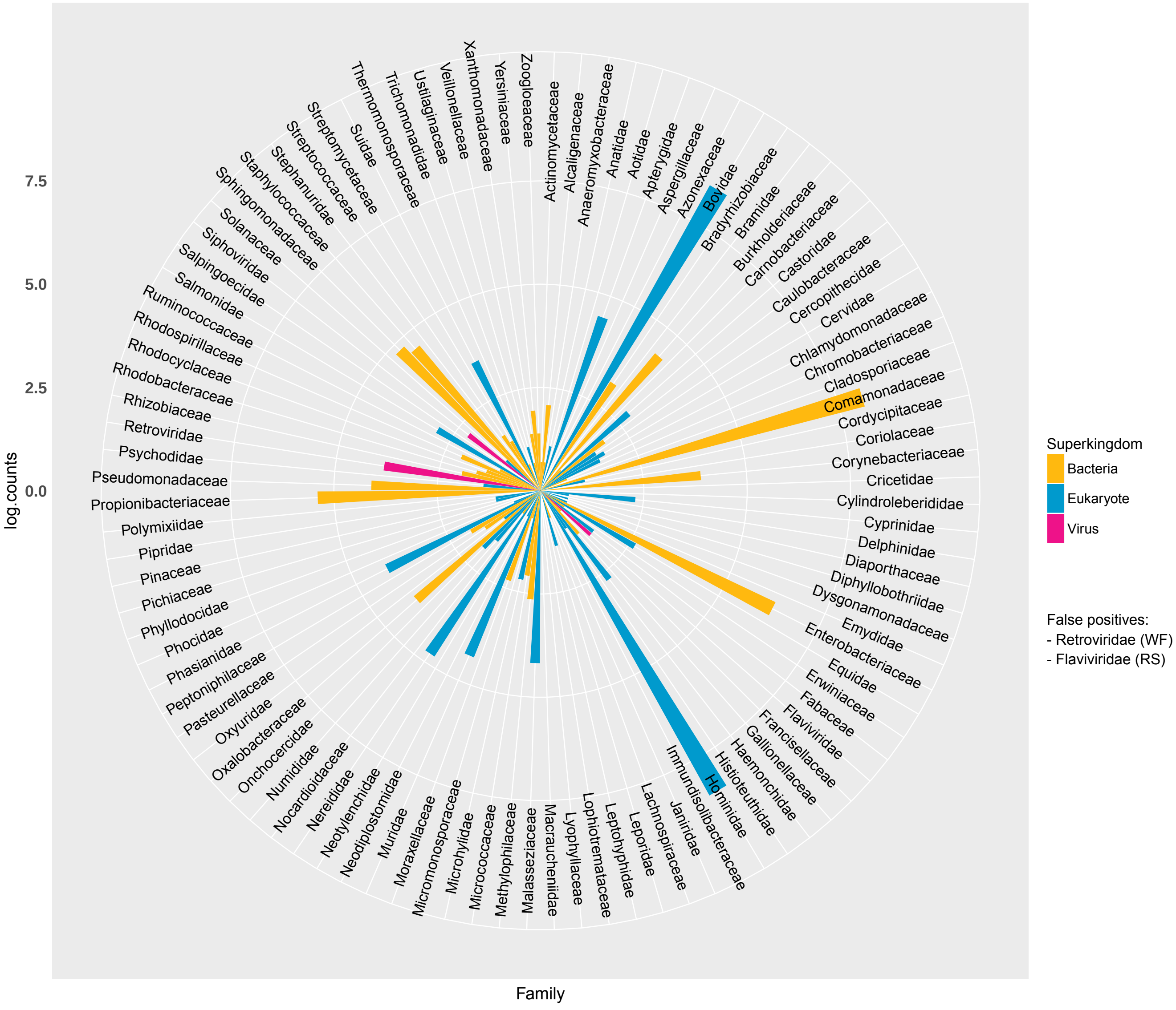

Figure 3: Graphical representation of read counts for the 100 most abundant families. In order to represent all detected superkingdoms, the displayed families are selected from the complete result applying the HighestAverages algorithm from the R package SciencesPo. Families are sorted alphabetically, color-coded according to superkingdoms. Note that the graph is in log-scale.

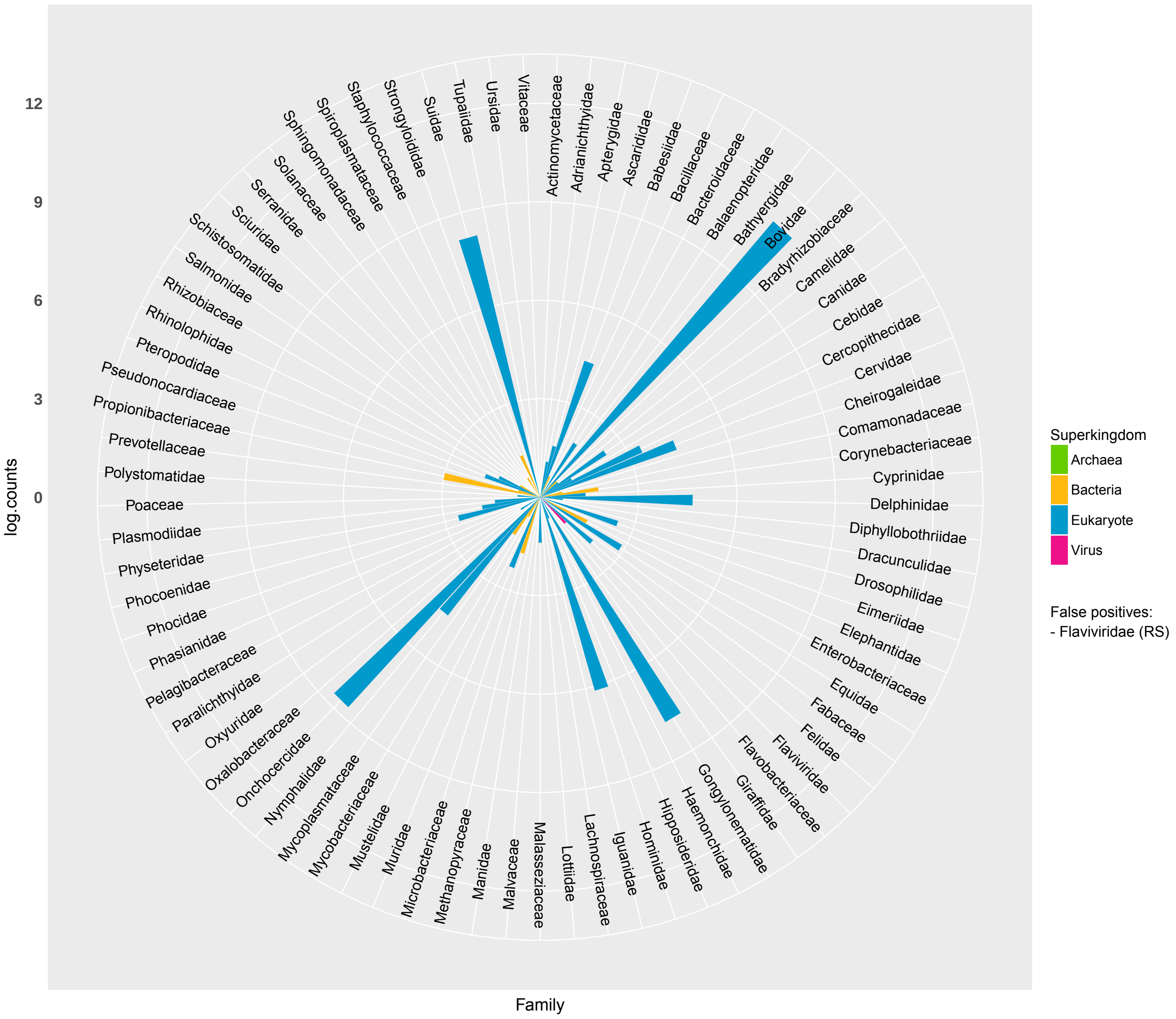

Figure 3: Graphical representation of read counts for the 100 most abundant families. In order to represent all detected superkingdoms, the displayed families are selected from the complete result applying the HighestAverages algorithm from the R package SciencesPo. Families are sorted alphabetically, color-coded according to superkingdoms. Note that the graph is in log-scale.

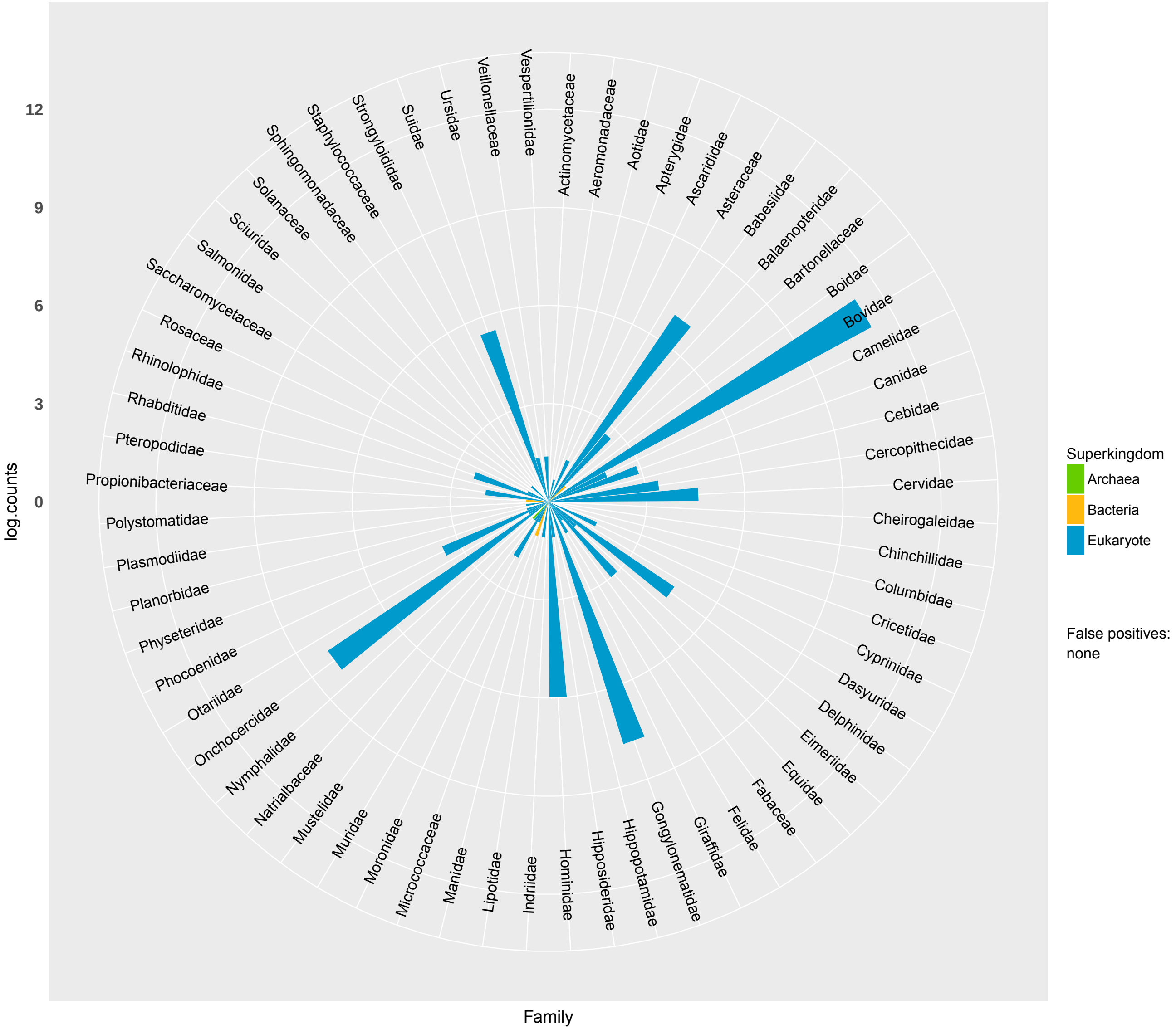

Figure 3: Graphical representation of read counts for the 100 most abundant families. In order to represent all detected superkingdoms, the displayed families are selected from the complete result applying the HighestAverages algorithm from the R package SciencesPo. Families are sorted alphabetically, color-coded according to superkingdoms. Note that the graph is in log-scale.

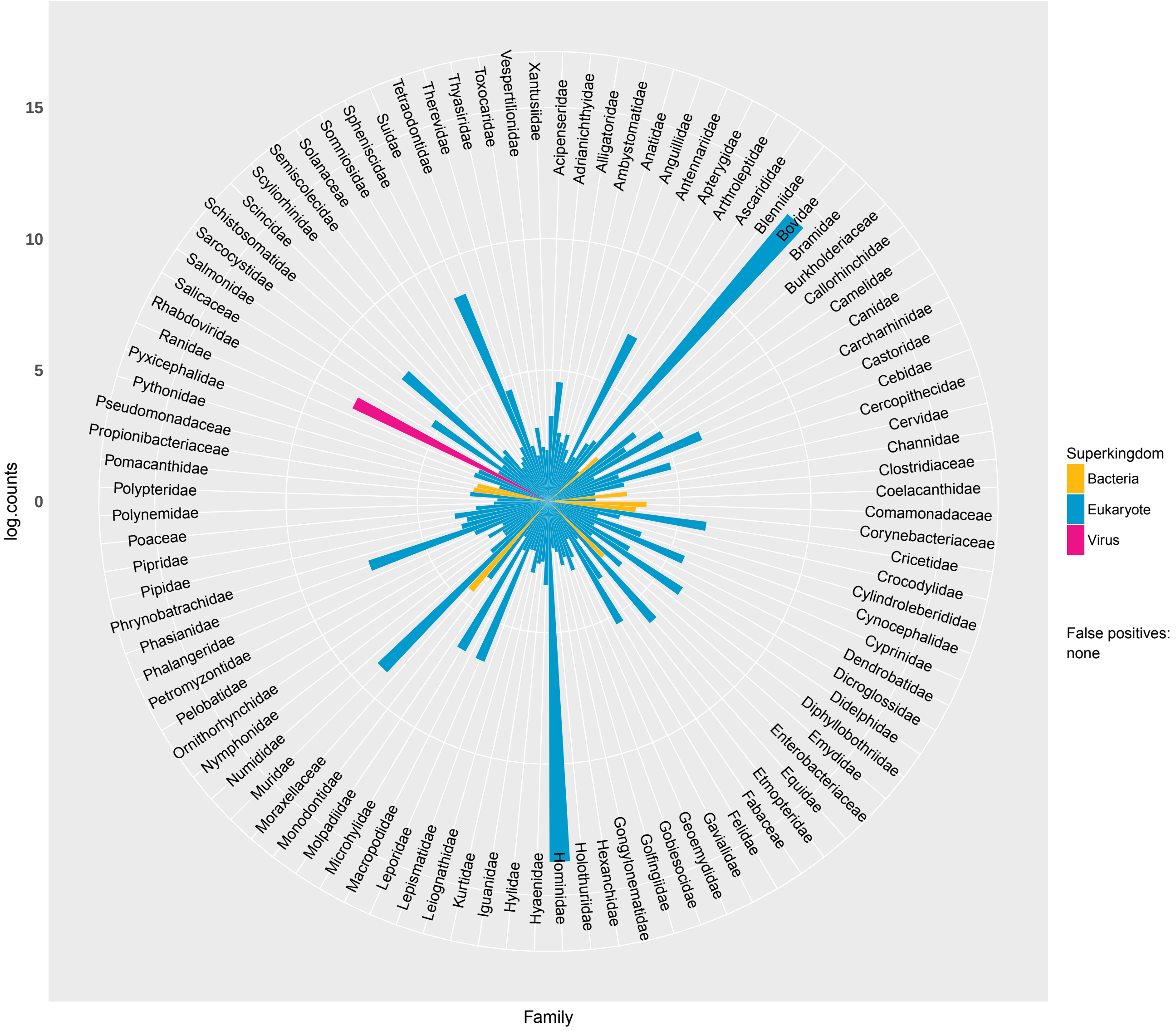

Figure 3: Graphical representation of read counts for the 100 most abundant families. In order to represent all detected superkingdoms, the displayed families are selected from the complete result applying the HighestAverages algorithm from the R package SciencesPo. Families are sorted alphabetically, color-coded according to superkingdoms. Note that the graph is in log-scale.

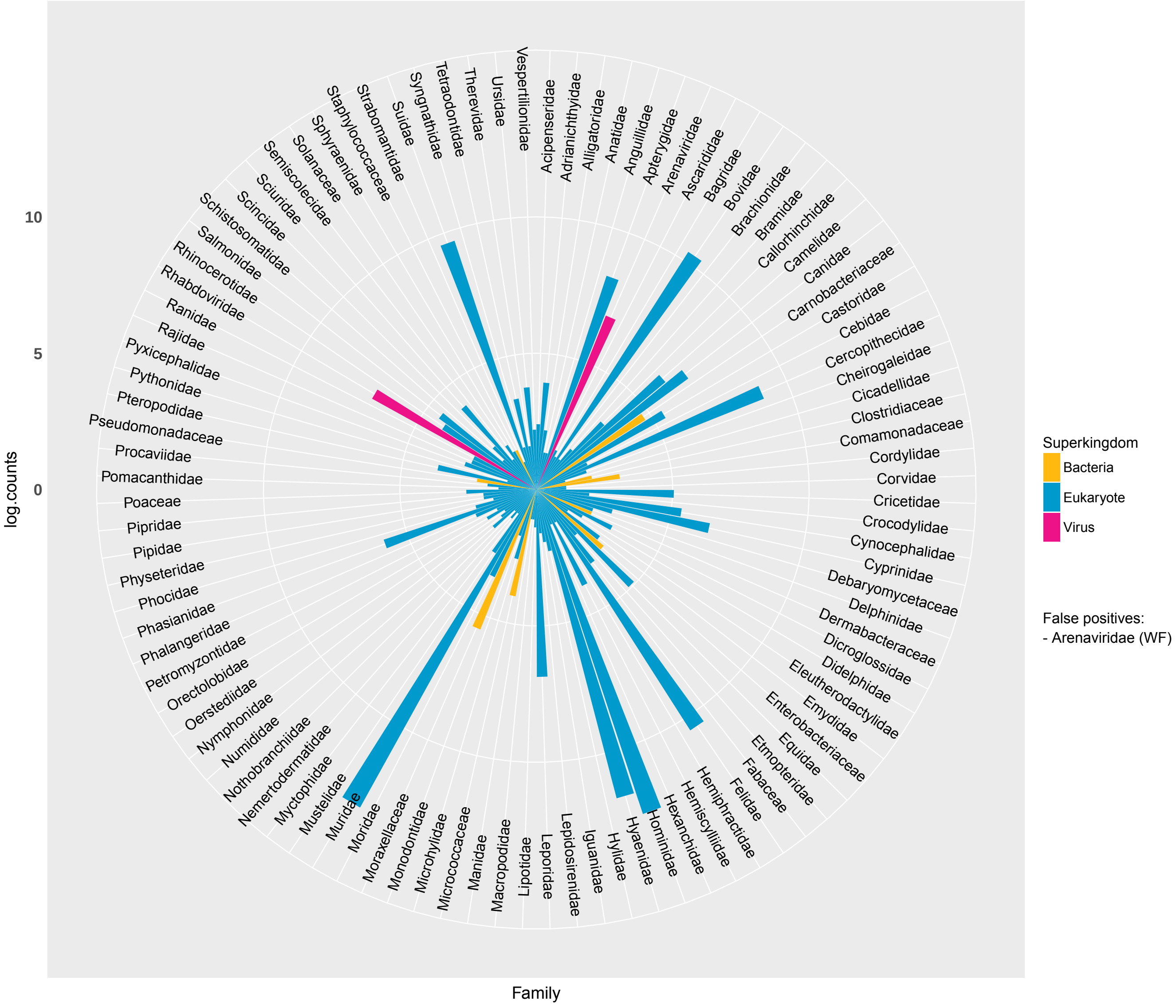

Figure 3: Graphical representation of read counts for the 100 most abundant families. In order to represent all detected superkingdoms, the displayed families are selected from the complete result applying the HighestAverages algorithm from the R package SciencesPo. Families are sorted alphabetically, color-coded according to superkingdoms. Note that the graph is in log-scale.

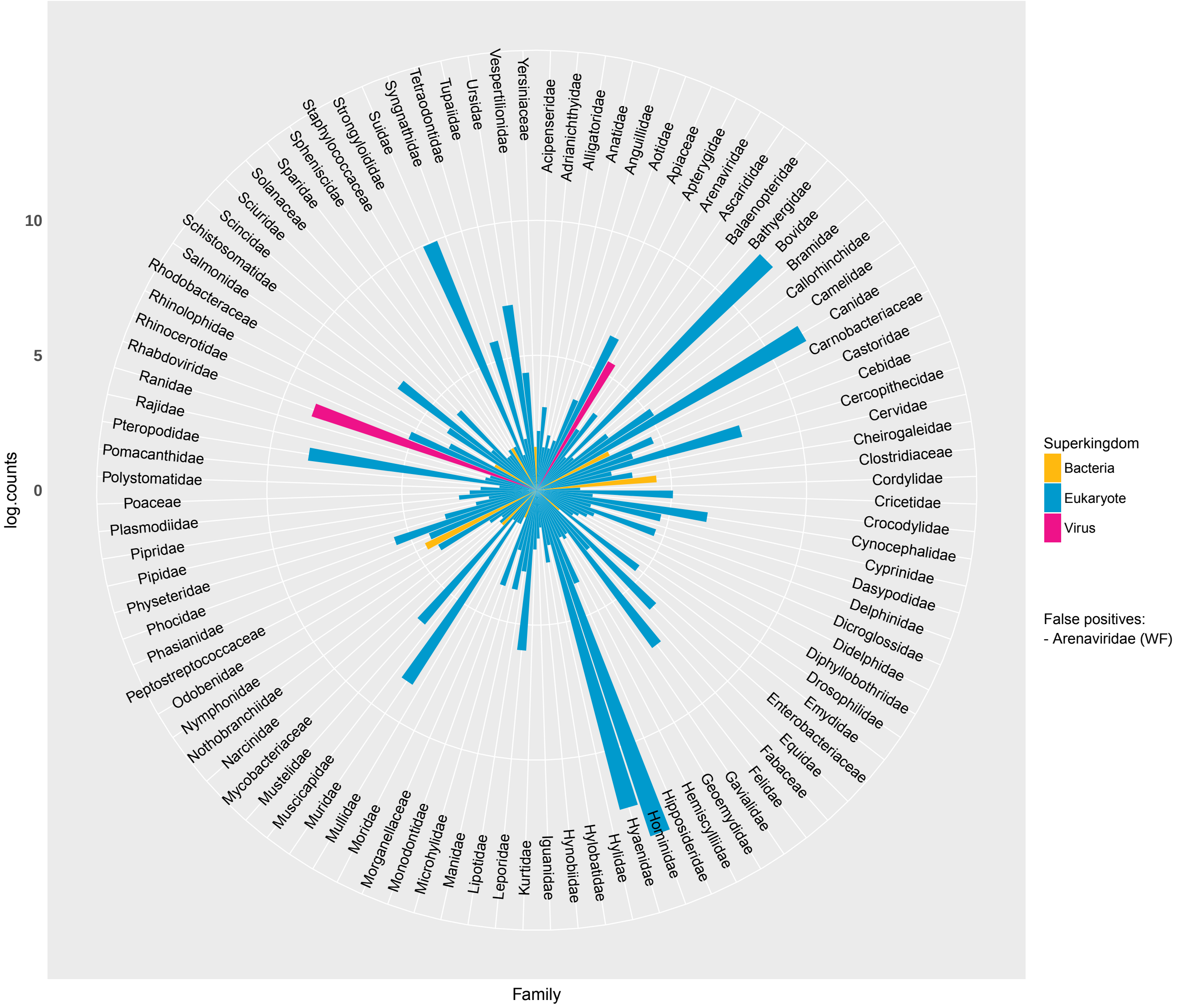

Figure 3: Graphical representation of read counts for the 100 most abundant families. In order to represent all detected superkingdoms, the displayed families are selected from the complete result applying the HighestAverages algorithm from the R package SciencesPo. Families are sorted alphabetically, color-coded according to superkingdoms. Note that the graph is in log-scale.

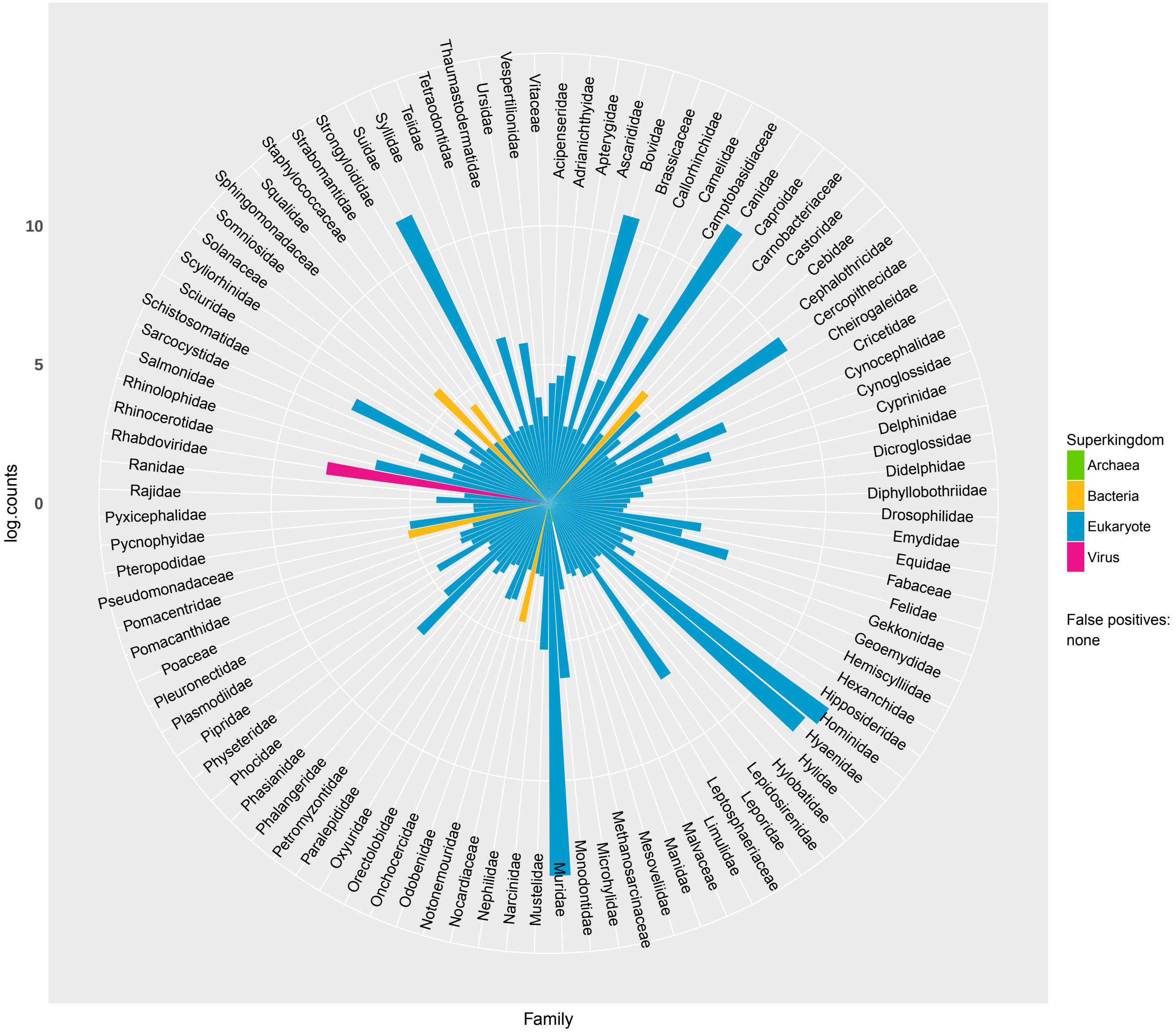

Figure 3: Graphical representation of read counts for the 100 most abundant families. In order to represent all detected superkingdoms, the displayed families are selected from the complete result applying the HighestAverages algorithm from the R package SciencesPo. Families are sorted alphabetically, color-coded according to superkingdoms. Note that the graph is in log-scale.

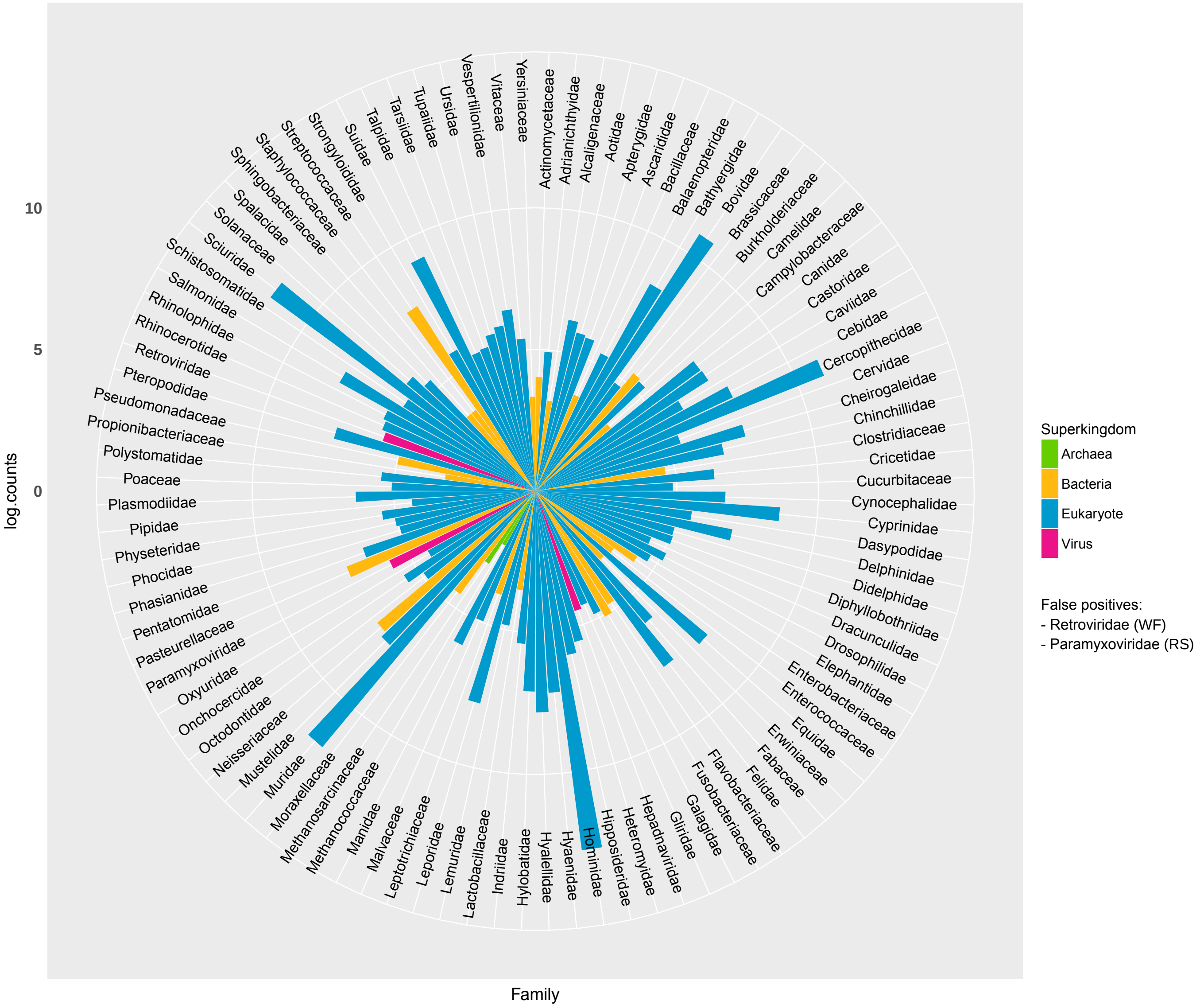

Figure 3: Graphical representation of read counts for the 100 most abundant families. In order to represent all detected superkingdoms, the displayed families are selected from the complete result applying the HighestAverages algorithm from the R package SciencesPo. Families are sorted alphabetically, color-coded according to superkingdoms. Note that the graph is in log-scale.

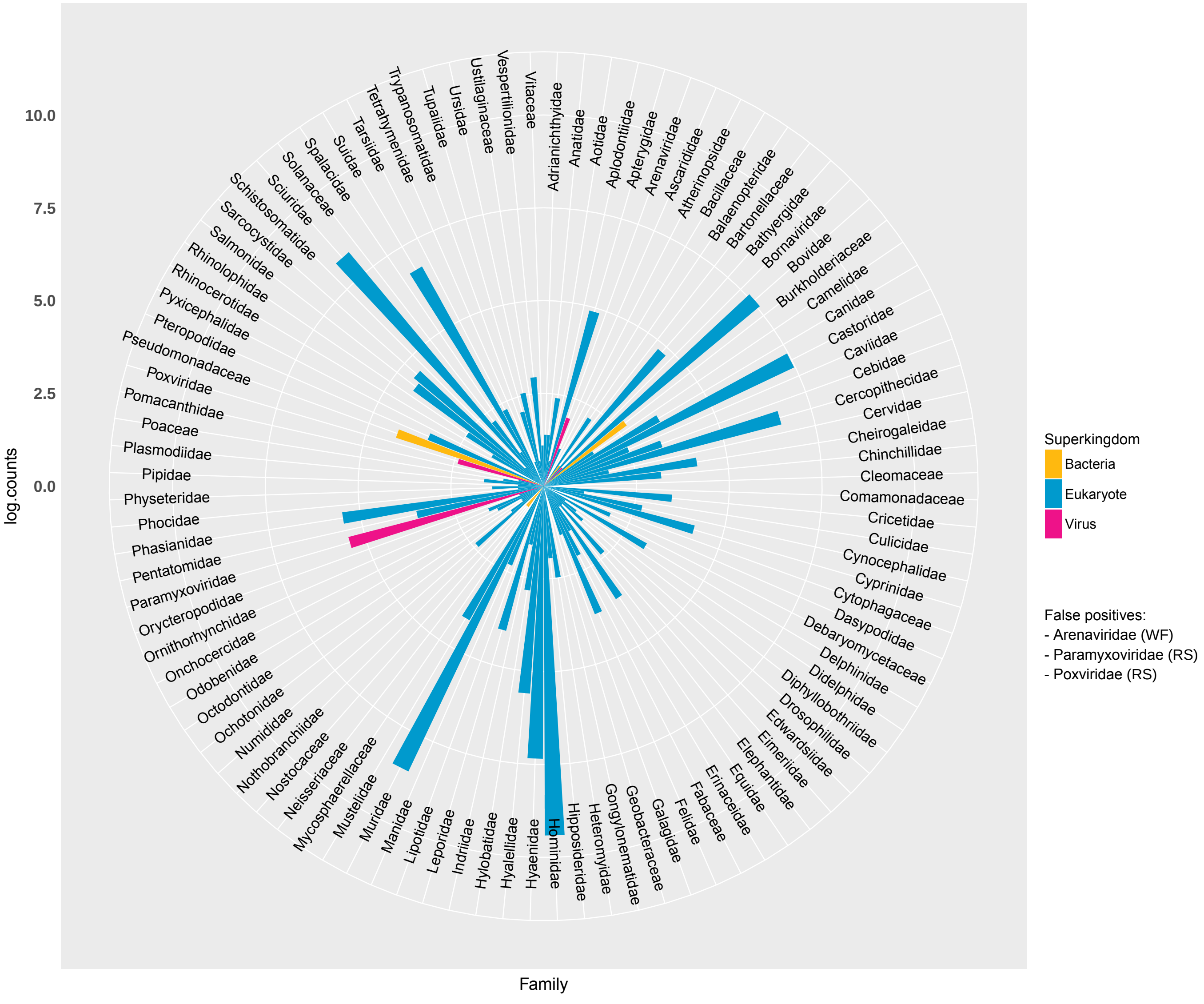

Figure 3: Graphical representation of read counts for the 100 most abundant families. In order to represent all detected superkingdoms, the displayed families are selected from the complete result applying the HighestAverages algorithm from the R package SciencesPo. Families are sorted alphabetically, color-coded according to superkingdoms. Note that the graph is in log-scale.

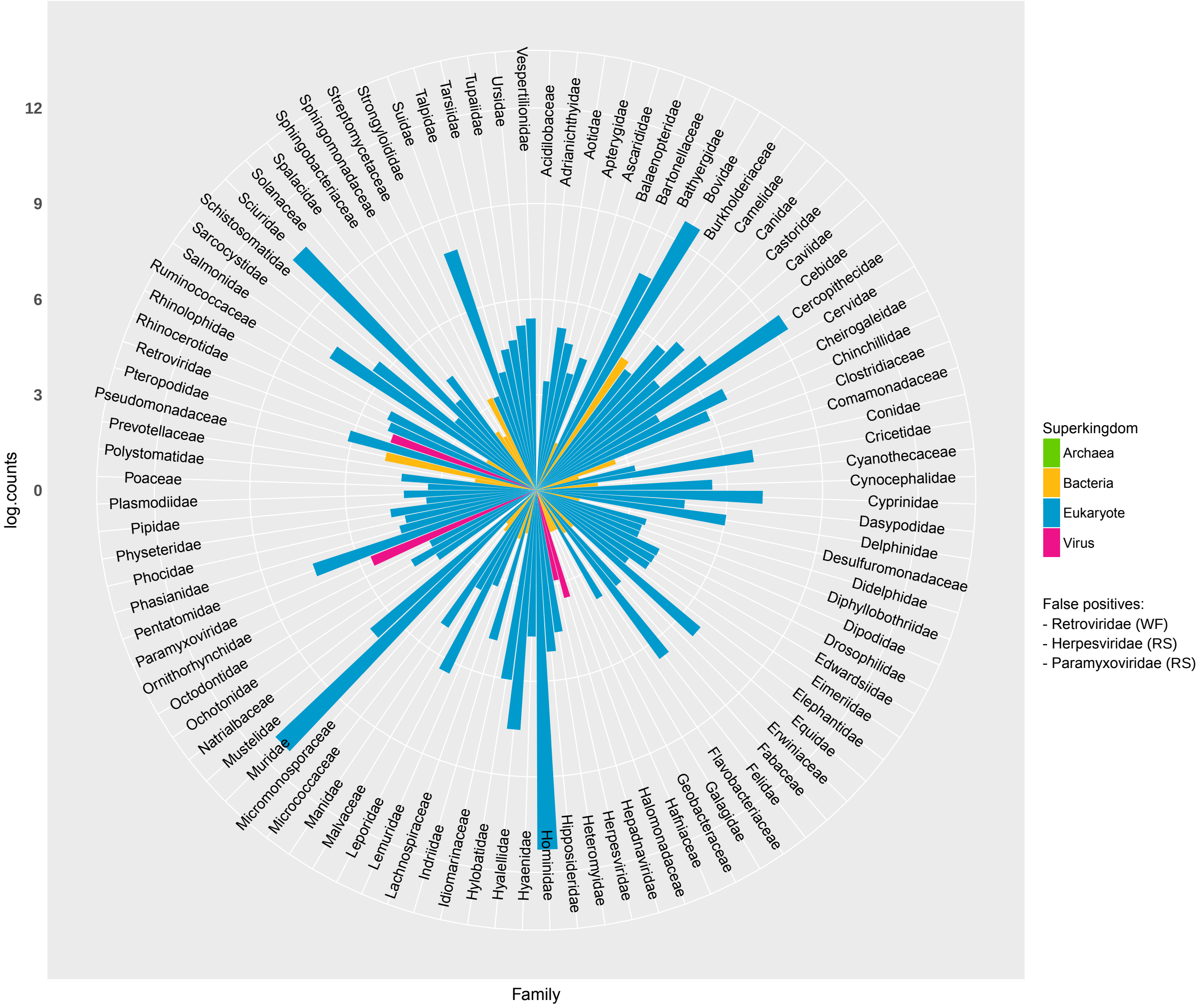

Figure 3: Graphical representation of read counts for the 100 most abundant families. In order to represent all detected superkingdoms, the displayed families are selected from the complete result applying the HighestAverages algorithm from the R package SciencesPo. Families are sorted alphabetically, color-coded according to superkingdoms. Note that the graph is in log-scale.

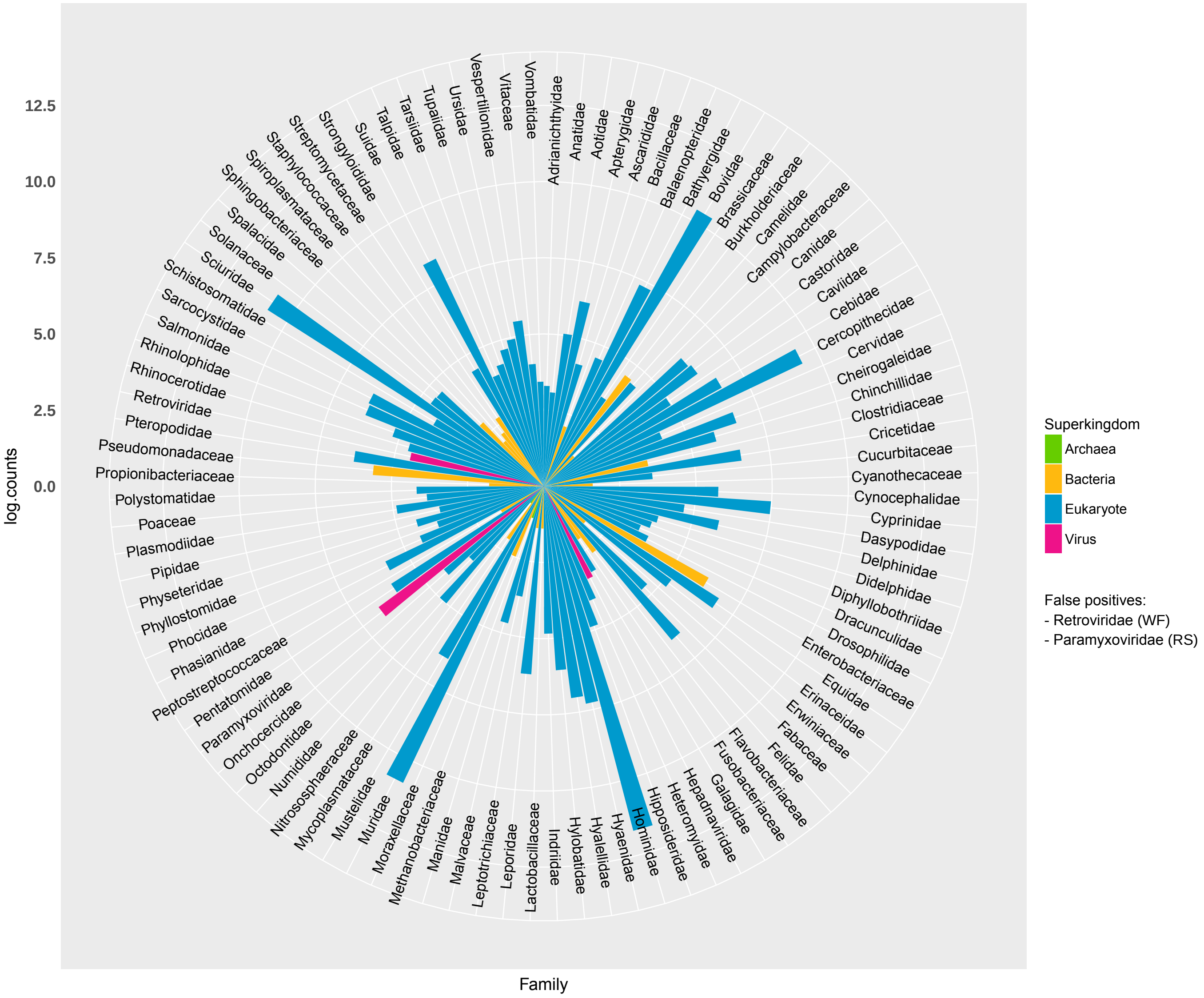

Figure 3: Graphical representation of read counts for the 100 most abundant families. In order to represent all detected superkingdoms, the displayed families are selected from the complete result applying the HighestAverages algorithm from the R package SciencesPo. Families are sorted alphabetically, color-coded according to superkingdoms. Note that the graph is in log-scale.

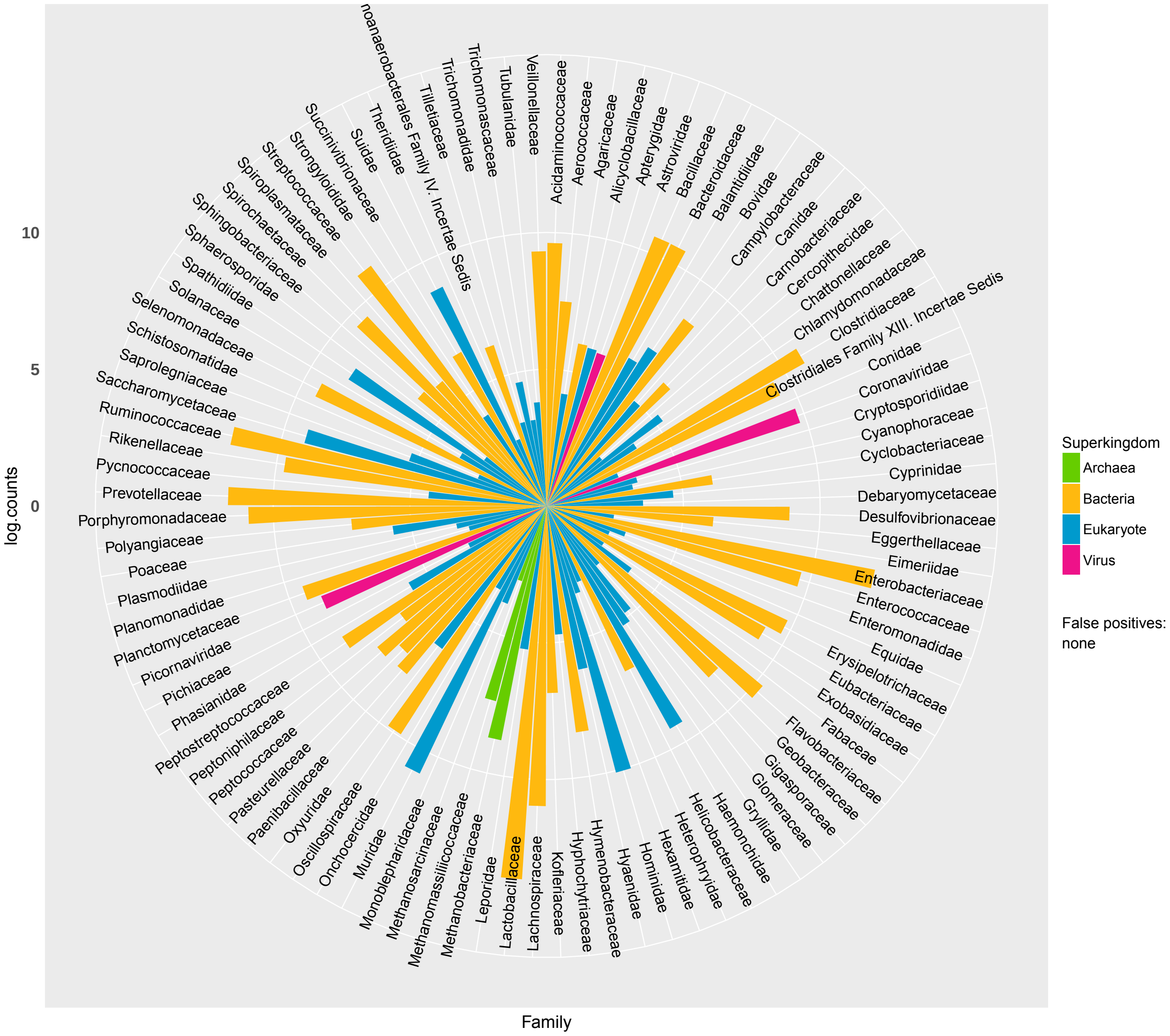

Figure 3: Graphical representation of read counts for the 100 most abundant families. In order to represent all detected superkingdoms, the displayed families are selected from the complete result applying the HighestAverages algorithm from the R package SciencesPo. Families are sorted alphabetically, color-coded according to superkingdoms. Note that the graph is in log-scale.

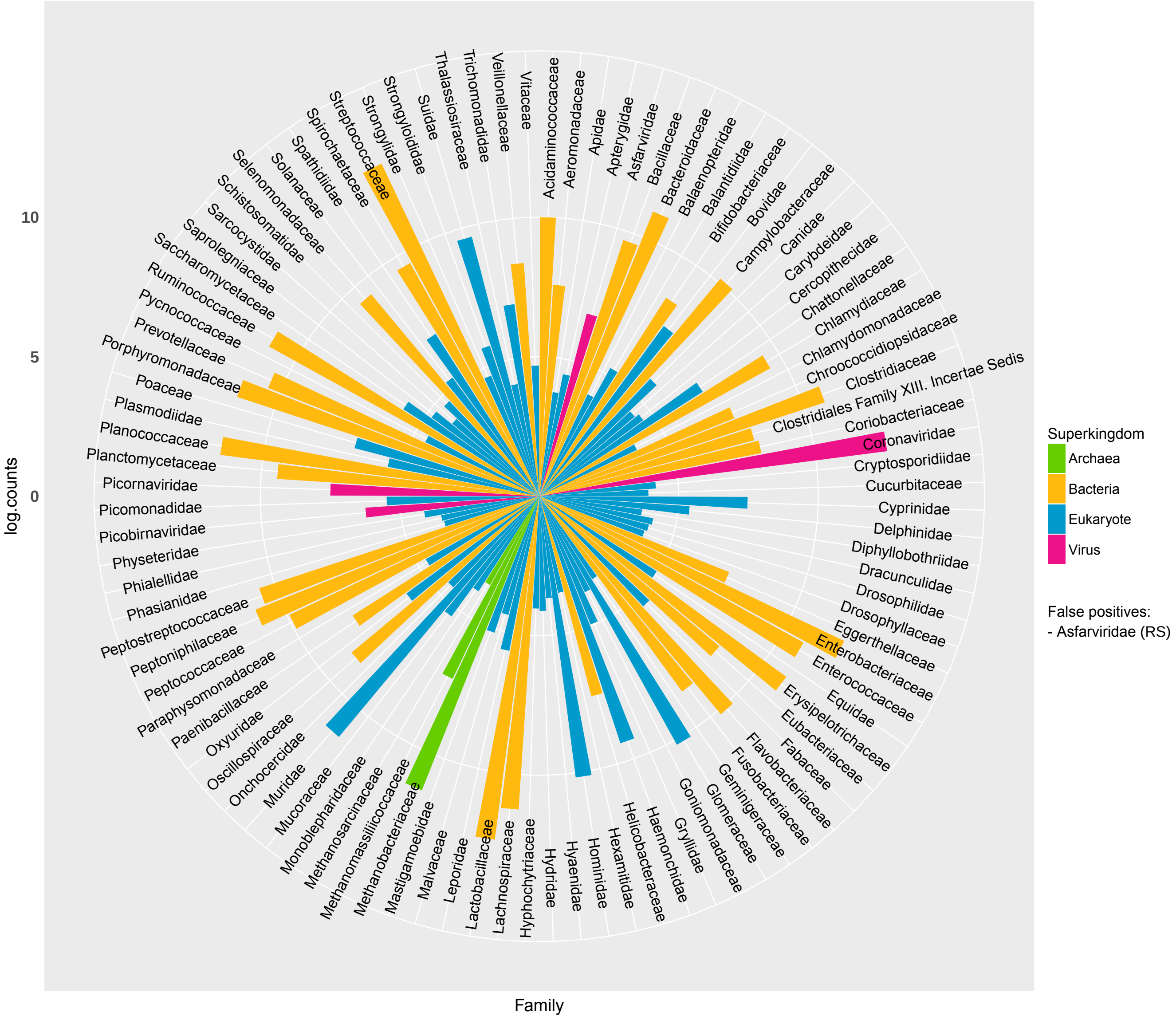

Figure 3: Graphical representation of read counts for the 100 most abundant families. In order to represent all detected superkingdoms, the displayed families are selected from the complete result applying the HighestAverages algorithm from the R package SciencesPo. Families are sorted alphabetically, color-coded according to superkingdoms. Note that the graph is in log-scale.

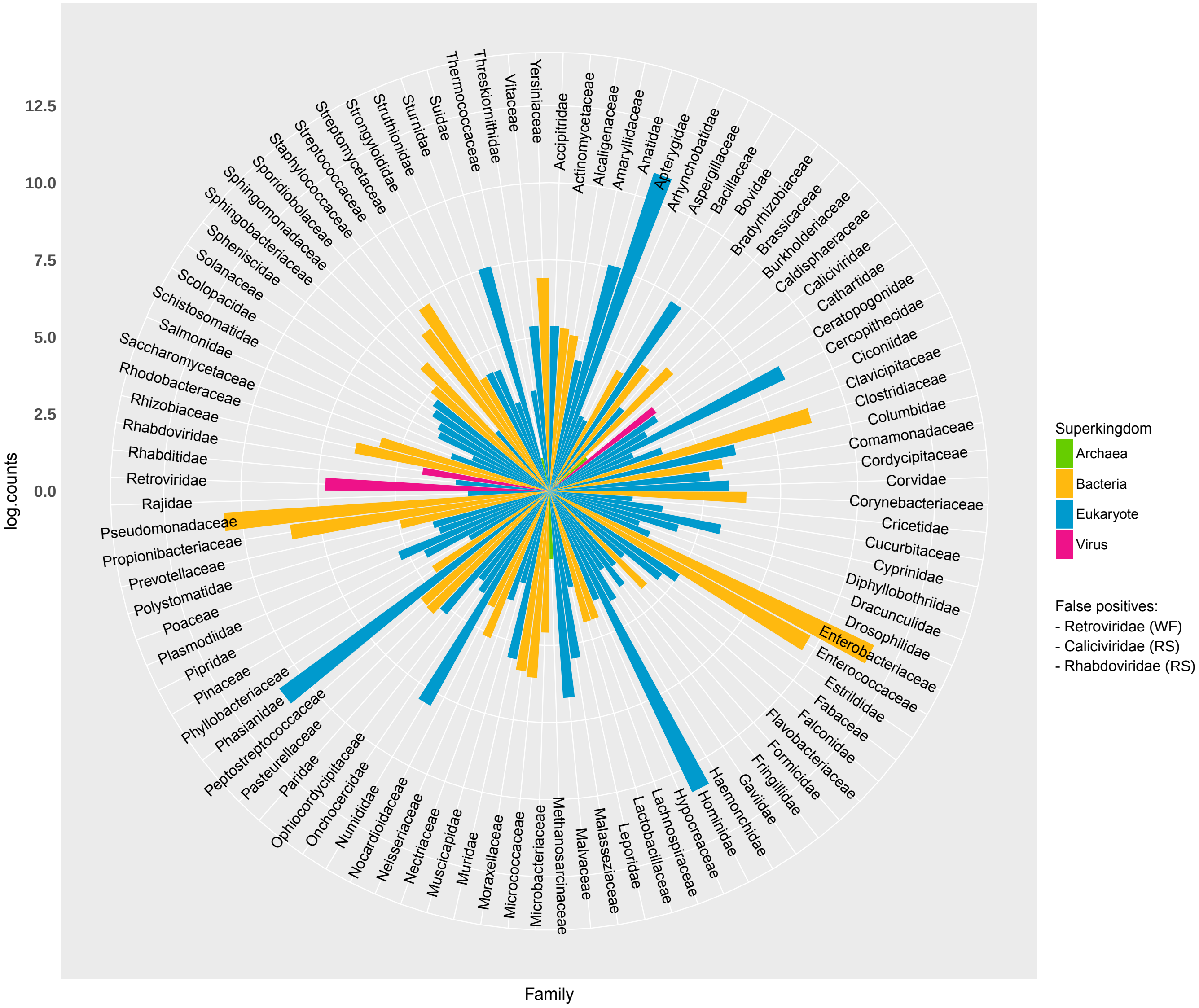

Figure 3: Graphical representation of read counts for the 100 most abundant families. In order to represent all detected superkingdoms, the displayed families are selected from the complete result applying the HighestAverages algorithm from the R package SciencesPo. Families are sorted alphabetically, color-coded according to superkingdoms. Note that the graph is in log-scale.

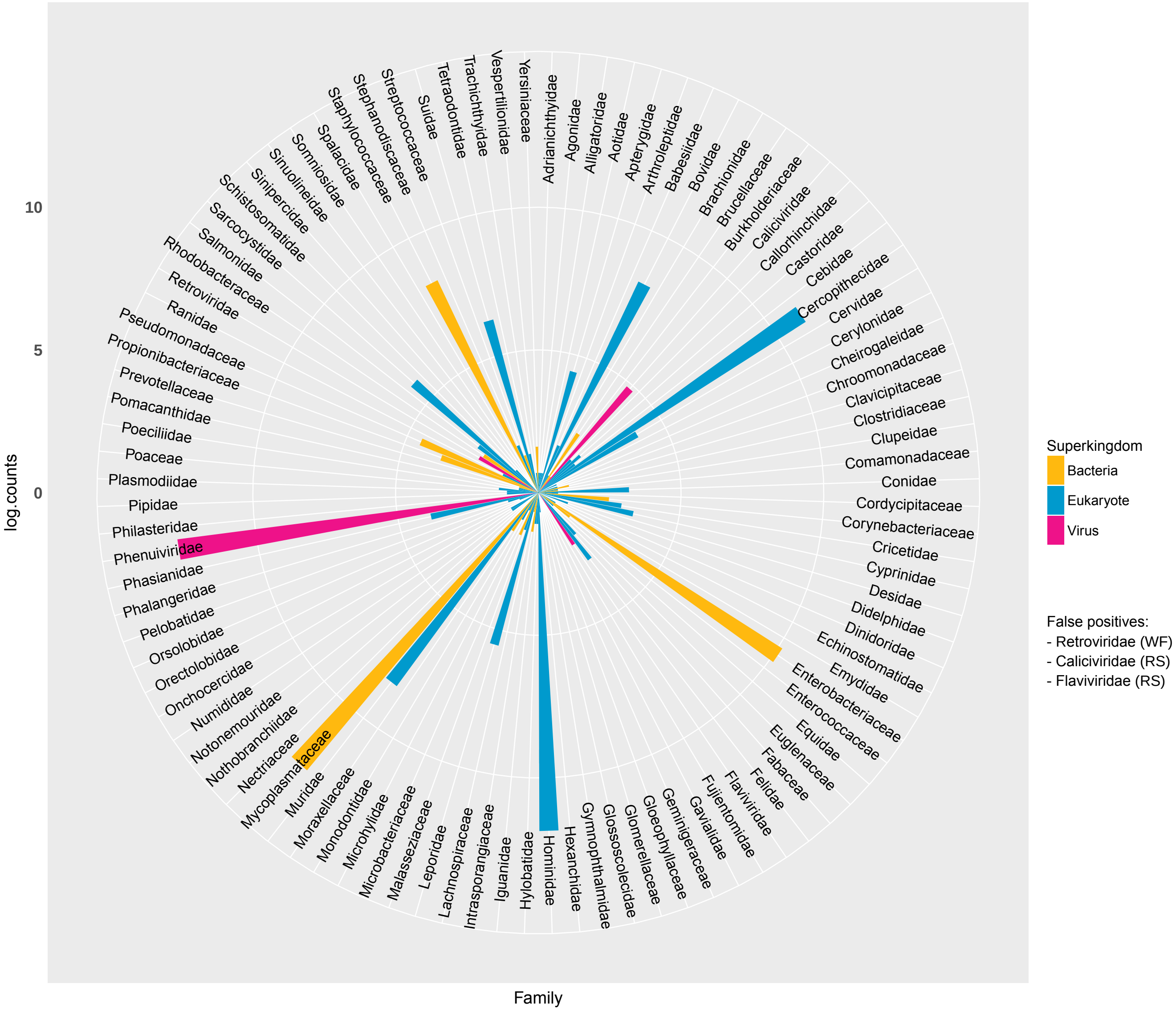

Figure 3: Graphical representation of read counts for the 100 most abundant families. In order to represent all detected superkingdoms, the displayed families are selected from the complete result applying the HighestAverages algorithm from the R package SciencesPo. Families are sorted alphabetically, color-coded according to superkingdoms. Note that the graph is in log-scale.

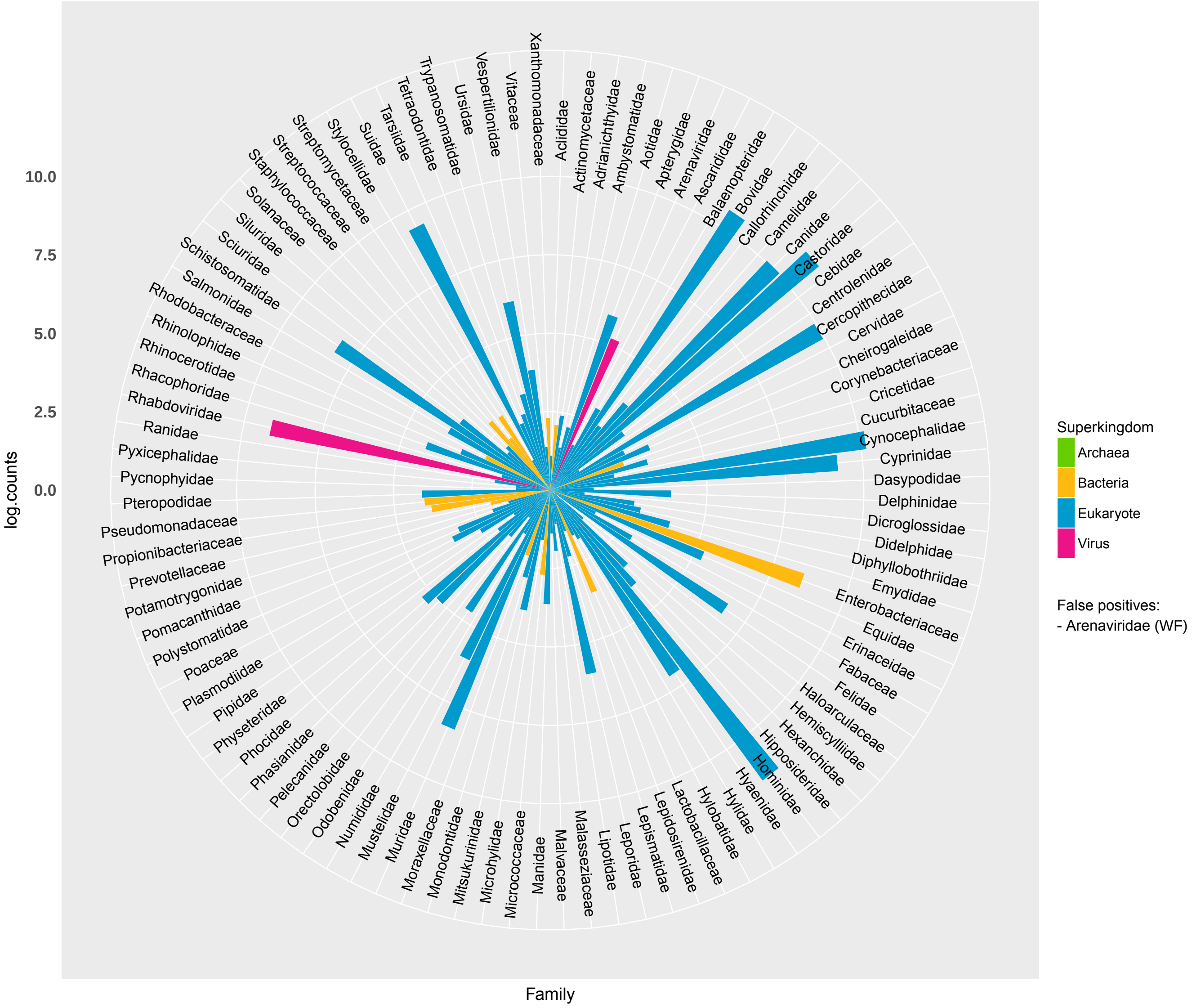

Figure 3: Graphical representation of read counts for the 100 most abundant families. In order to represent all detected superkingdoms, the displayed families are selected from the complete result applying the HighestAverages algorithm from the R package SciencesPo. Families are sorted alphabetically, color-coded according to superkingdoms. Note that the graph is in log-scale.

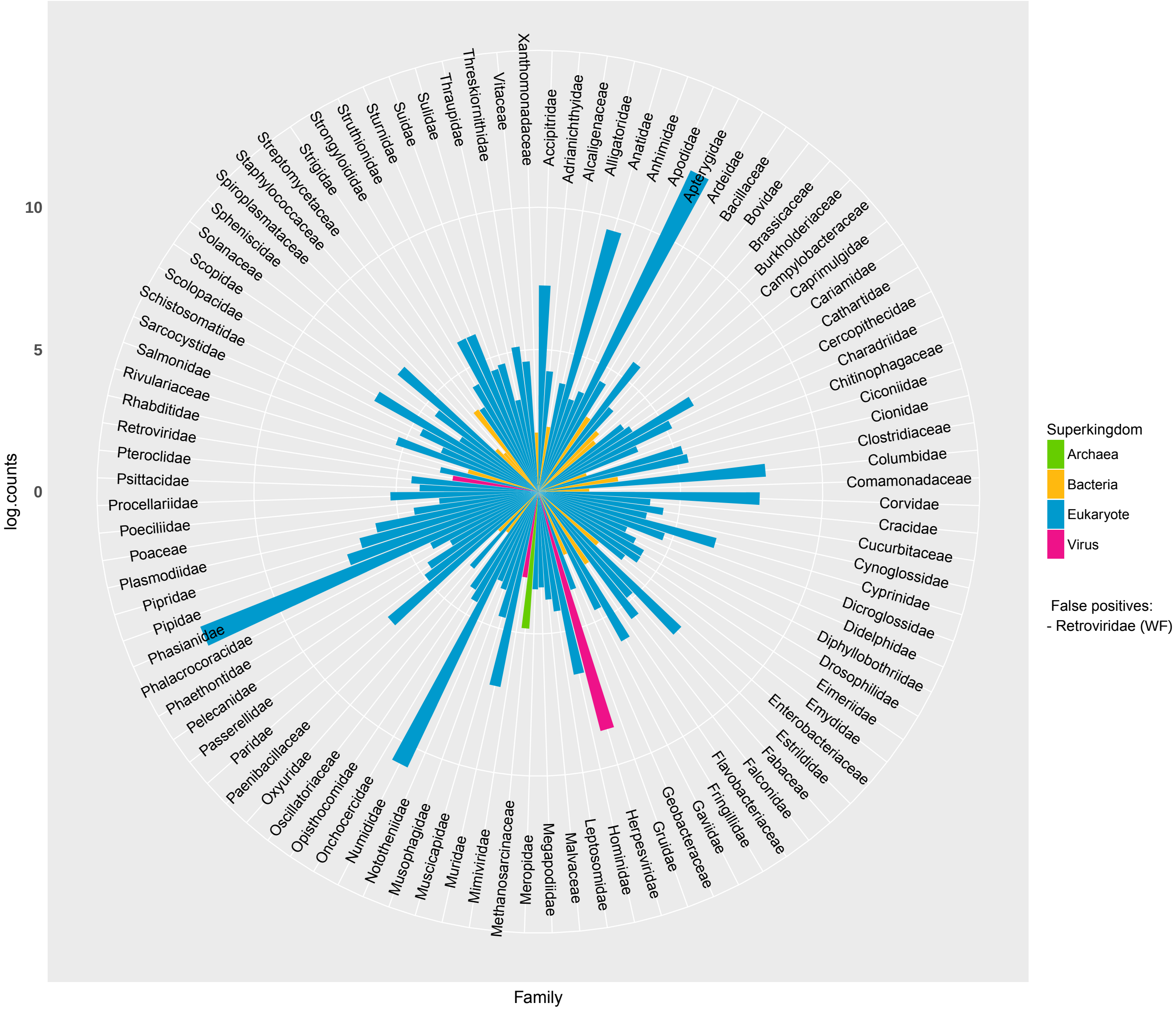

Figure 3: Graphical representation of read counts for the 100 most abundant families. In order to represent all detected superkingdoms, the displayed families are selected from the complete result applying the HighestAverages algorithm from the R package SciencesPo. Families are sorted alphabetically, color-coded according to superkingdoms. Note that the graph is in log-scale.

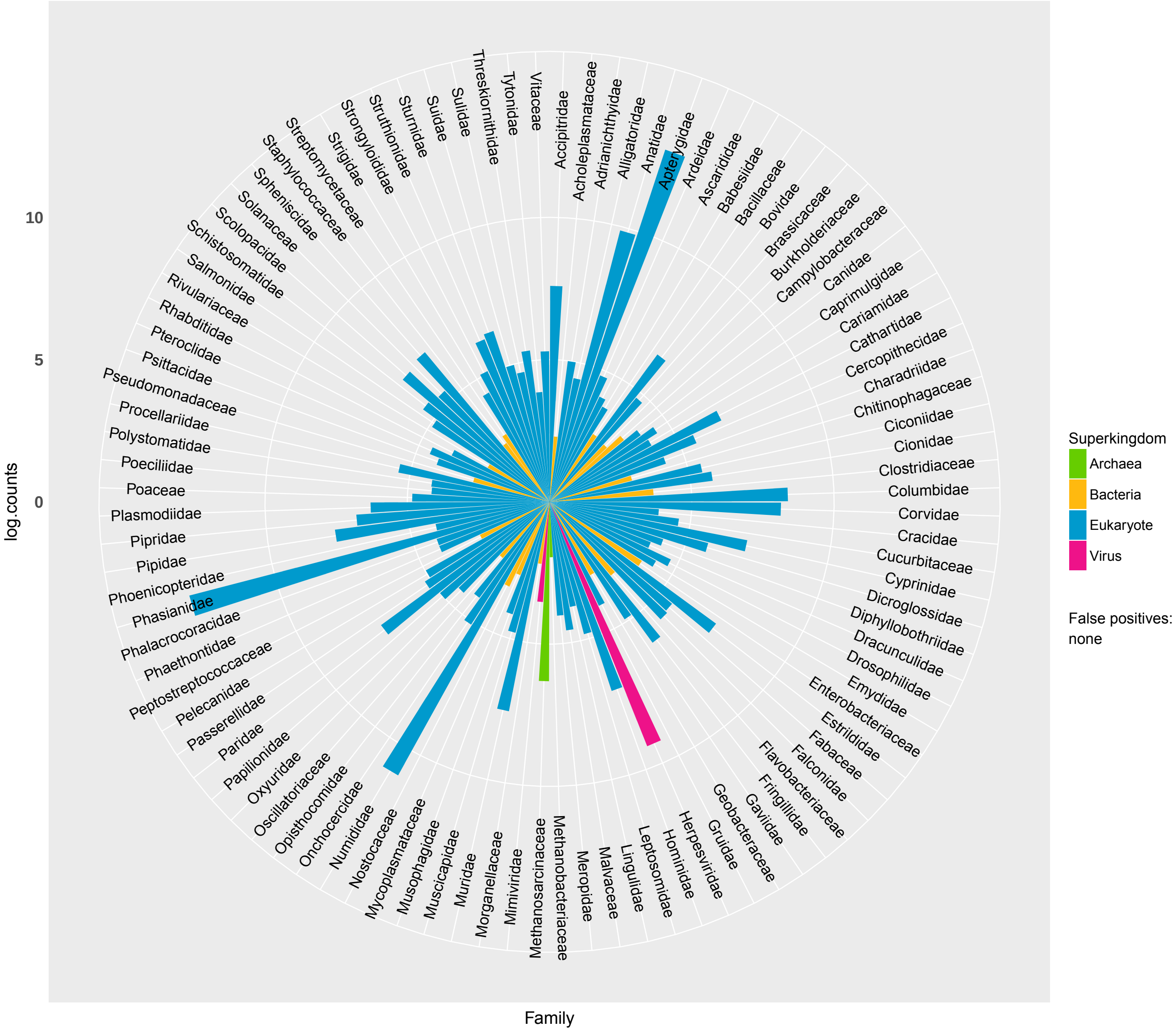

Figure 3: Graphical representation of read counts for the 100 most abundant families. In order to represent all detected superkingdoms, the displayed families are selected from the complete result applying the HighestAverages algorithm from the R package SciencesPo. Families are sorted alphabetically, color-coded according to superkingdoms. Note that the graph is in log-scale.

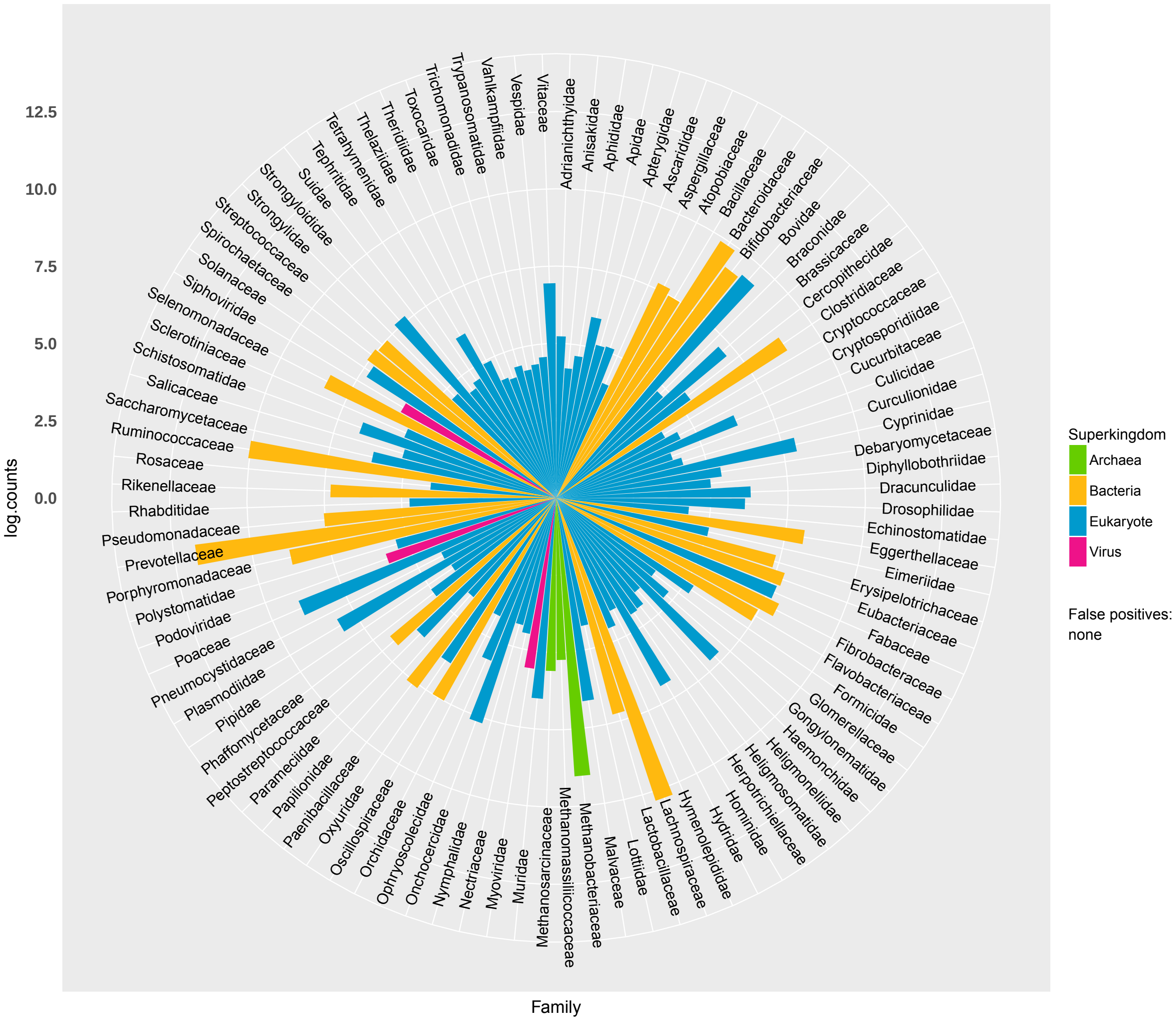

Figure 3: Graphical representation of read counts for the 100 most abundant families. In order to represent all detected superkingdoms, the displayed families are selected from the complete result applying the HighestAverages algorithm from the R package SciencesPo. Families are sorted alphabetically, color-coded according to superkingdoms. Note that the graph is in log-scale.

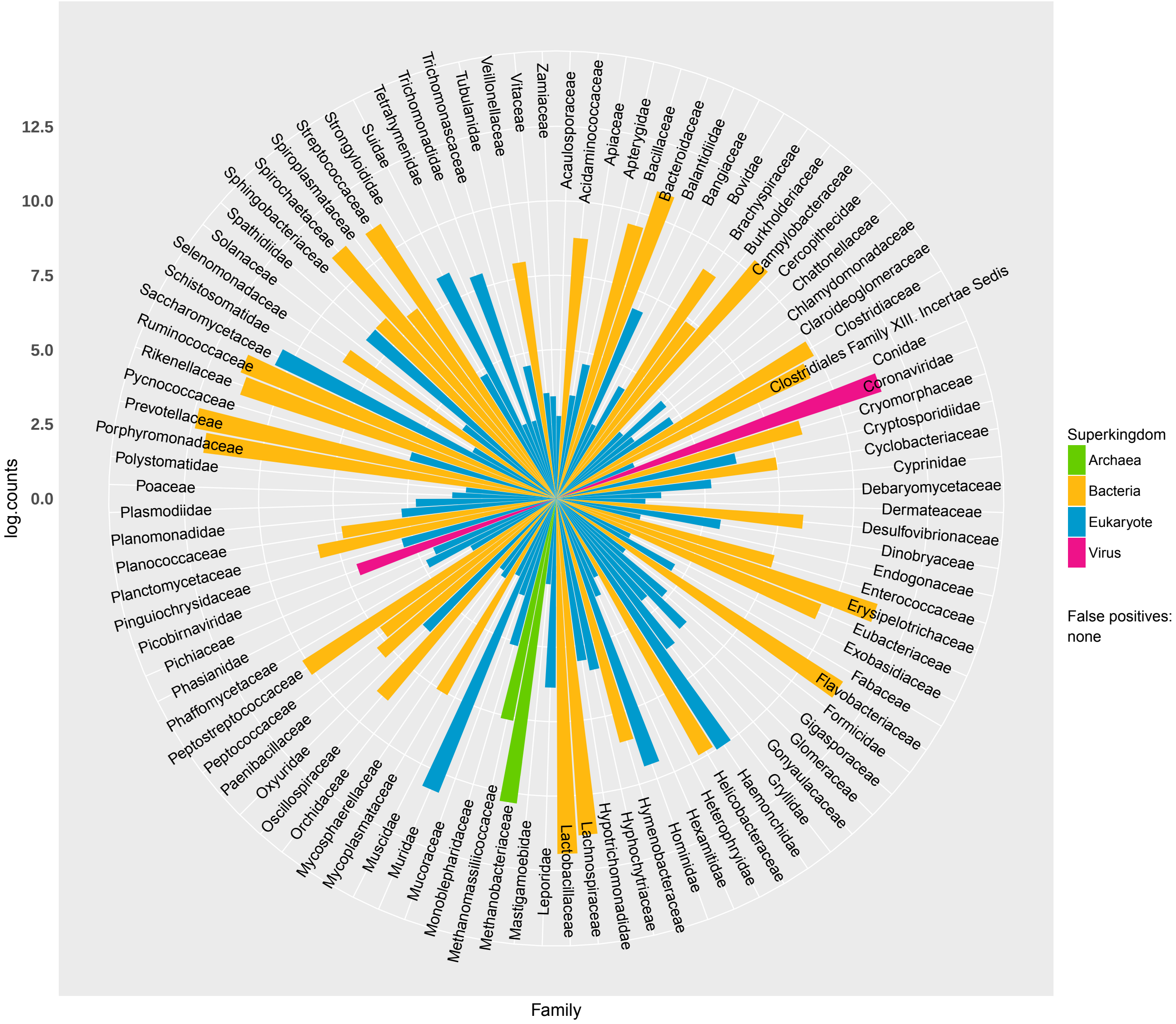

Figure 3: Graphical representation of read counts for the 100 most abundant families. In order to represent all detected superkingdoms, the displayed families are selected from the complete result applying the HighestAverages algorithm from the R package SciencesPo. Families are sorted alphabetically, color-coded according to superkingdoms. Note that the graph is in log-scale.

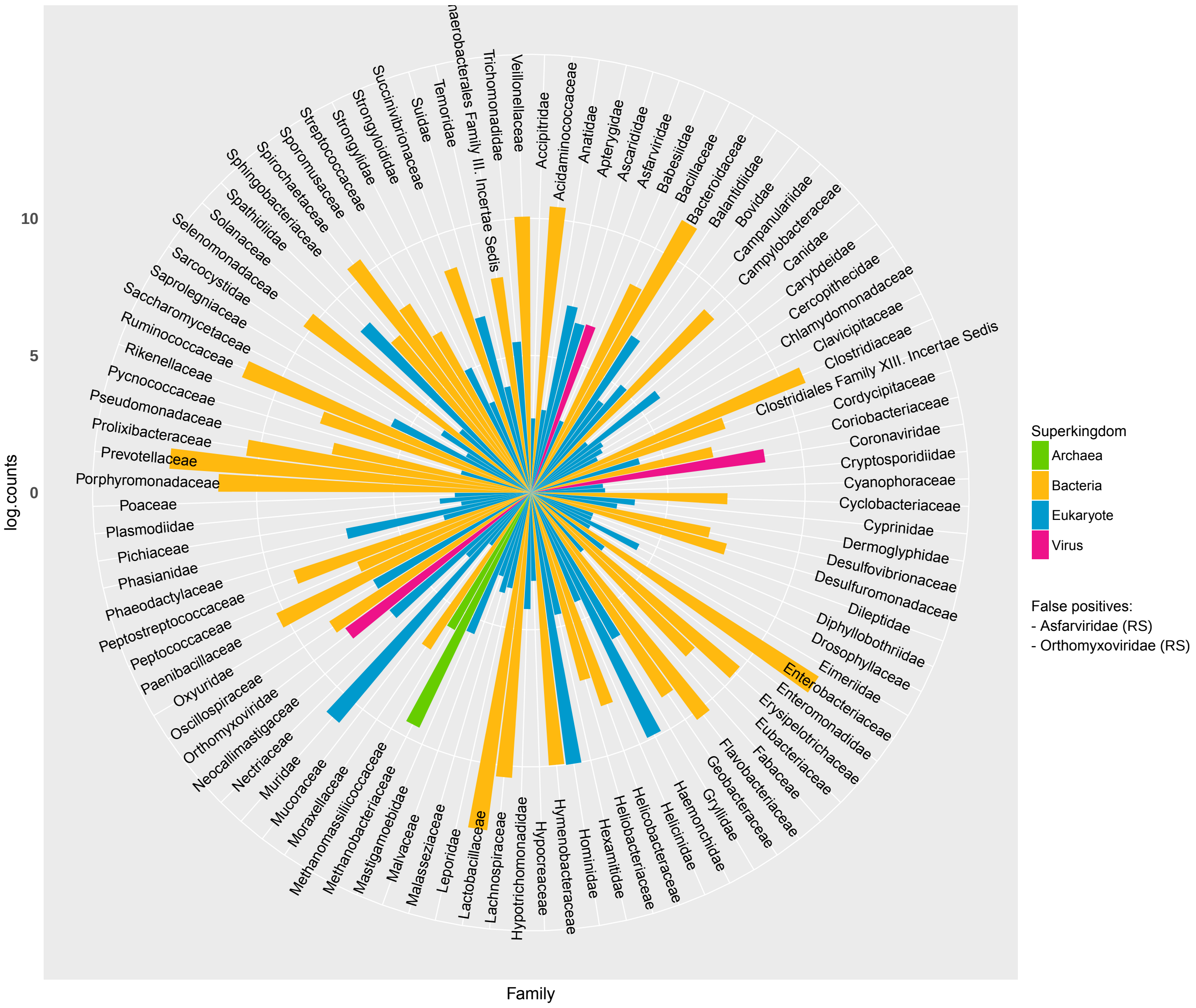

Figure 3: Graphical representation of read counts for the 100 most abundant families. In order to represent all detected superkingdoms, the displayed families are selected from the complete result applying the HighestAverages algorithm from the R package SciencesPo. Families are sorted alphabetically, color-coded according to superkingdoms. Note that the graph is in log-scale.

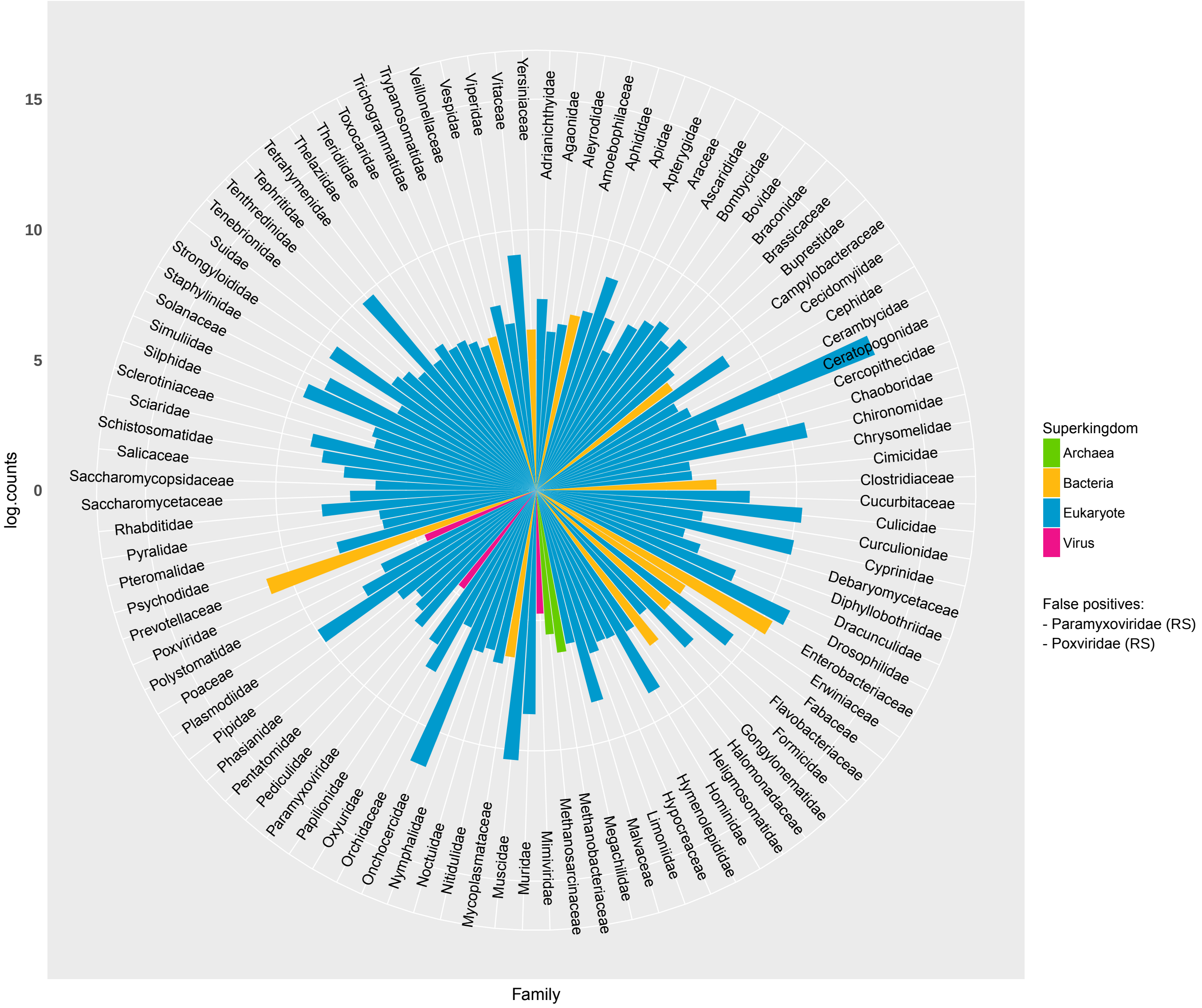

Figure 3: Graphical representation of read counts for the 100 most abundant families. In order to represent all detected superkingdoms, the displayed families are selected from the complete result applying the HighestAverages algorithm from the R package SciencesPo. Families are sorted alphabetically, color-coded according to superkingdoms. Note that the graph is in log-scale.

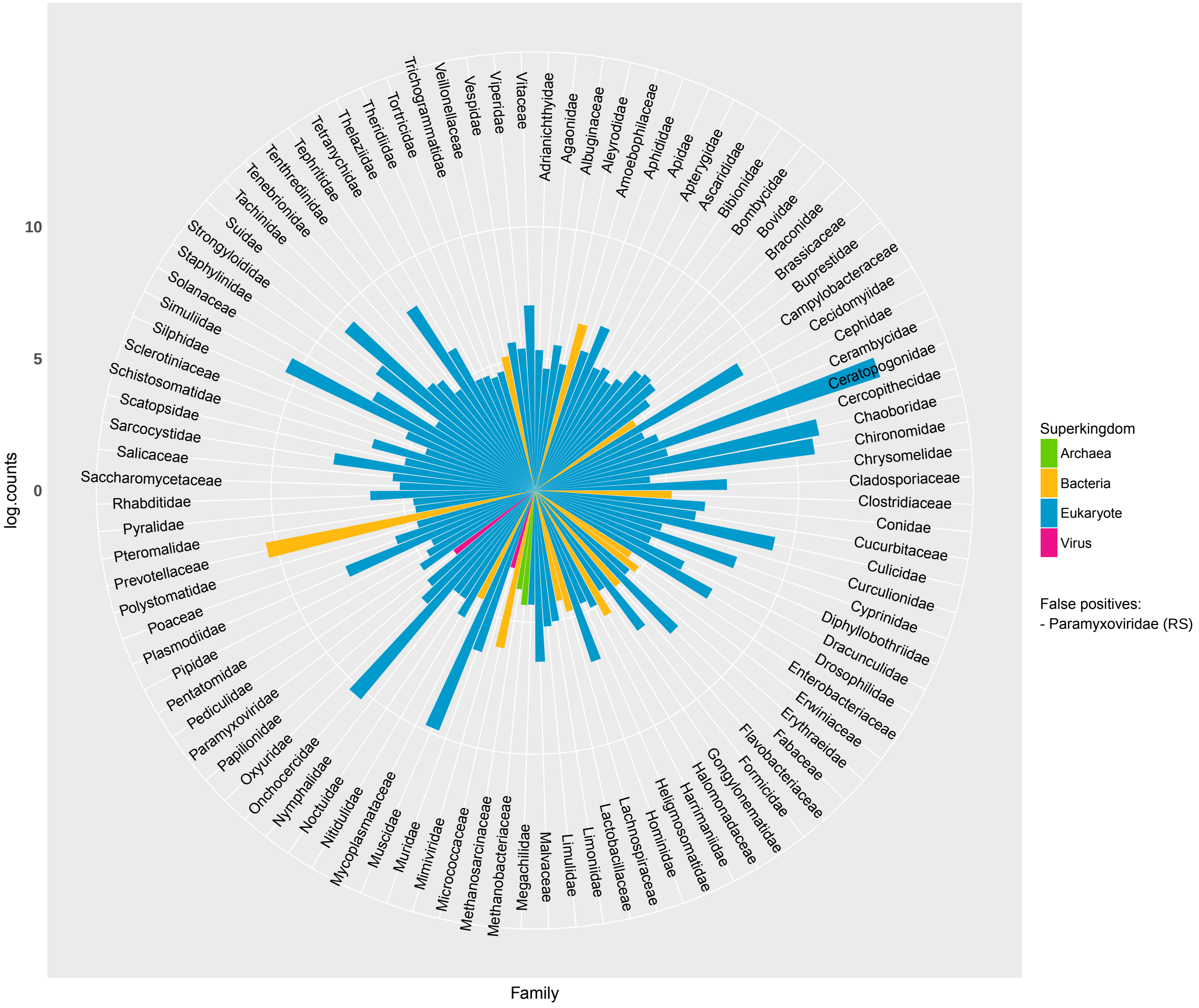

Figure 3: Graphical representation of read counts for the 100 most abundant families. In order to represent all detected superkingdoms, the displayed families are selected from the complete result applying the HighestAverages algorithm from the R package SciencesPo. Families are sorted alphabetically, color-coded according to superkingdoms. Note that the graph is in log-scale.

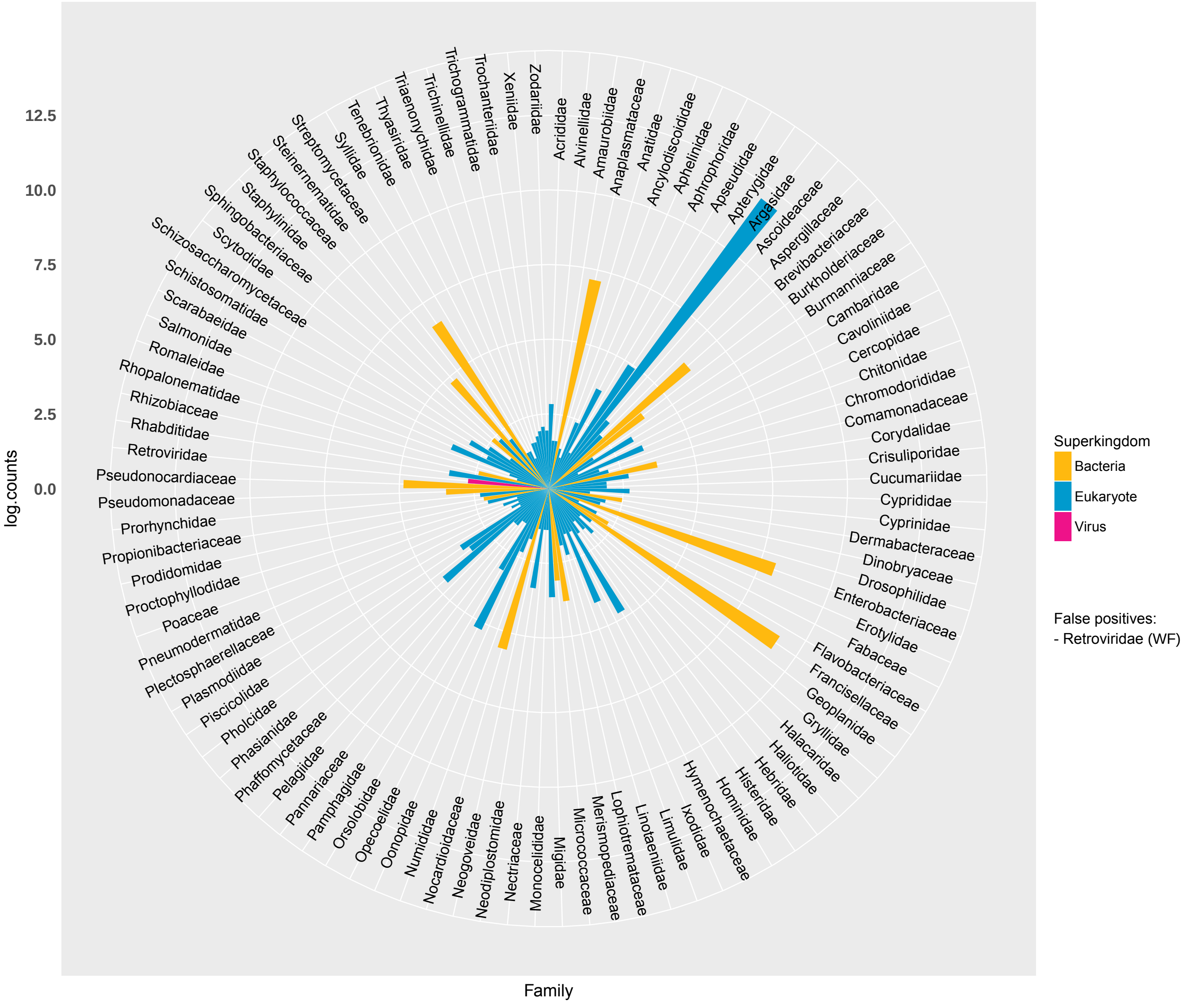

Figure 3: Graphical representation of read counts for the 100 most abundant families. In order to represent all detected superkingdoms, the displayed families are selected from the complete result applying the HighestAverages algorithm from the R package SciencesPo. Families are sorted alphabetically, color-coded according to superkingdoms. Note that the graph is in log-scale.

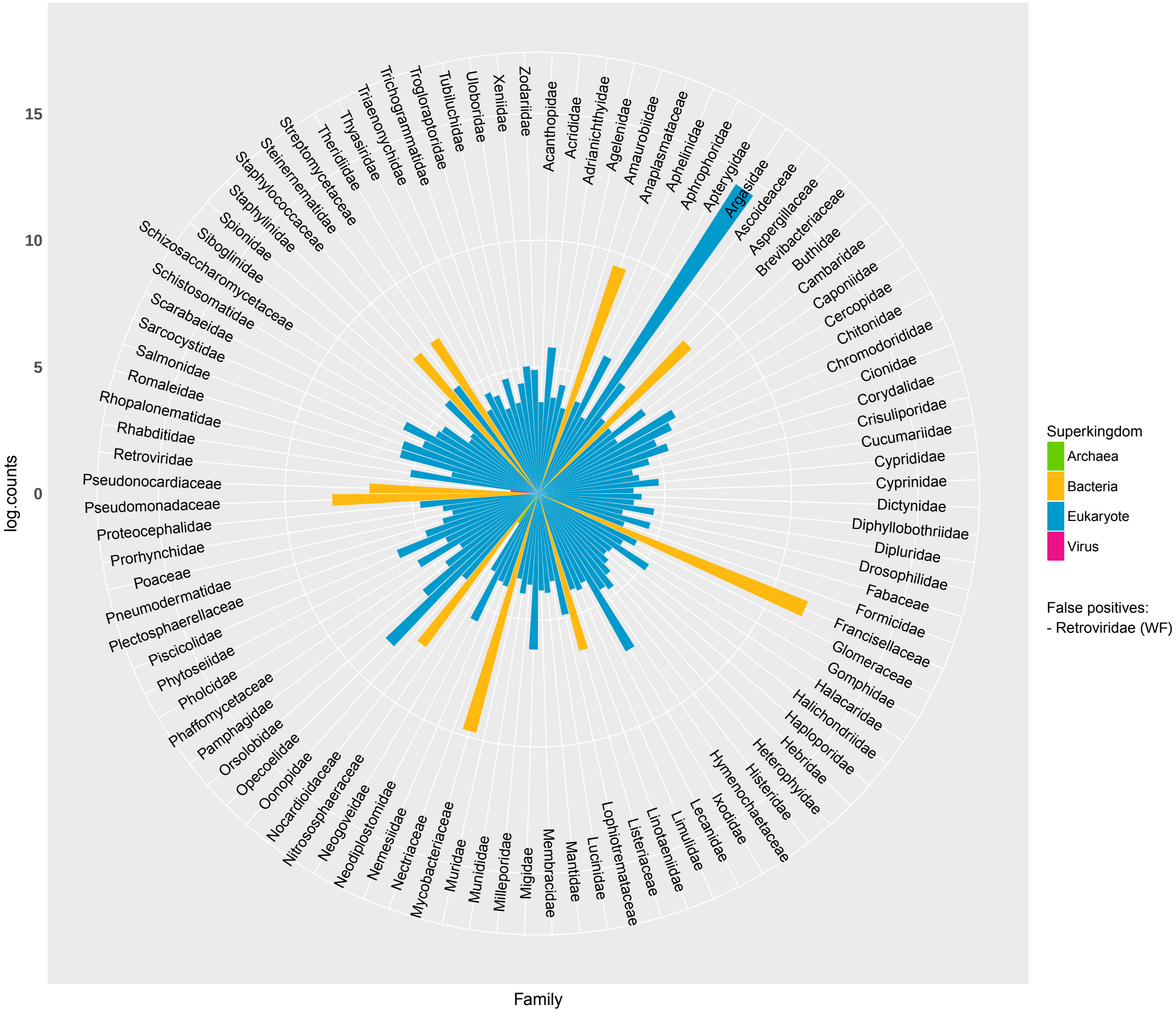

Figure 3: Graphical representation of read counts for the 100 most abundant families. In order to represent all detected superkingdoms, the displayed families are selected from the complete result applying the HighestAverages algorithm from the R package SciencesPo. Families are sorted alphabetically, color-coded according to superkingdoms. Note that the graph is in log-scale.

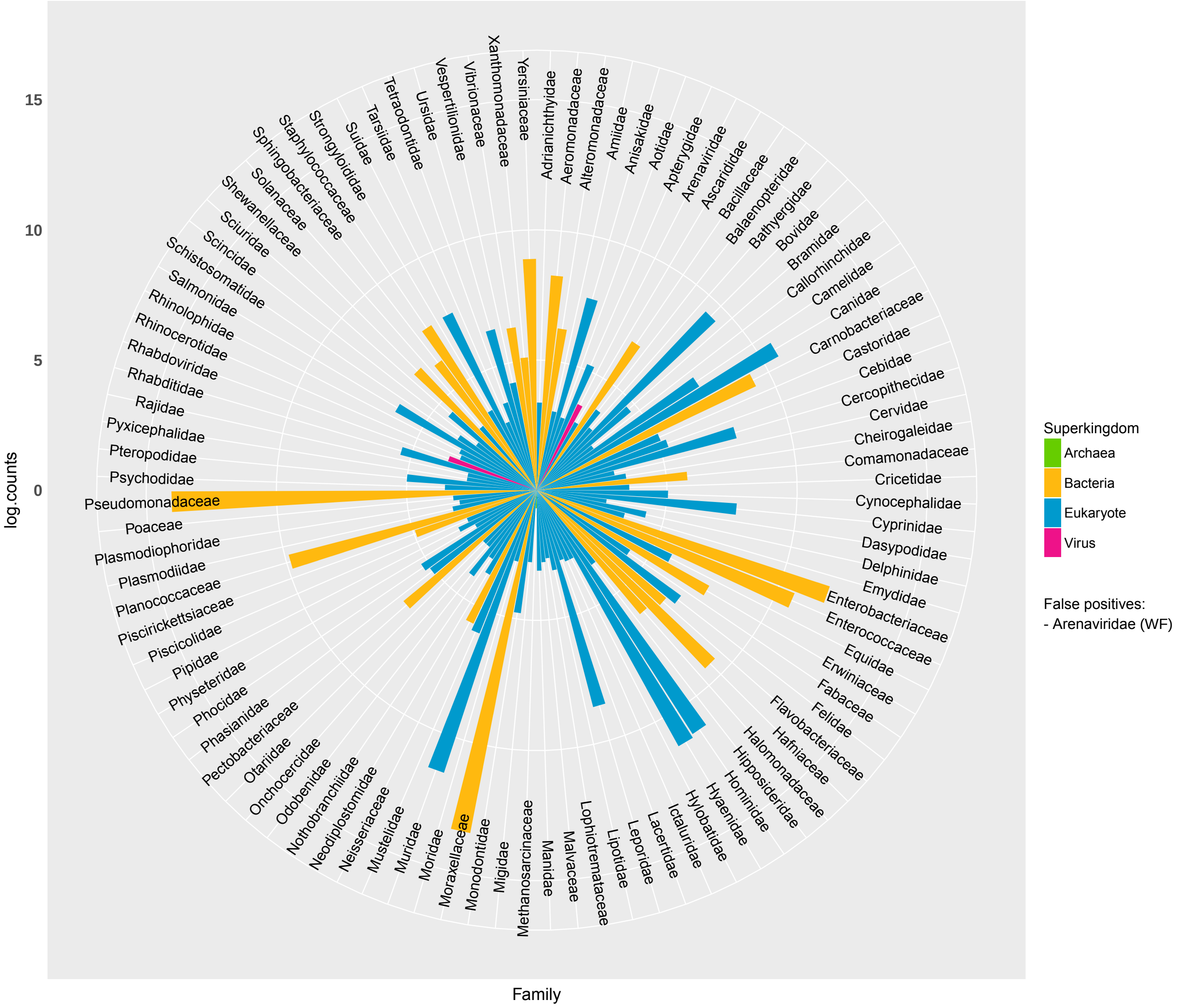

Figure 3: Graphical representation of read counts for the 100 most abundant families. In order to represent all detected superkingdoms, the displayed families are selected from the complete result applying the HighestAverages algorithm from the R package SciencesPo. Families are sorted alphabetically, color-coded according to superkingdoms. Note that the graph is in log-scale.

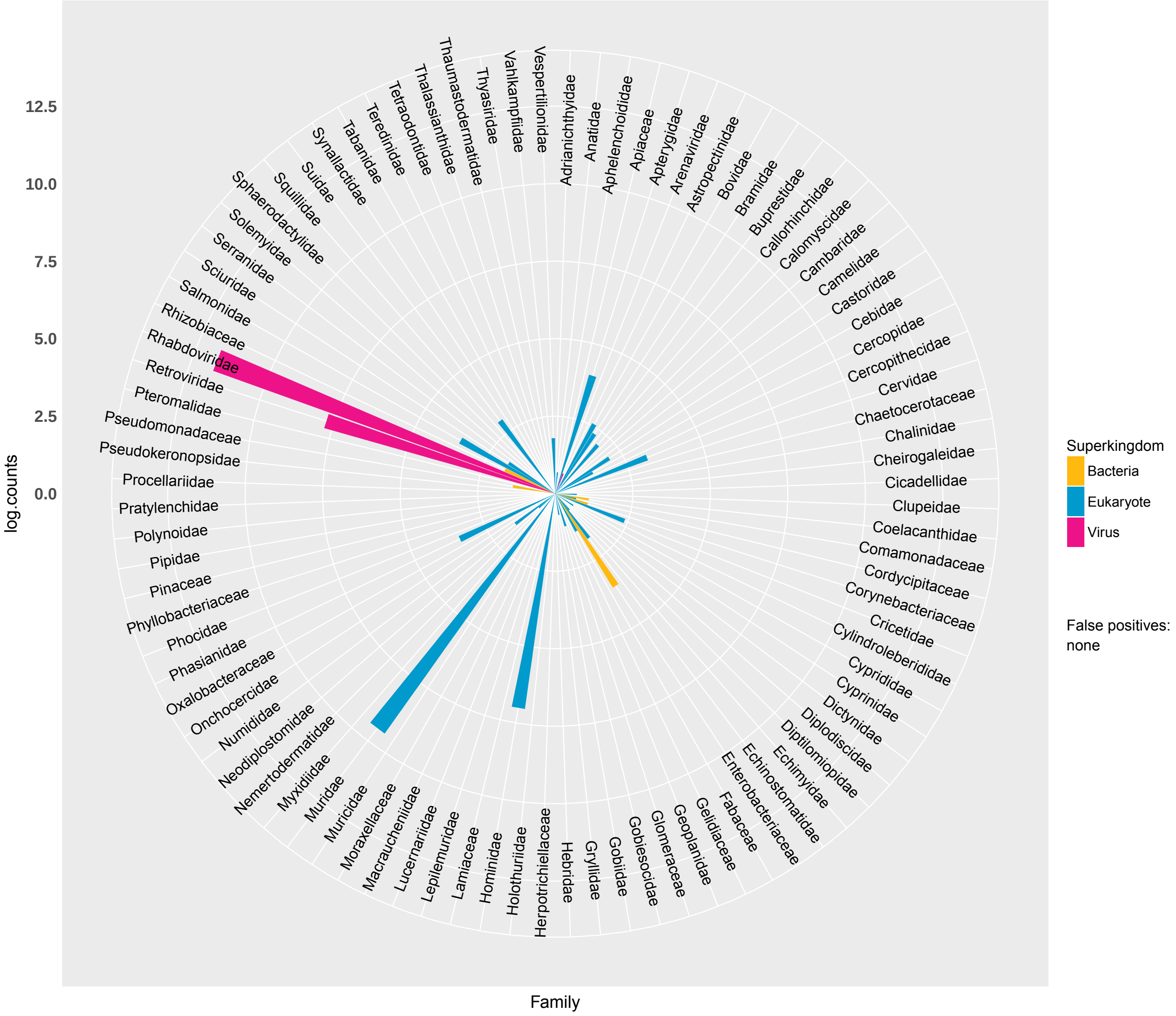

Figure 3: Graphical representation of read counts for the 100 most abundant families. In order to represent all detected superkingdoms, the displayed families are selected from the complete result applying the HighestAverages algorithm from the R package SciencesPo. Families are sorted alphabetically, color-coded according to superkingdoms. Note that the graph is in log-scale.

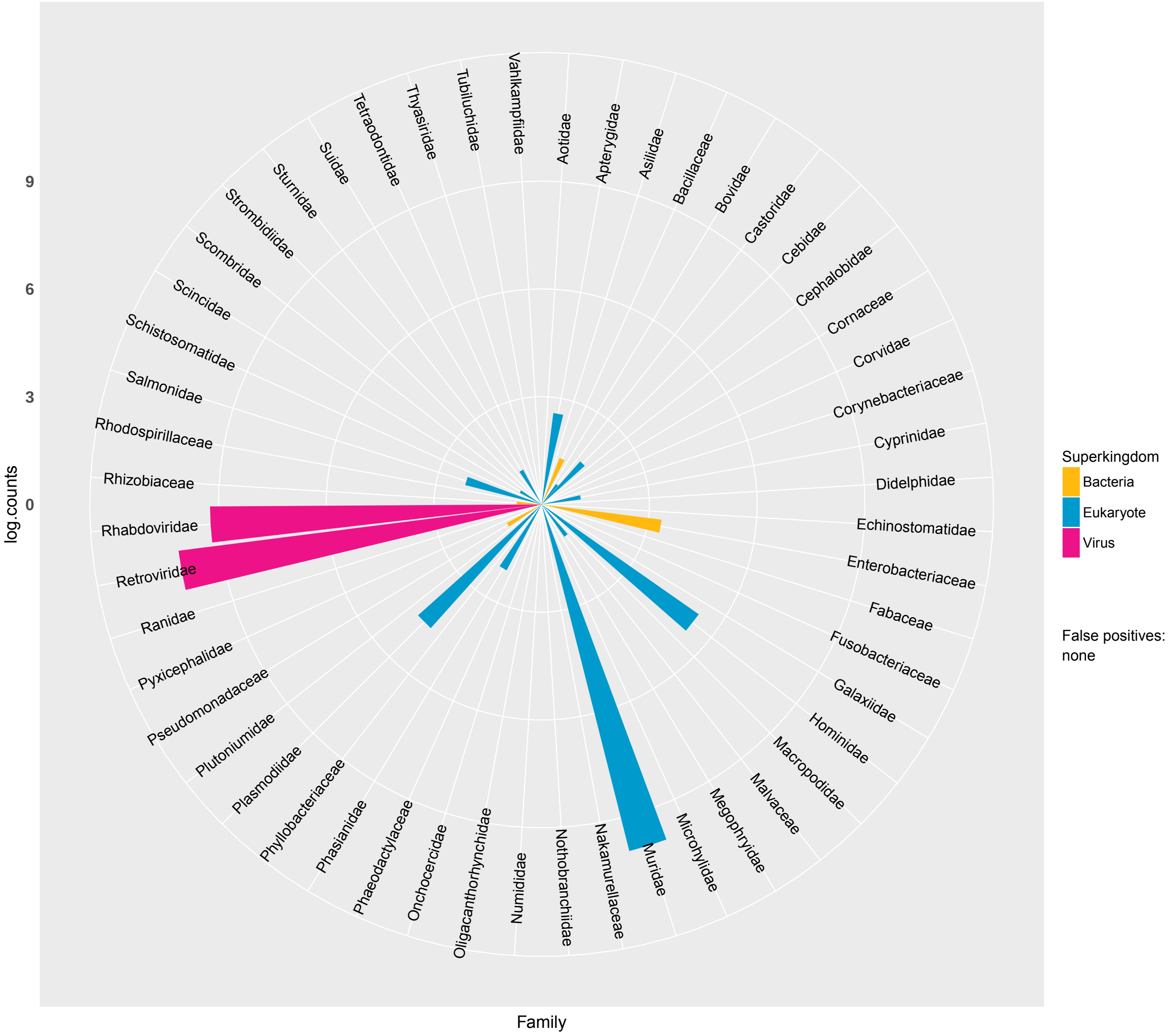

Figure 3: Graphical representation of read counts for the 100 most abundant families. In order to represent all detected superkingdoms, the displayed families are selected from the complete result applying the HighestAverages algorithm from the R package SciencesPo. Families are sorted alphabetically, color-coded according to superkingdoms. Note that the graph is in log-scale.

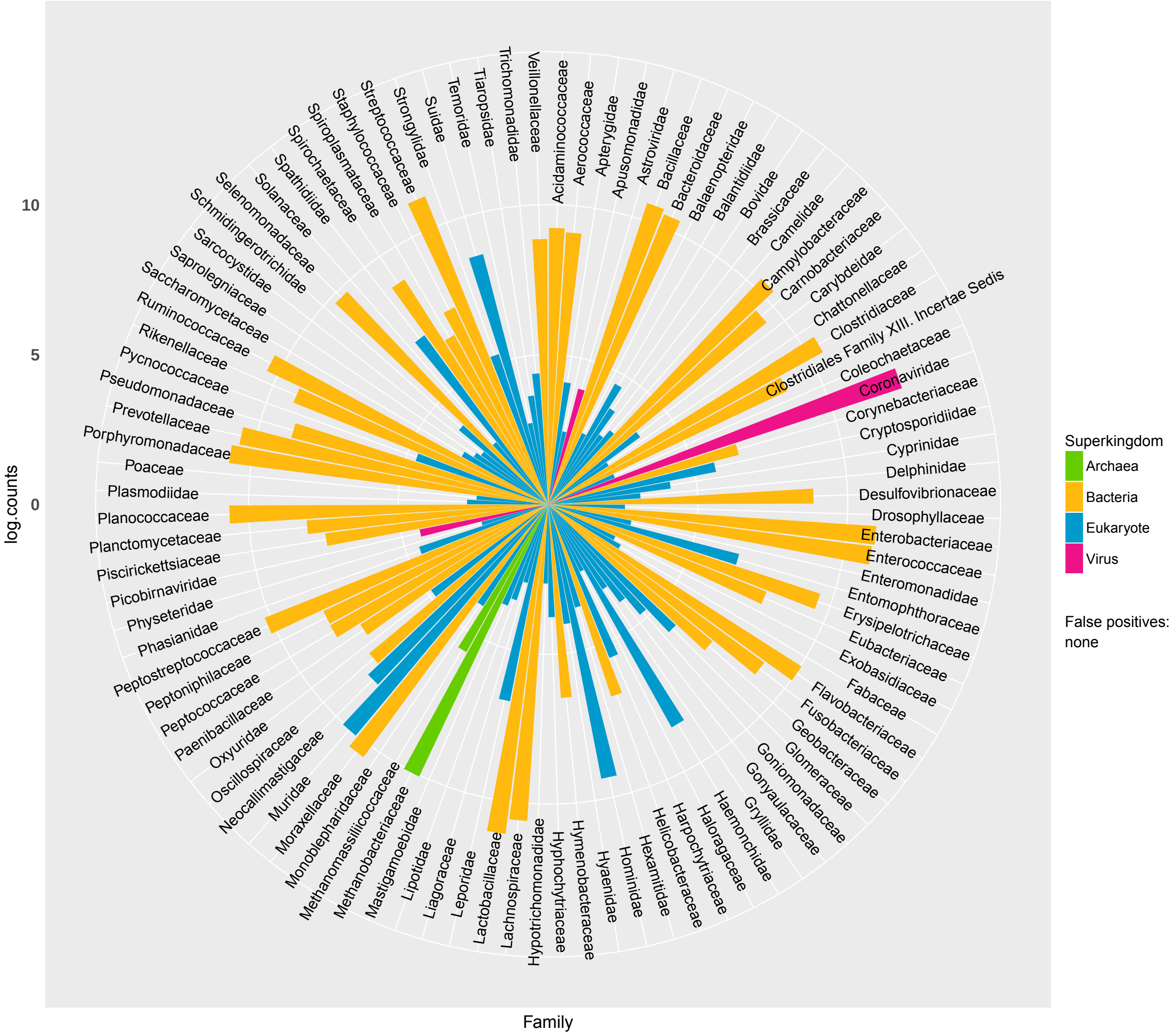

Figure 3: Graphical representation of read counts for the 100 most abundant families. In order to represent all detected superkingdoms, the displayed families are selected from the complete result applying the HighestAverages algorithm from the R package SciencesPo. Families are sorted alphabetically, color-coded according to superkingdoms. Note that the graph is in log-scale.

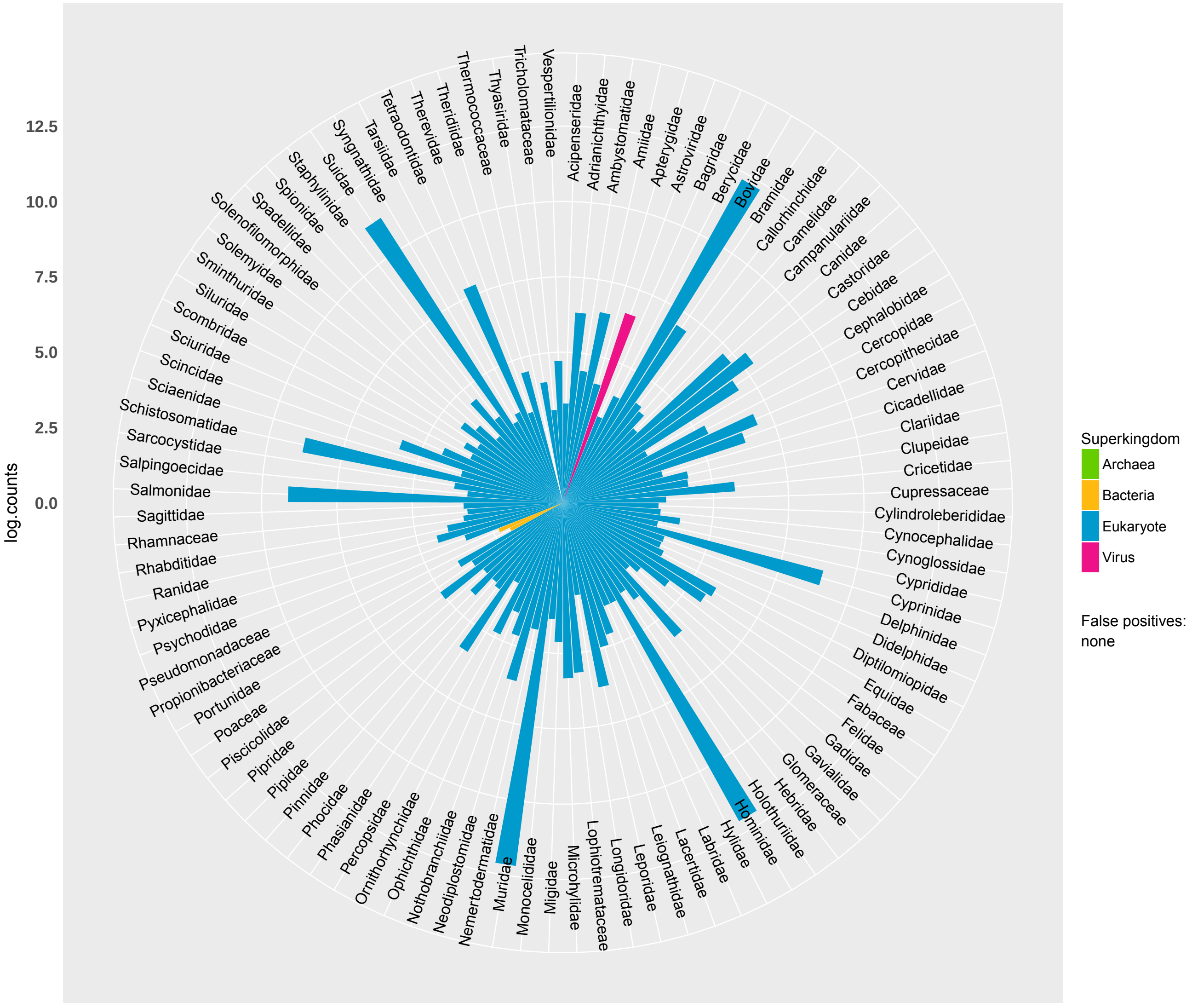

Figure 3: Graphical representation of read counts for the 100 most abundant families. In order to represent all detected superkingdoms, the displayed families are selected from the complete result applying the HighestAverages algorithm from the R package SciencesPo. Families are sorted alphabetically, color-coded according to superkingdoms. Note that the graph is in log-scale.

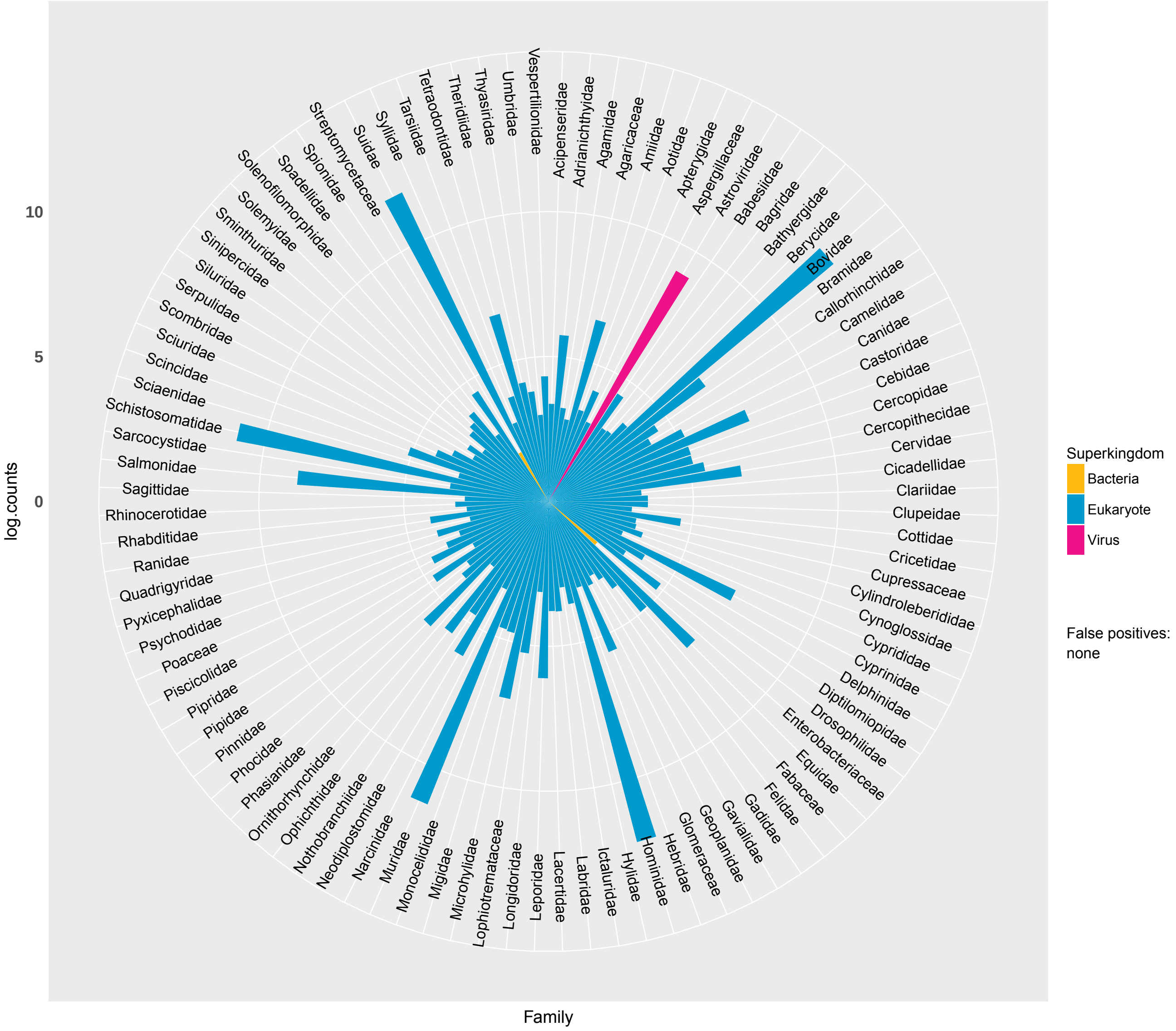

Figure 3: Graphical representation of read counts for the 100 most abundant families. In order to represent all detected superkingdoms, the displayed families are selected from the complete result applying the HighestAverages algorithm from the R package SciencesPo. Families are sorted alphabetically, color-coded according to superkingdoms. Note that the graph is in log-scale.

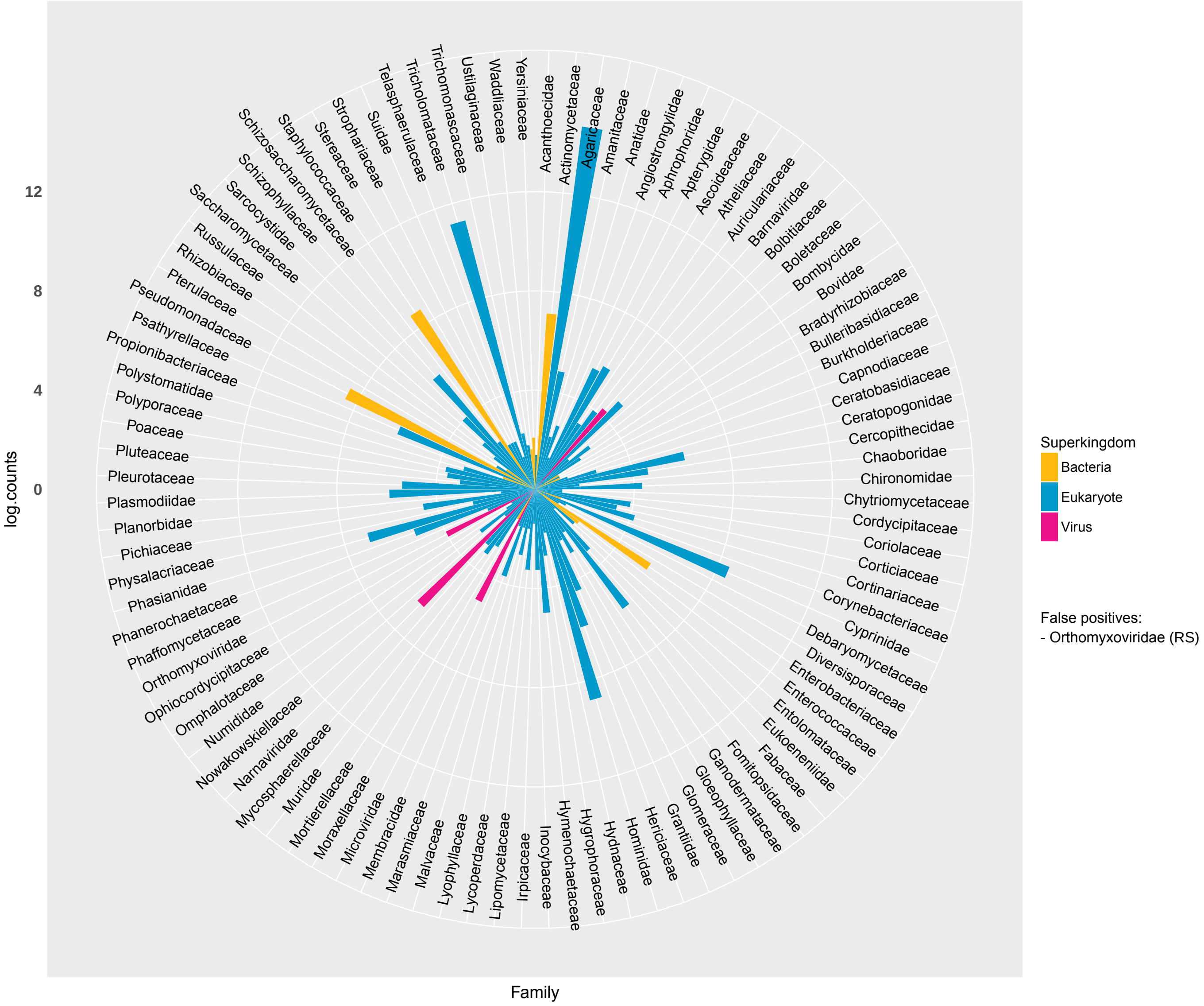

Figure 3: Graphical representation of read counts for the 100 most abundant families. In order to represent all detected superkingdoms, the displayed families are selected from the complete result applying the HighestAverages algorithm from the R package SciencesPo. Families are sorted alphabetically, color-coded according to superkingdoms. Note that the graph is in log-scale.

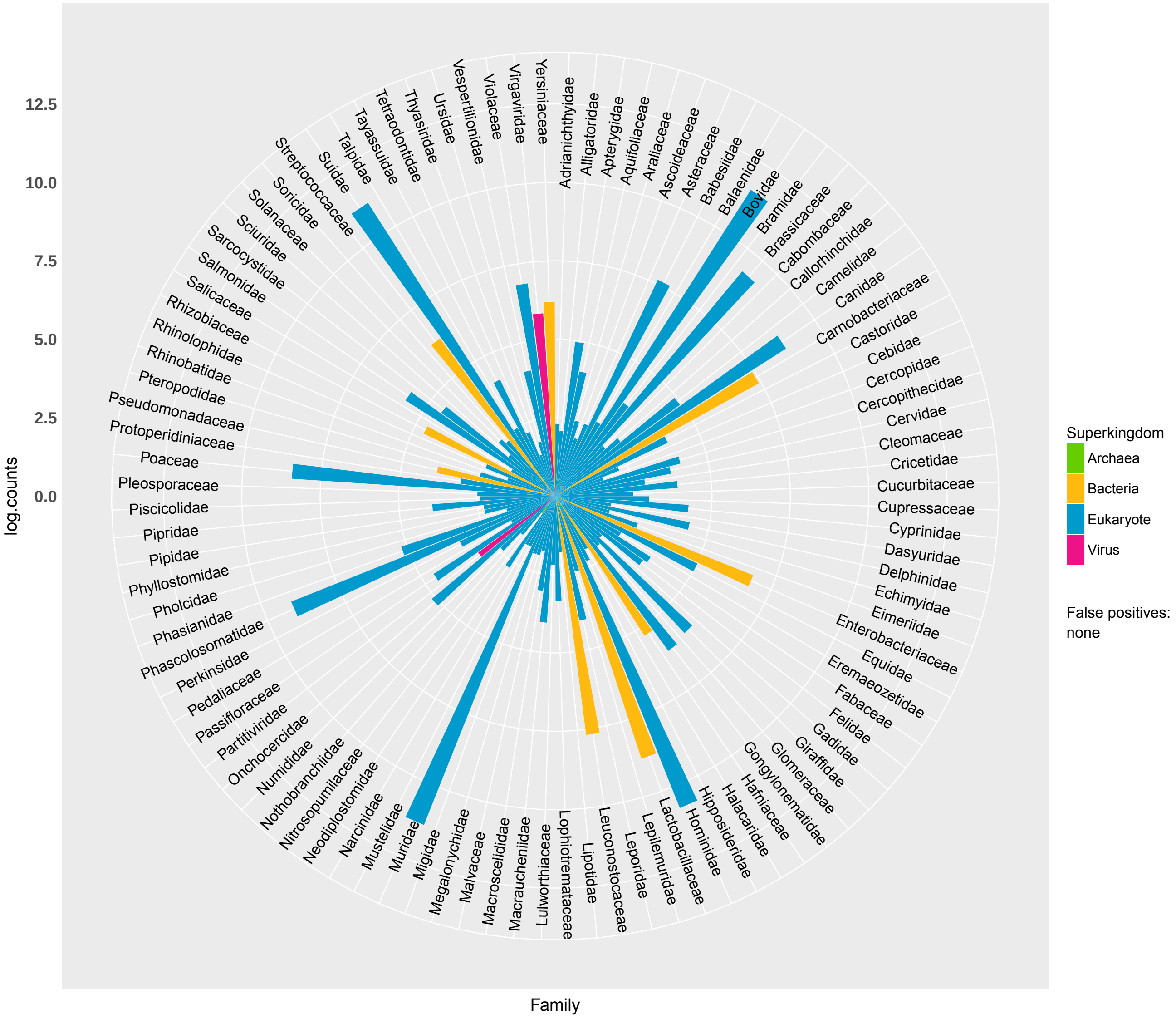

Figure 3: Graphical representation of read counts for the 100 most abundant families. In order to represent all detected superkingdoms, the displayed families are selected from the complete result applying the HighestAverages algorithm from the R package SciencesPo. Families are sorted alphabetically, color-coded according to superkingdoms. Note that the graph is in log-scale.

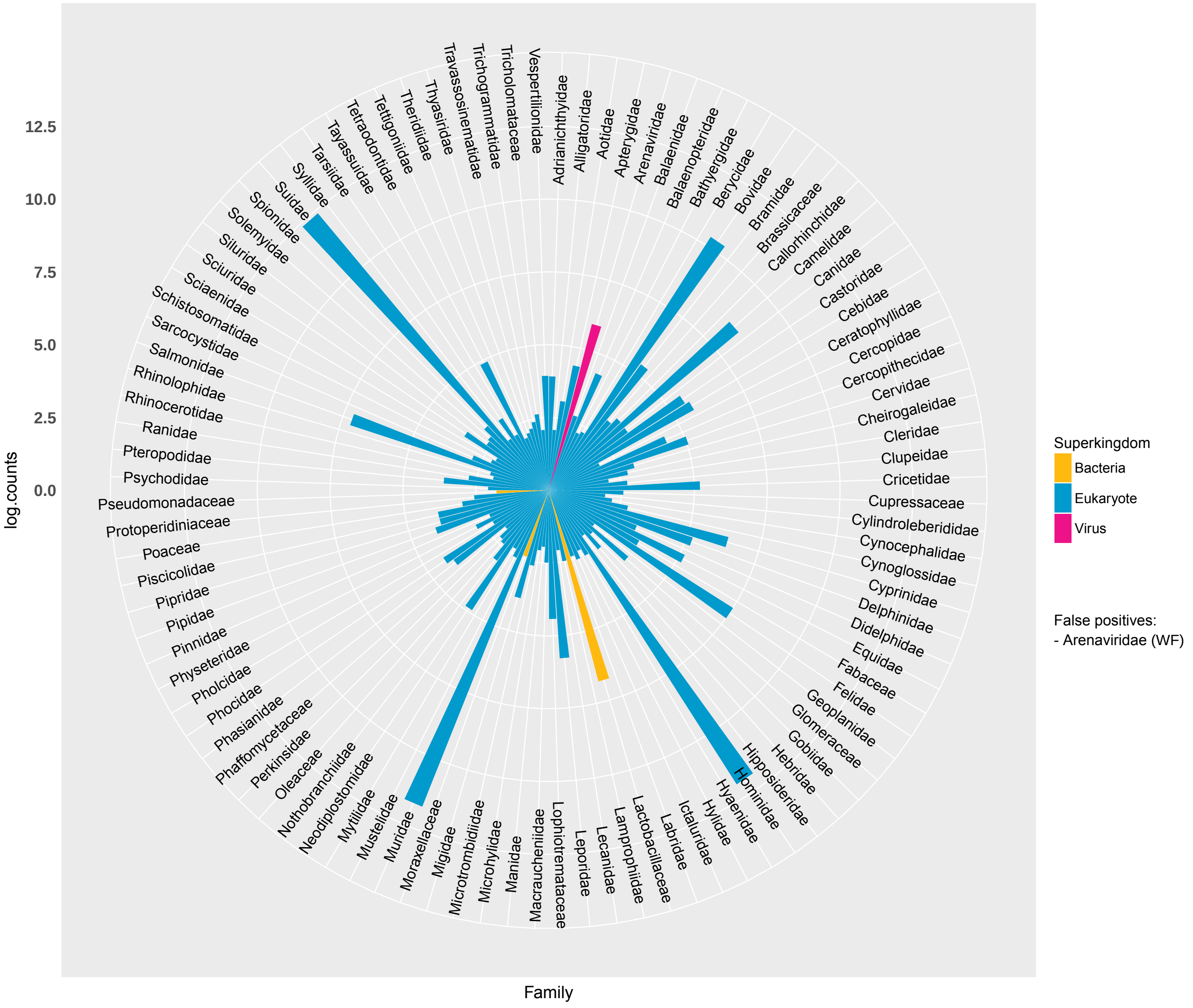

Figure 3: Graphical representation of read counts for the 100 most abundant families. In order to represent all detected superkingdoms, the displayed families are selected from the complete result applying the HighestAverages algorithm from the R package SciencesPo. Families are sorted alphabetically, color-coded according to superkingdoms. Note that the graph is in log-scale.

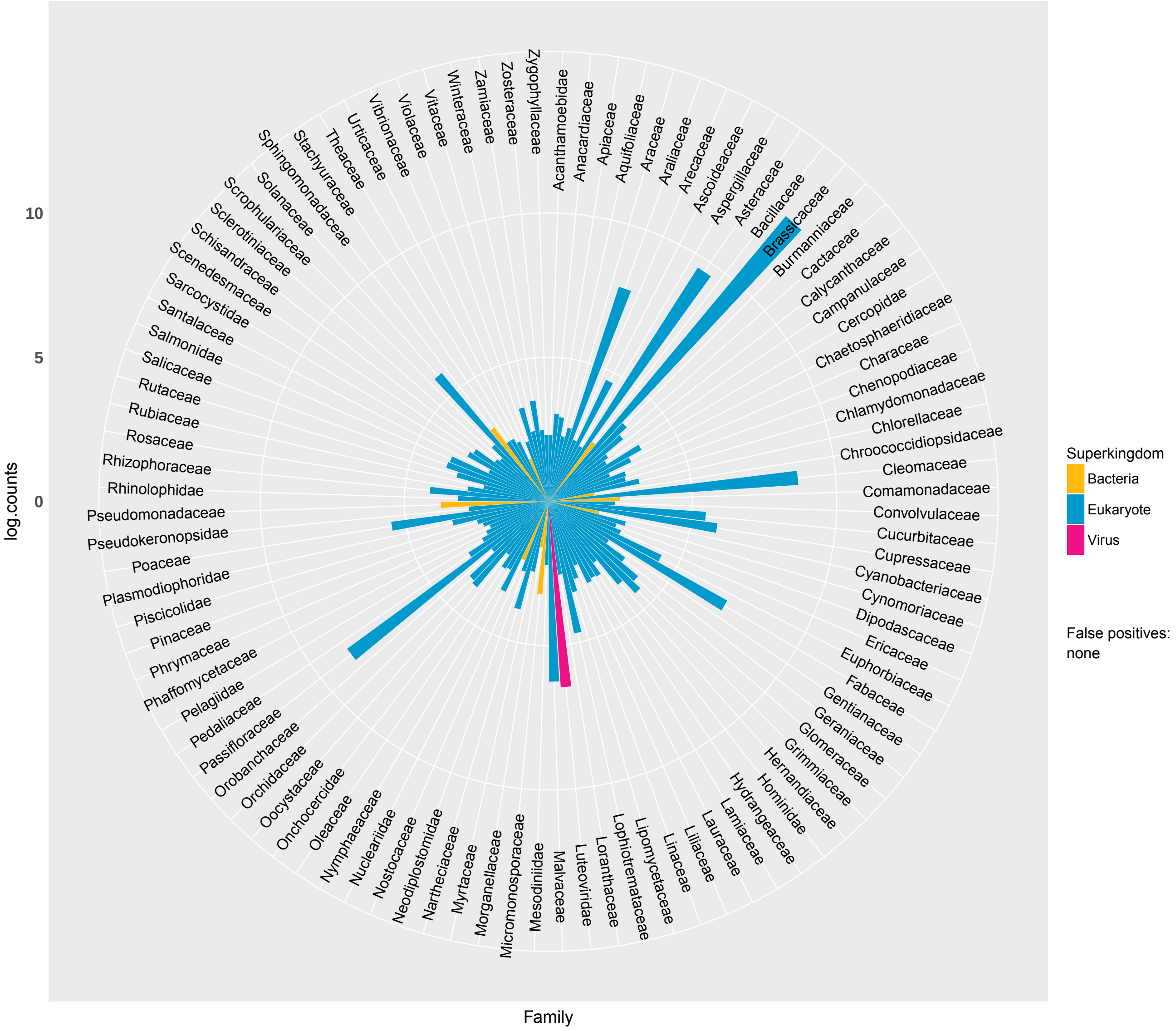

Figure 3: Graphical representation of read counts for the 100 most abundant families. In order to represent all detected superkingdoms, the displayed families are selected from the complete result applying the HighestAverages algorithm from the R package SciencesPo. Families are sorted alphabetically, color-coded according to superkingdoms. Note that the graph is in log-scale.

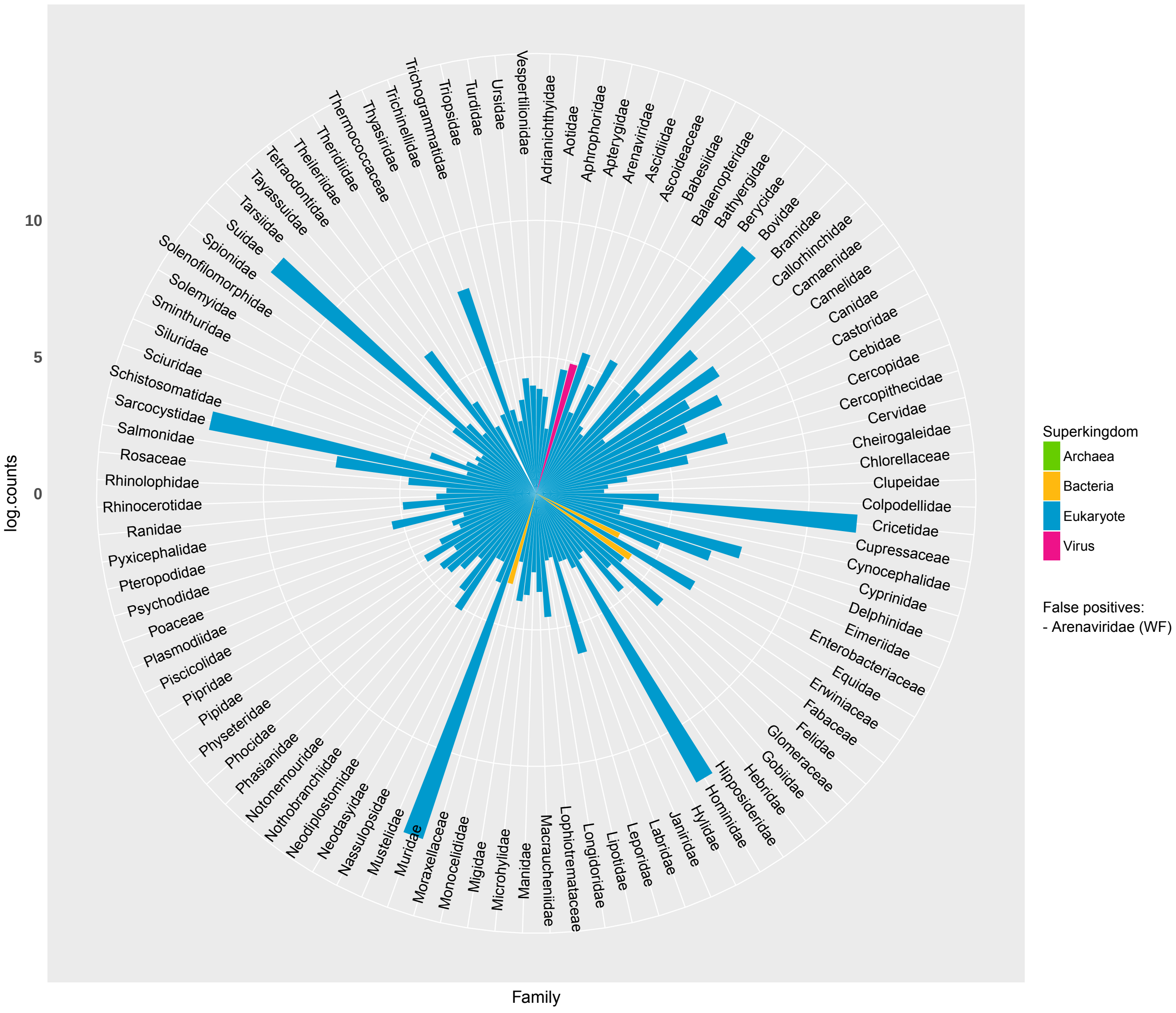

Figure 3: Graphical representation of read counts for the 100 most abundant families. In order to represent all detected superkingdoms, the displayed families are selected from the complete result applying the HighestAverages algorithm from the R package SciencesPo. Families are sorted alphabetically, color-coded according to superkingdoms. Note that the graph is in log-scale.

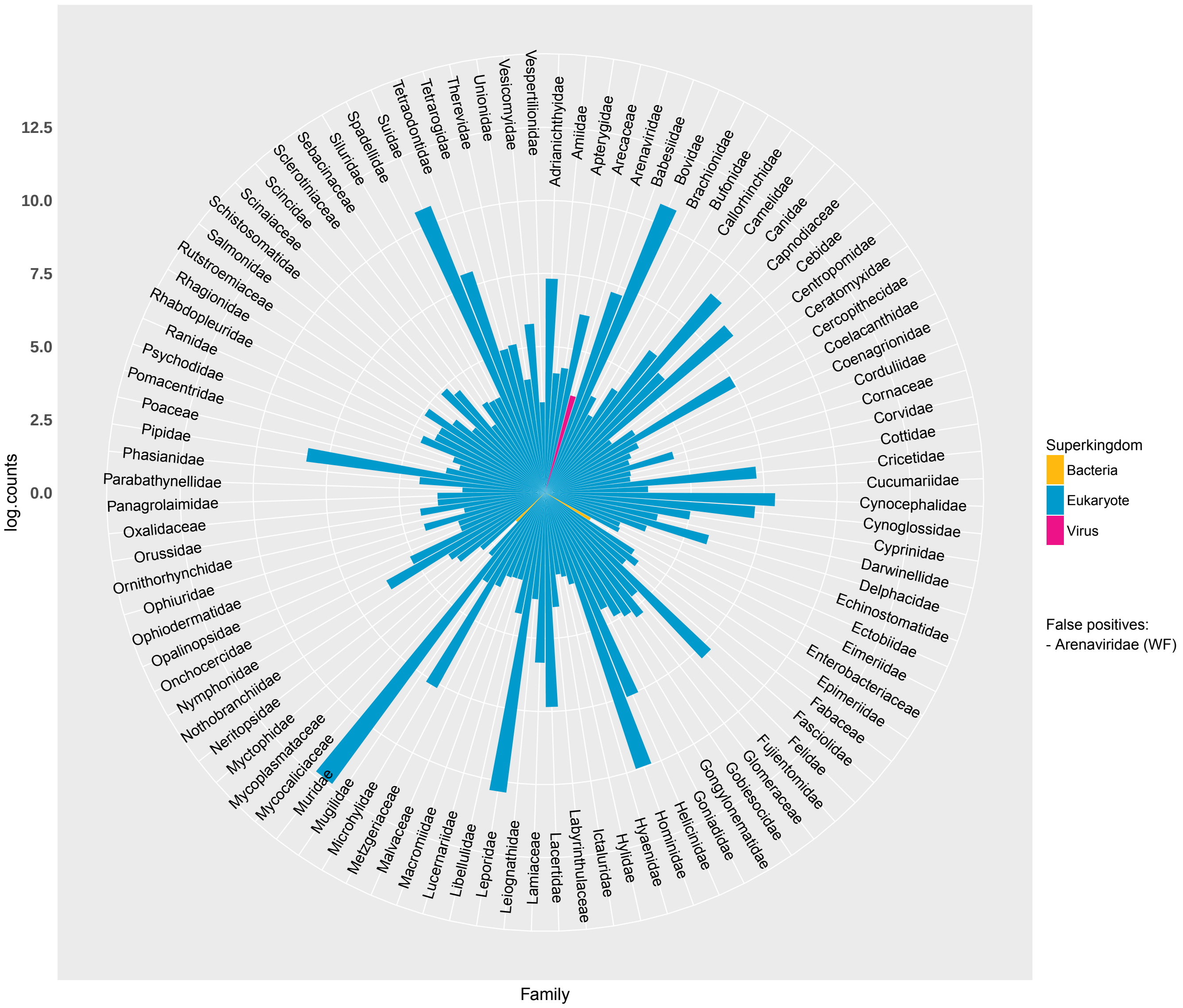

Figure 3: Graphical representation of read counts for the 100 most abundant families. In order to represent all detected superkingdoms, the displayed families are selected from the complete result applying the HighestAverages algorithm from the R package SciencesPo. Families are sorted alphabetically, color-coded according to superkingdoms. Note that the graph is in log-scale.

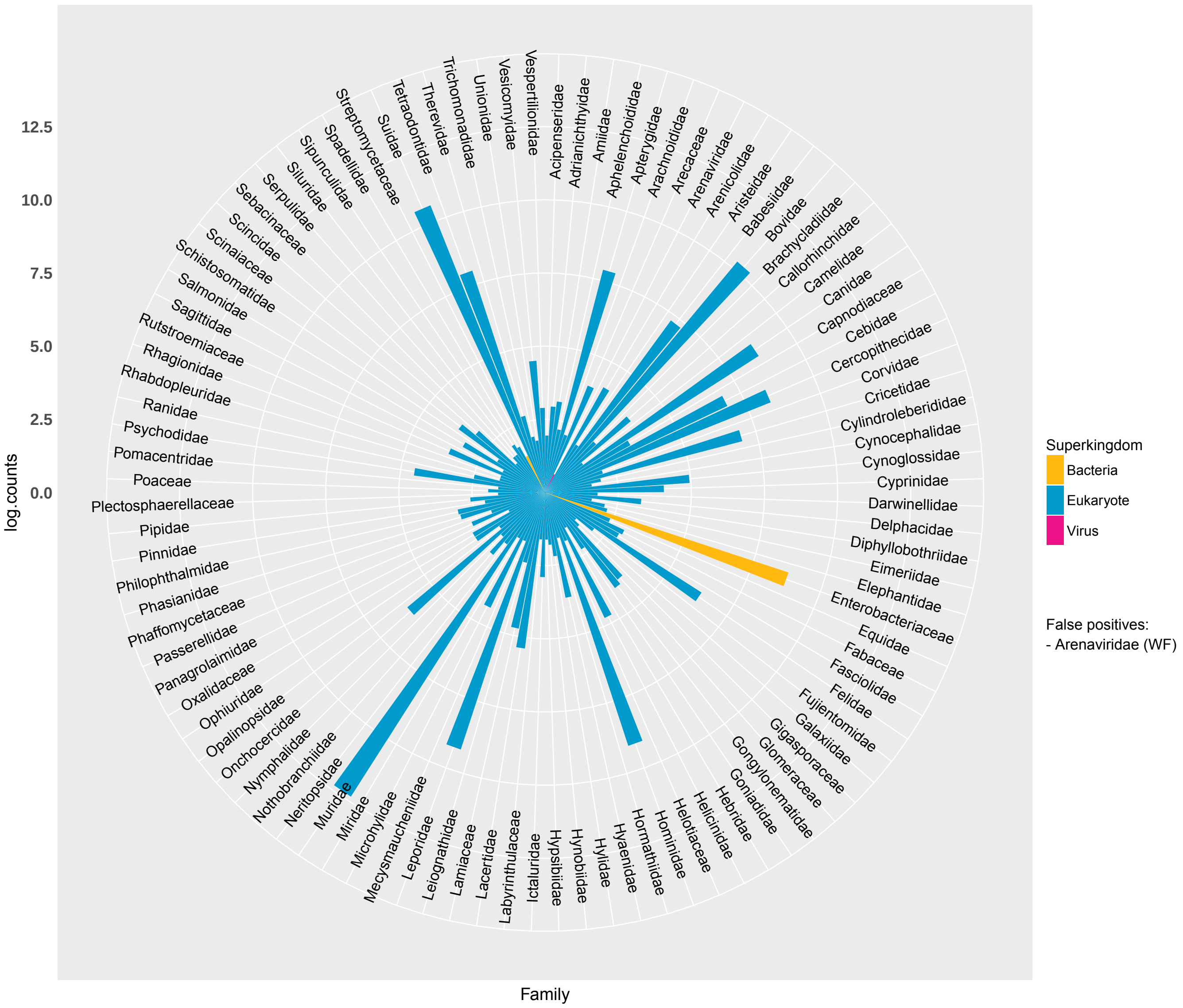

Figure 3: Graphical representation of read counts for the 100 most abundant families. In order to represent all detected superkingdoms, the displayed families are selected from the complete result applying the HighestAverages algorithm from the R package SciencesPo. Families are sorted alphabetically, color-coded according to superkingdoms. Note that the graph is in log-scale.

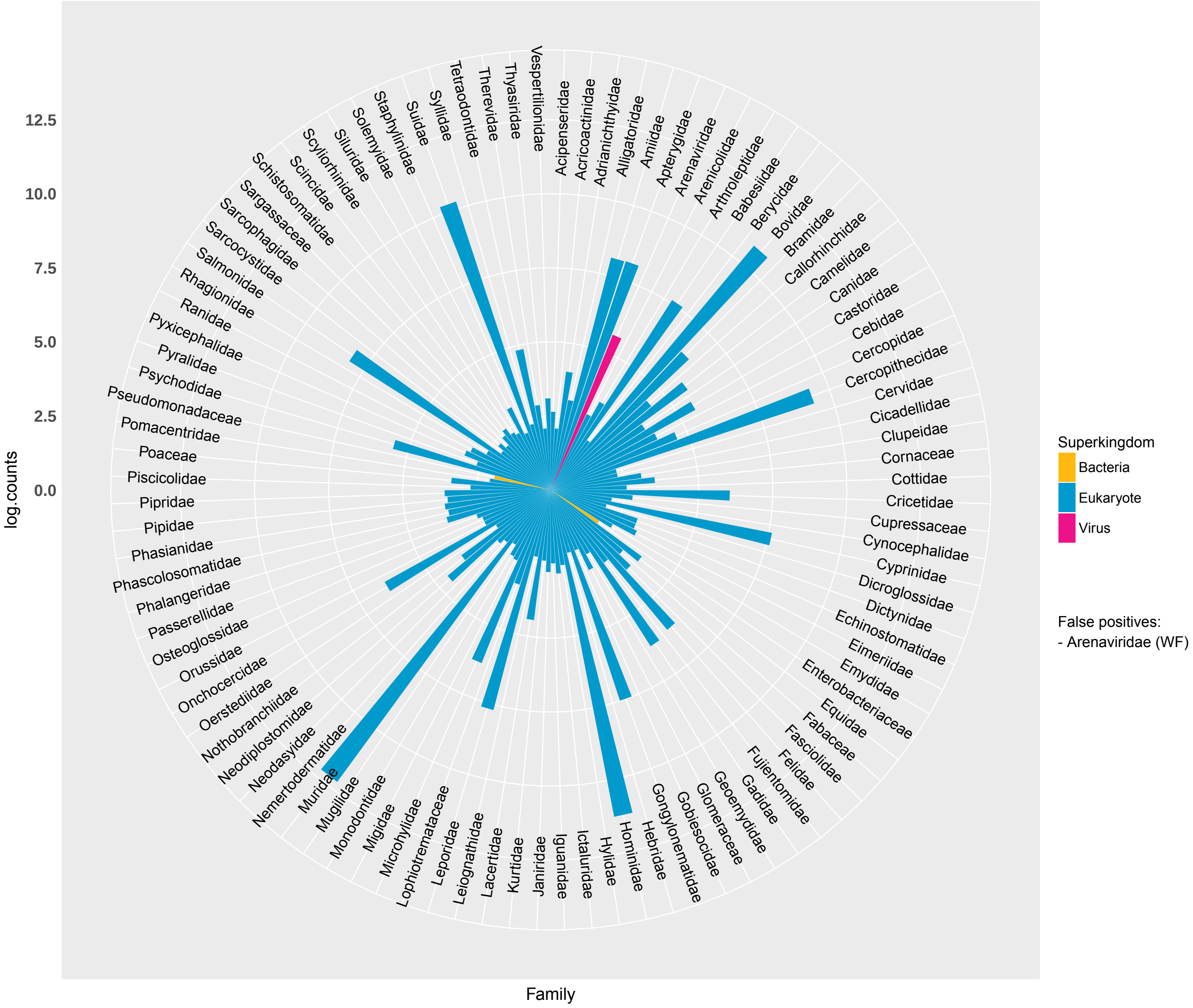

Figure 3: Graphical representation of read counts for the 100 most abundant families. In order to represent all detected superkingdoms, the displayed families are selected from the complete result applying the HighestAverages algorithm from the R package SciencesPo. Families are sorted alphabetically, color-coded according to superkingdoms. Note that the graph is in log-scale.

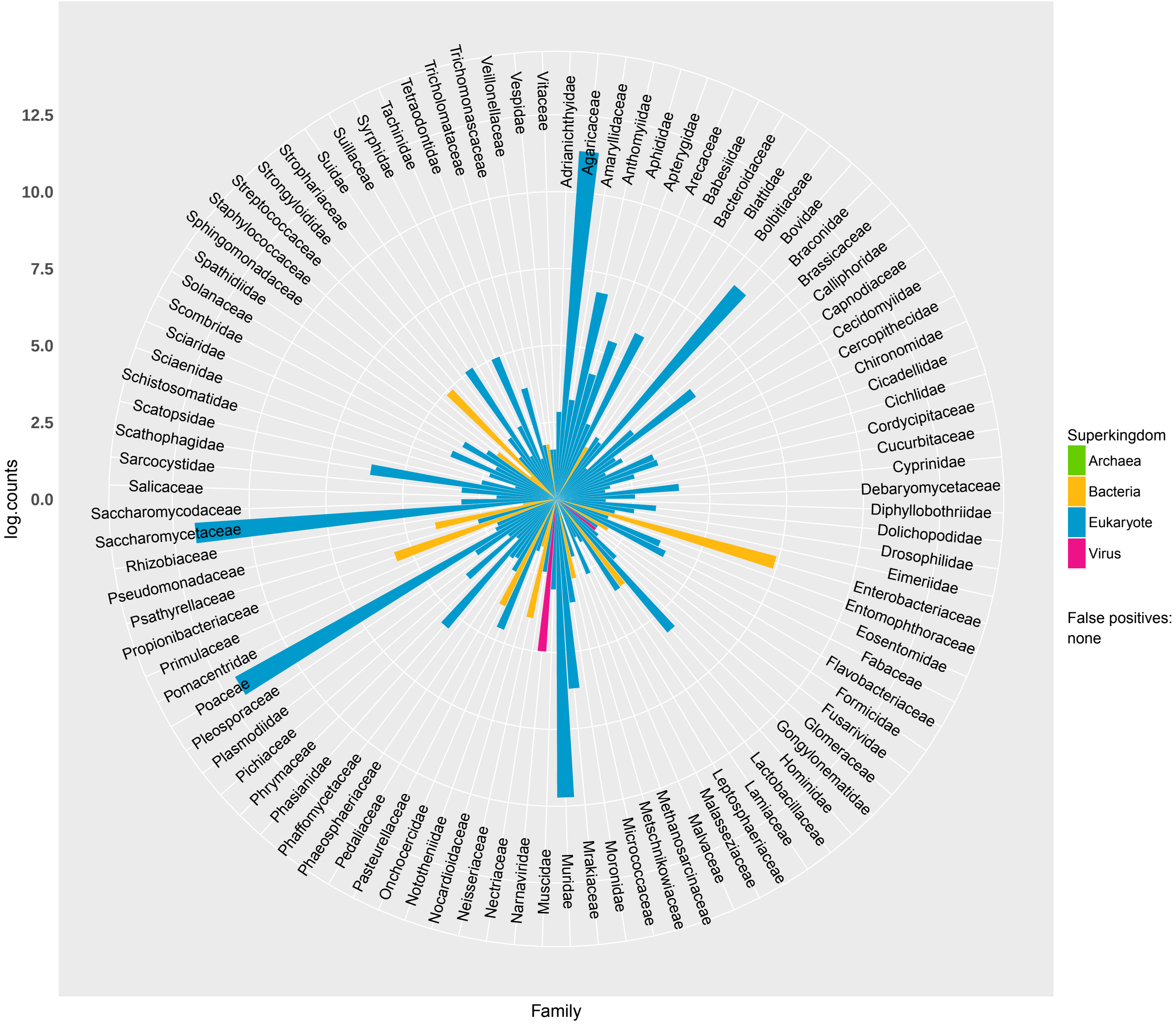

Figure 3: Graphical representation of read counts for the 100 most abundant families. In order to represent all detected superkingdoms, the displayed families are selected from the complete result applying the HighestAverages algorithm from the R package SciencesPo. Families are sorted alphabetically, color-coded according to superkingdoms. Note that the graph is in log-scale.

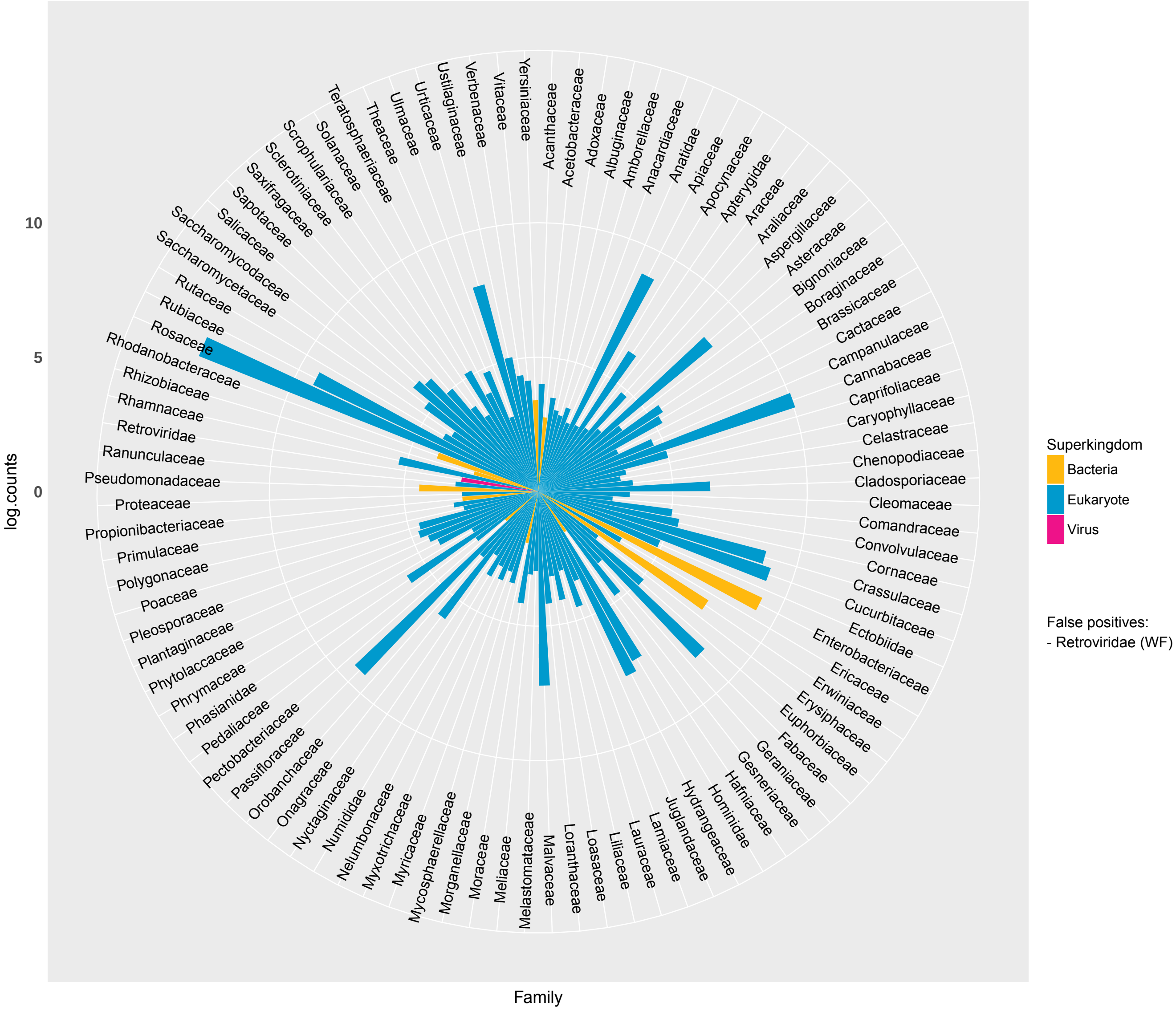

Figure 3: Graphical representation of read counts for the 100 most abundant families. In order to represent all detected superkingdoms, the displayed families are selected from the complete result applying the HighestAverages algorithm from the R package SciencesPo. Families are sorted alphabetically, color-coded according to superkingdoms. Note that the graph is in log-scale.

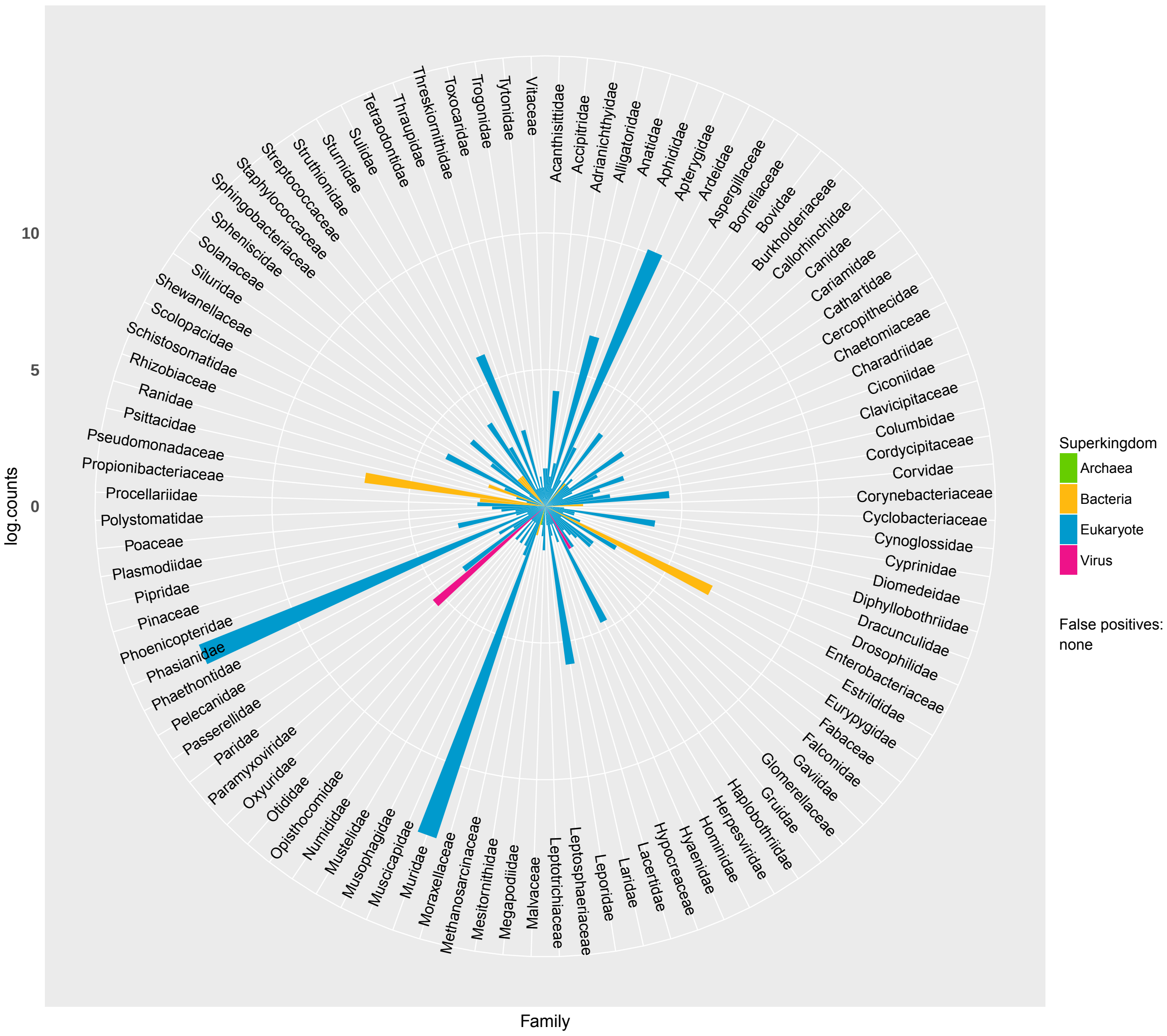

Figure 3: Graphical representation of read counts for the 100 most abundant families. In order to represent all detected superkingdoms, the displayed families are selected from the complete result applying the HighestAverages algorithm from the R package SciencesPo. Families are sorted alphabetically, color-coded according to superkingdoms. Note that the graph is in log-scale.

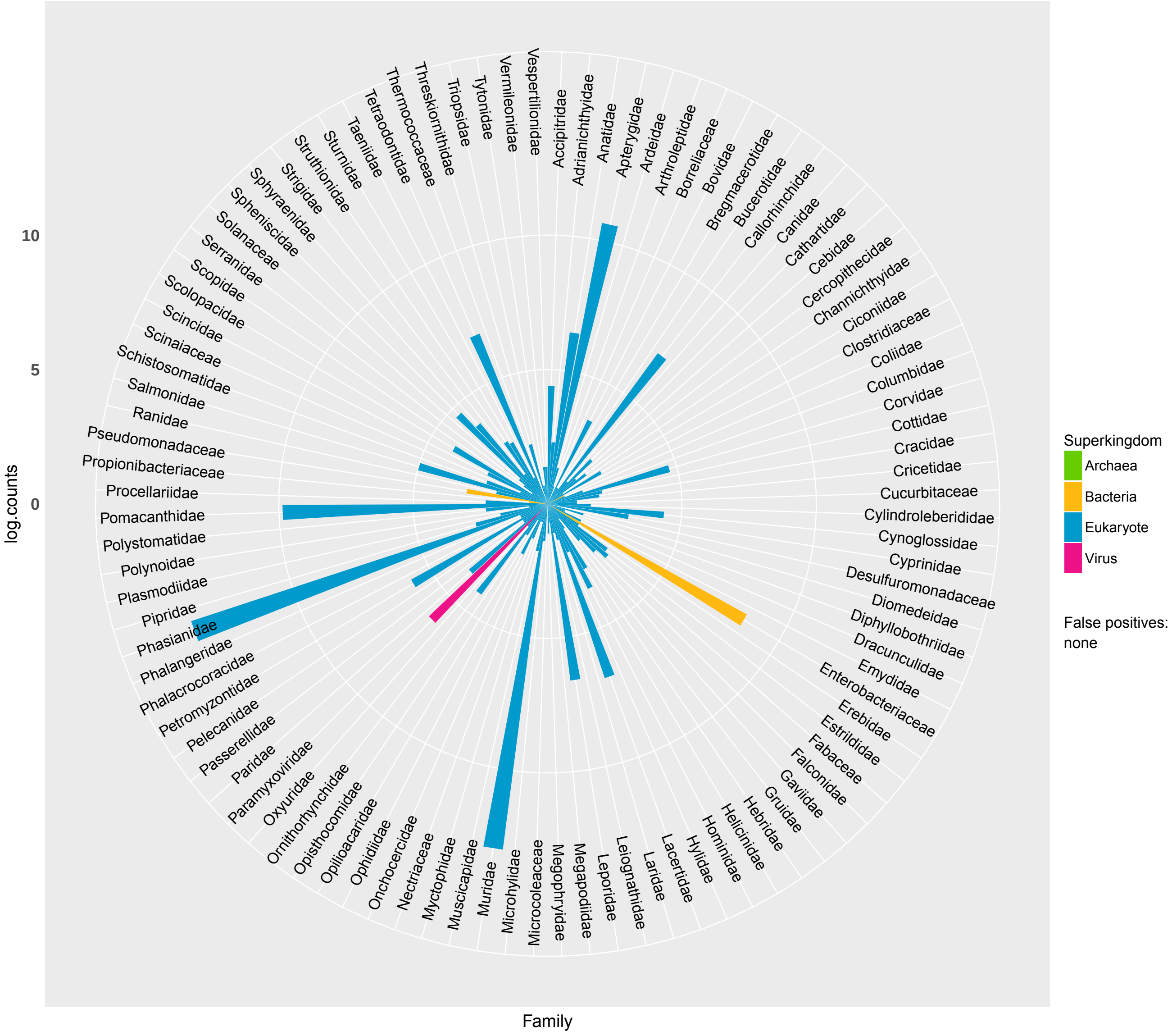

Figure 3: Graphical representation of read counts for the 100 most abundant families. In order to represent all detected superkingdoms, the displayed families are selected from the complete result applying the HighestAverages algorithm from the R package SciencesPo. Families are sorted alphabetically, color-coded according to superkingdoms. Note that the graph is in log-scale.

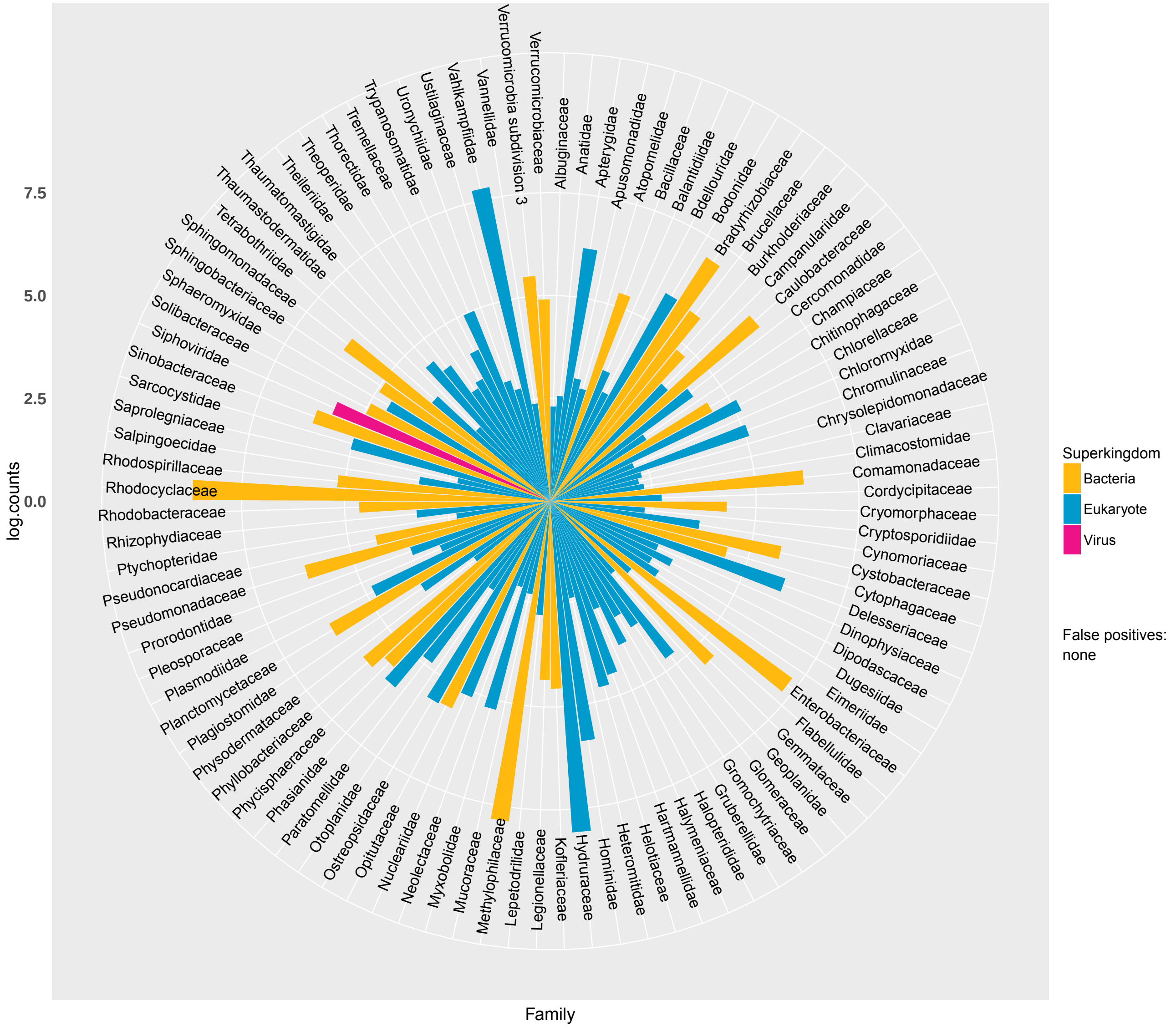

Figure 3: Graphical representation of read counts for the 100 most abundant families. In order to represent all detected superkingdoms, the displayed families are selected from the complete result applying the HighestAverages algorithm from the R package SciencesPo. Families are sorted alphabetically, color-coded according to superkingdoms. Note that the graph is in log-scale.

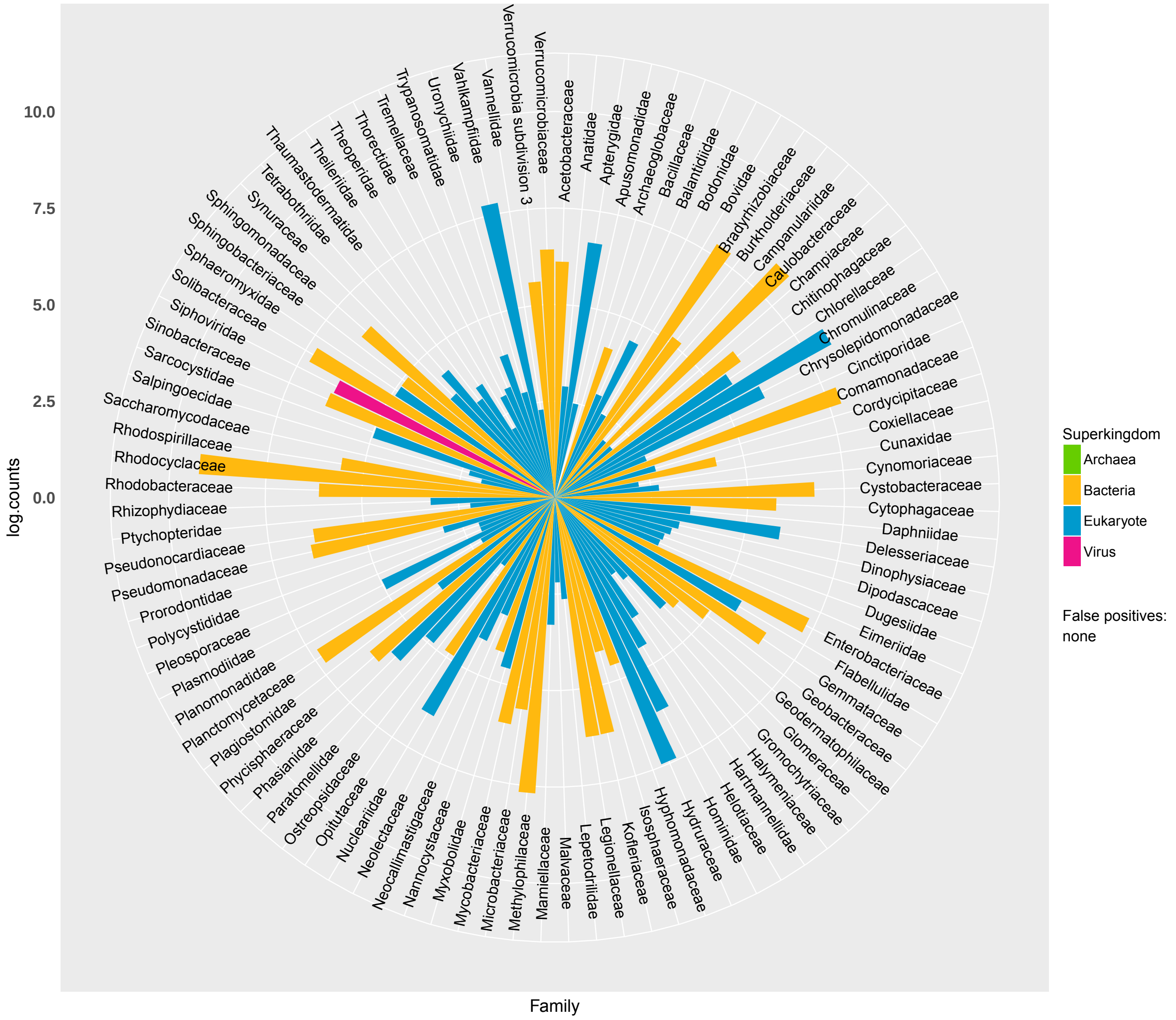

Figure 3: Graphical representation of read counts for the 100 most abundant families. In order to represent all detected superkingdoms, the displayed families are selected from the complete result applying the HighestAverages algorithm from the R package SciencesPo. Families are sorted alphabetically, color-coded according to superkingdoms. Note that the graph is in log-scale.

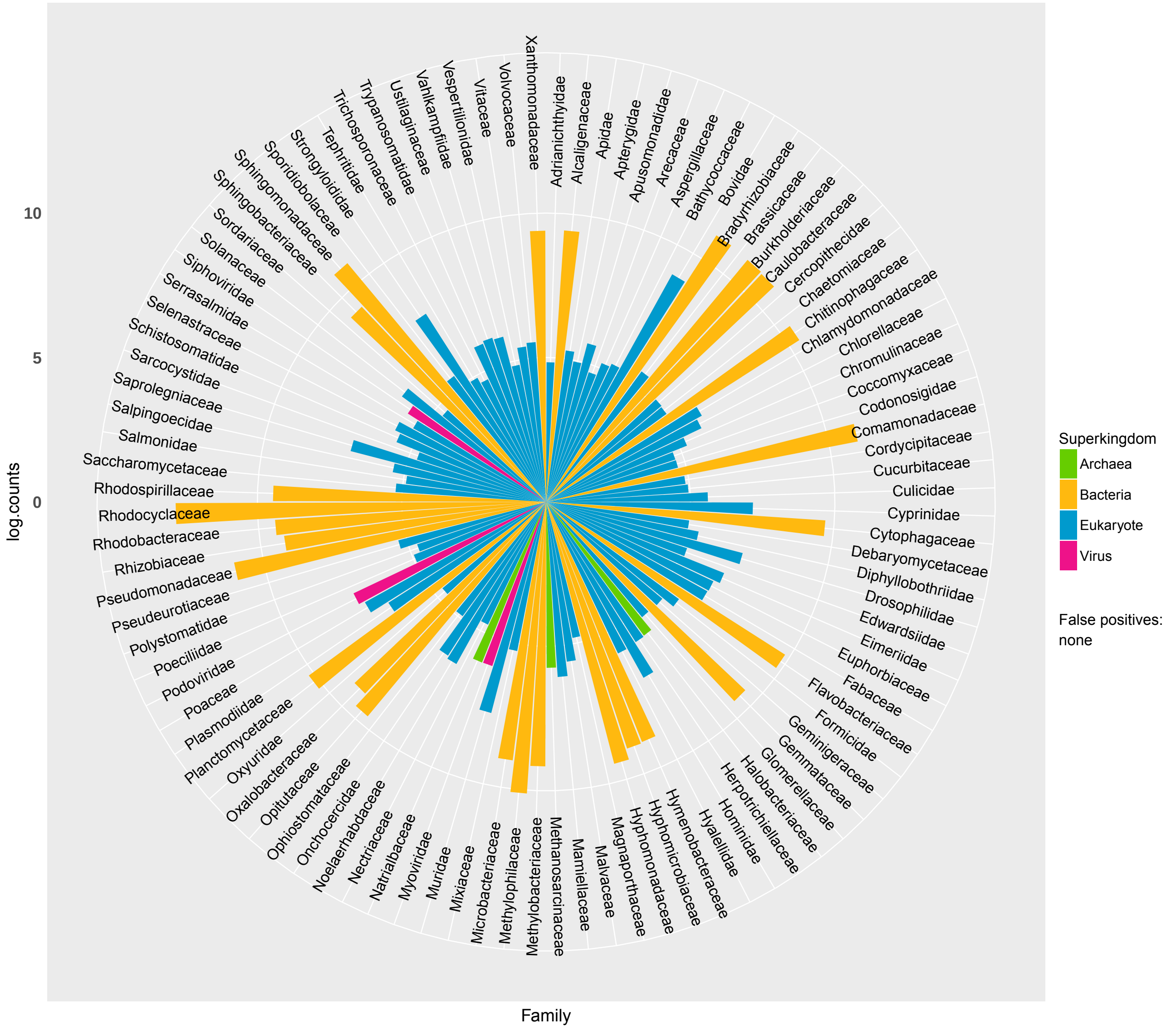

Figure 3: Graphical representation of read counts for the 100 most abundant families. In order to represent all detected superkingdoms, the displayed families are selected from the complete result applying the HighestAverages algorithm from the R package SciencesPo. Families are sorted alphabetically, color-coded according to superkingdoms. Note that the graph is in log-scale.

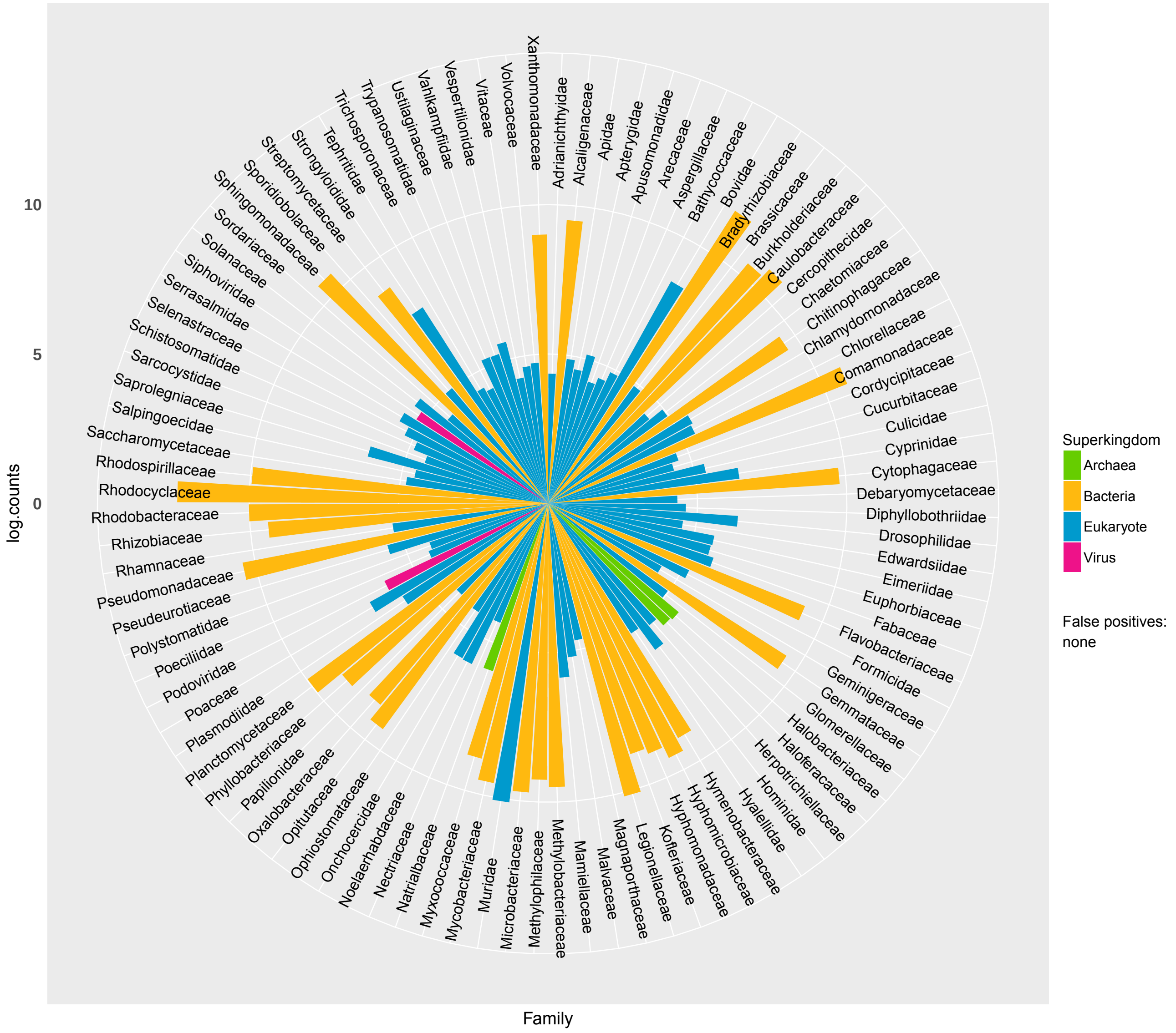

Figure 3: Graphical representation of read counts for the 100 most abundant families. In order to represent all detected superkingdoms, the displayed families are selected from the complete result applying the HighestAverages algorithm from the R package SciencesPo. Families are sorted alphabetically, color-coded according to superkingdoms. Note that the graph is in log-scale.

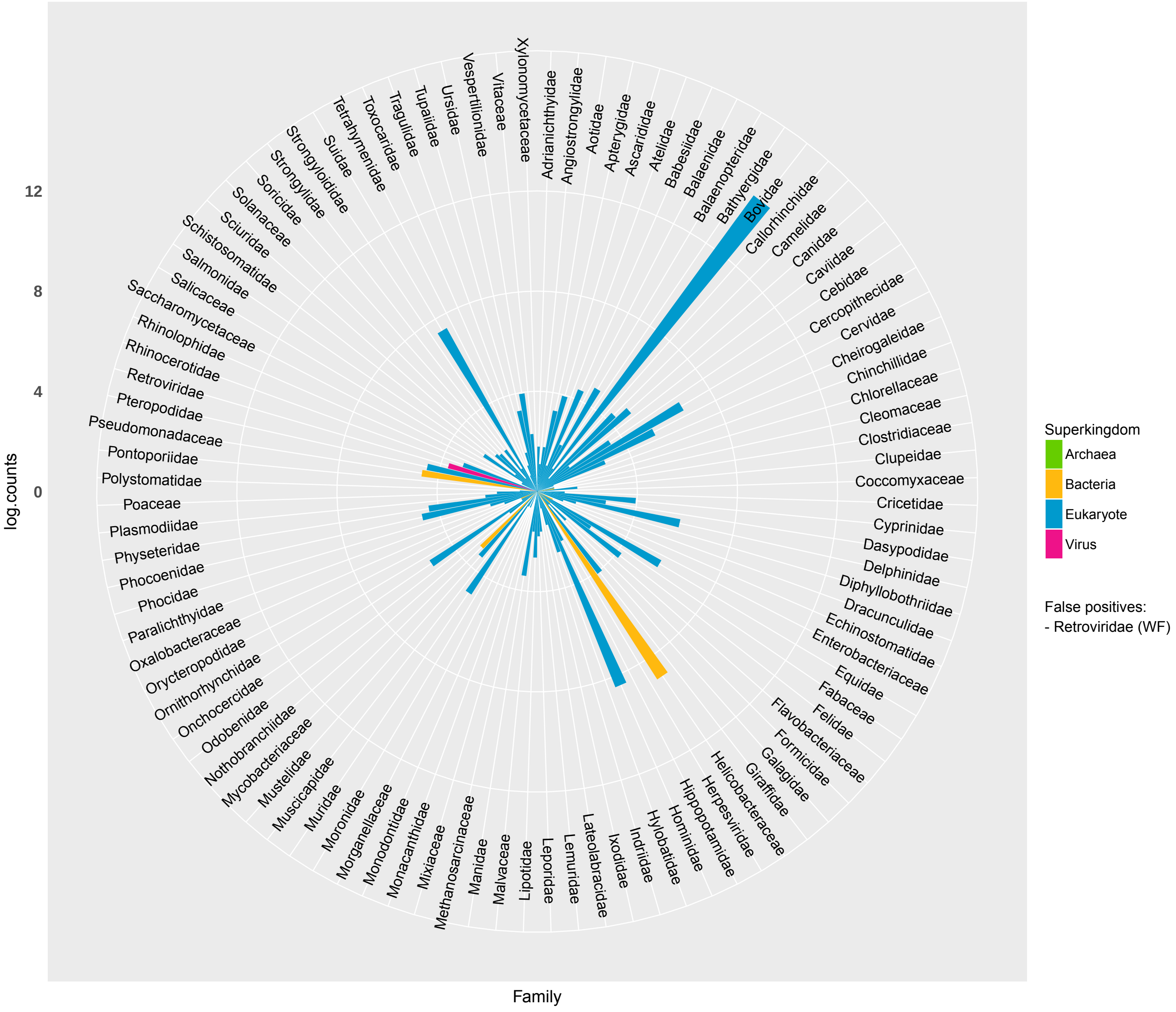

Figure 3: Graphical representation of read counts for the 100 most abundant families. In order to represent all detected superkingdoms, the displayed families are selected from the complete result applying the HighestAverages algorithm from the R package SciencesPo. Families are sorted alphabetically, color-coded according to superkingdoms. Note that the graph is in log-scale.

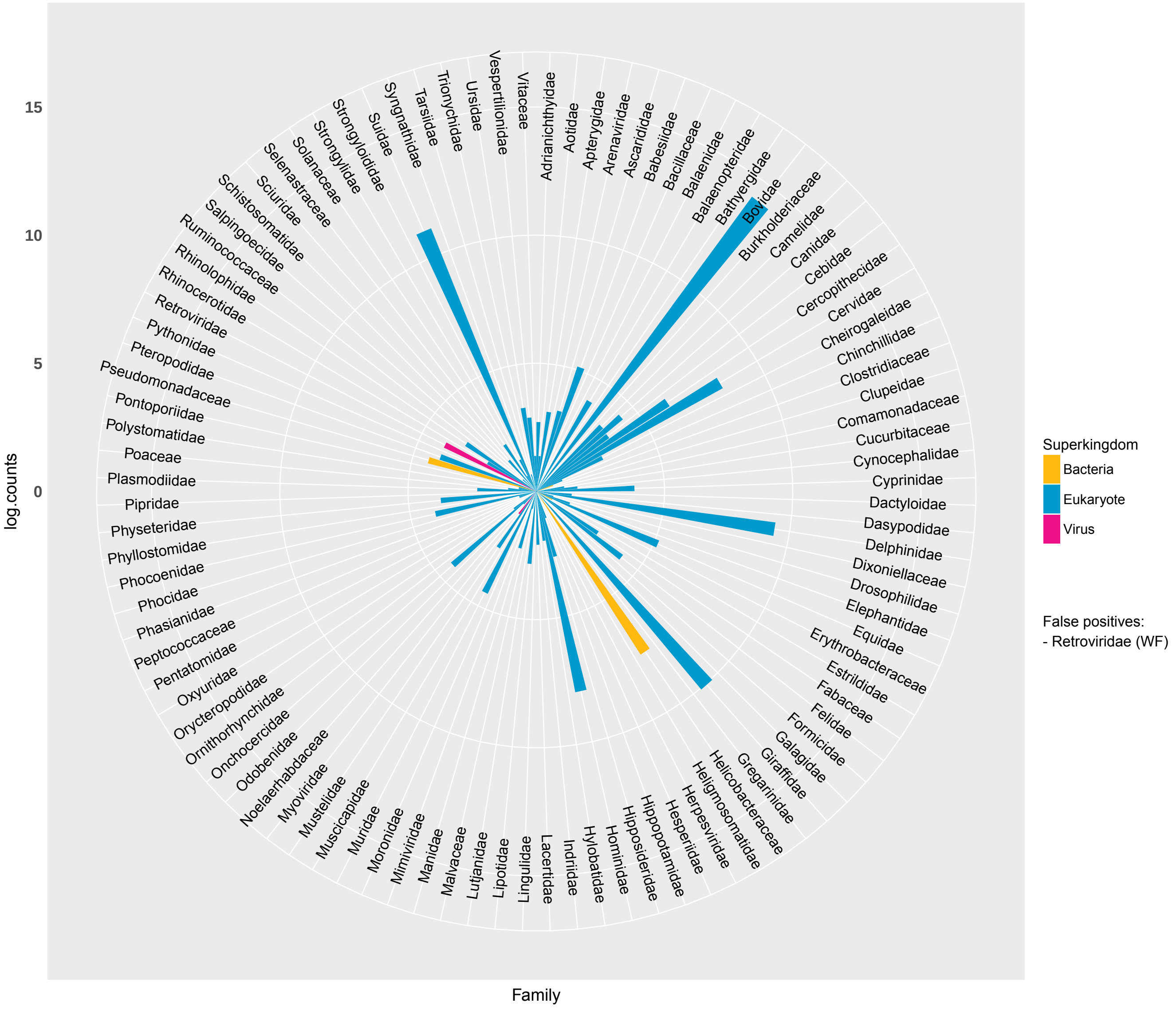

Figure 3: Graphical representation of read counts for the 100 most abundant families. In order to represent all detected superkingdoms, the displayed families are selected from the complete result applying the HighestAverages algorithm from the R package SciencesPo. Families are sorted alphabetically, color-coded according to superkingdoms. Note that the graph is in log-scale.

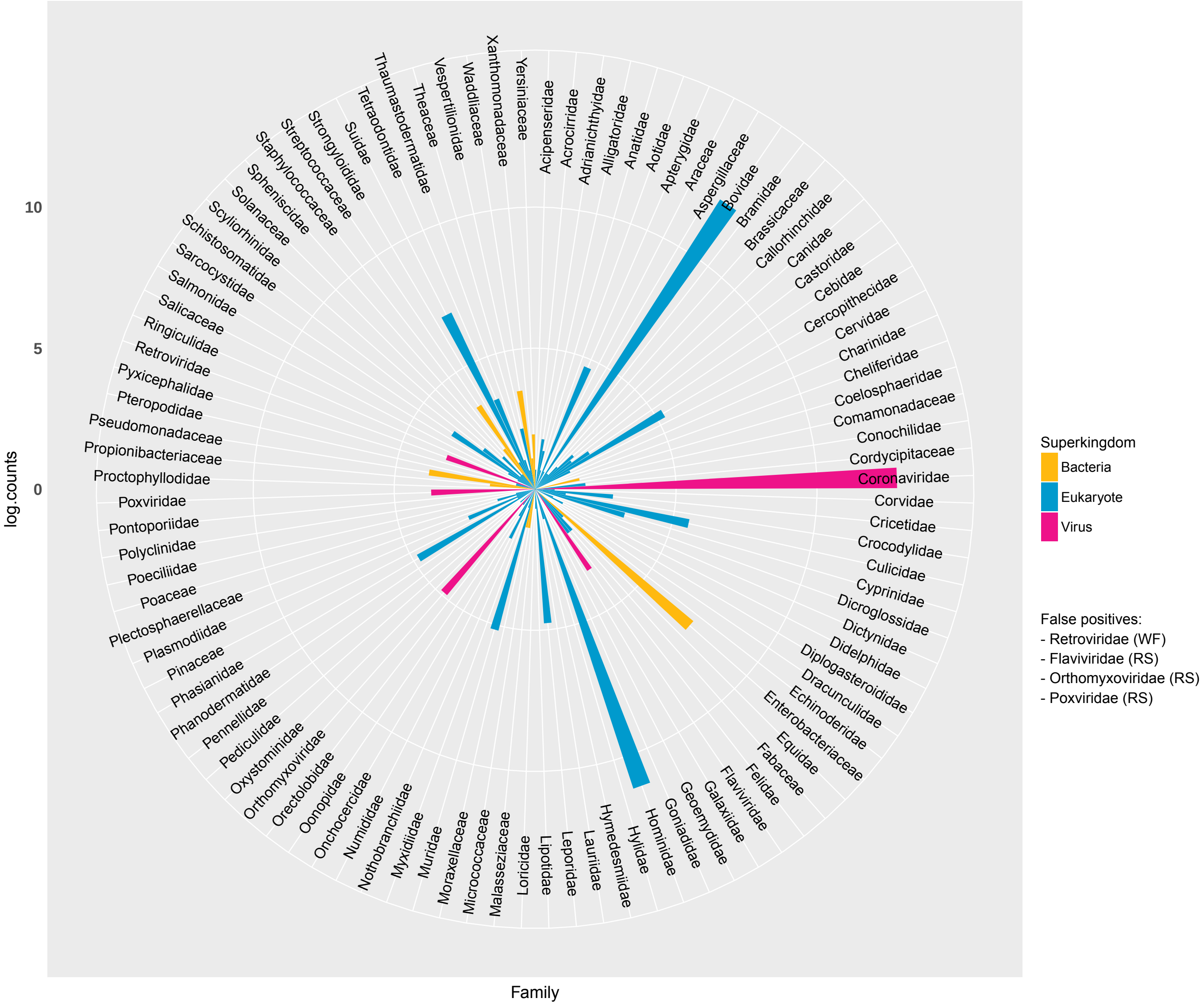

Figure 3: Graphical representation of read counts for the 100 most abundant families. In order to represent all detected superkingdoms, the displayed families are selected from the complete result applying the HighestAverages algorithm from the R package SciencesPo. Families are sorted alphabetically, color-coded according to superkingdoms. Note that the graph is in log-scale.

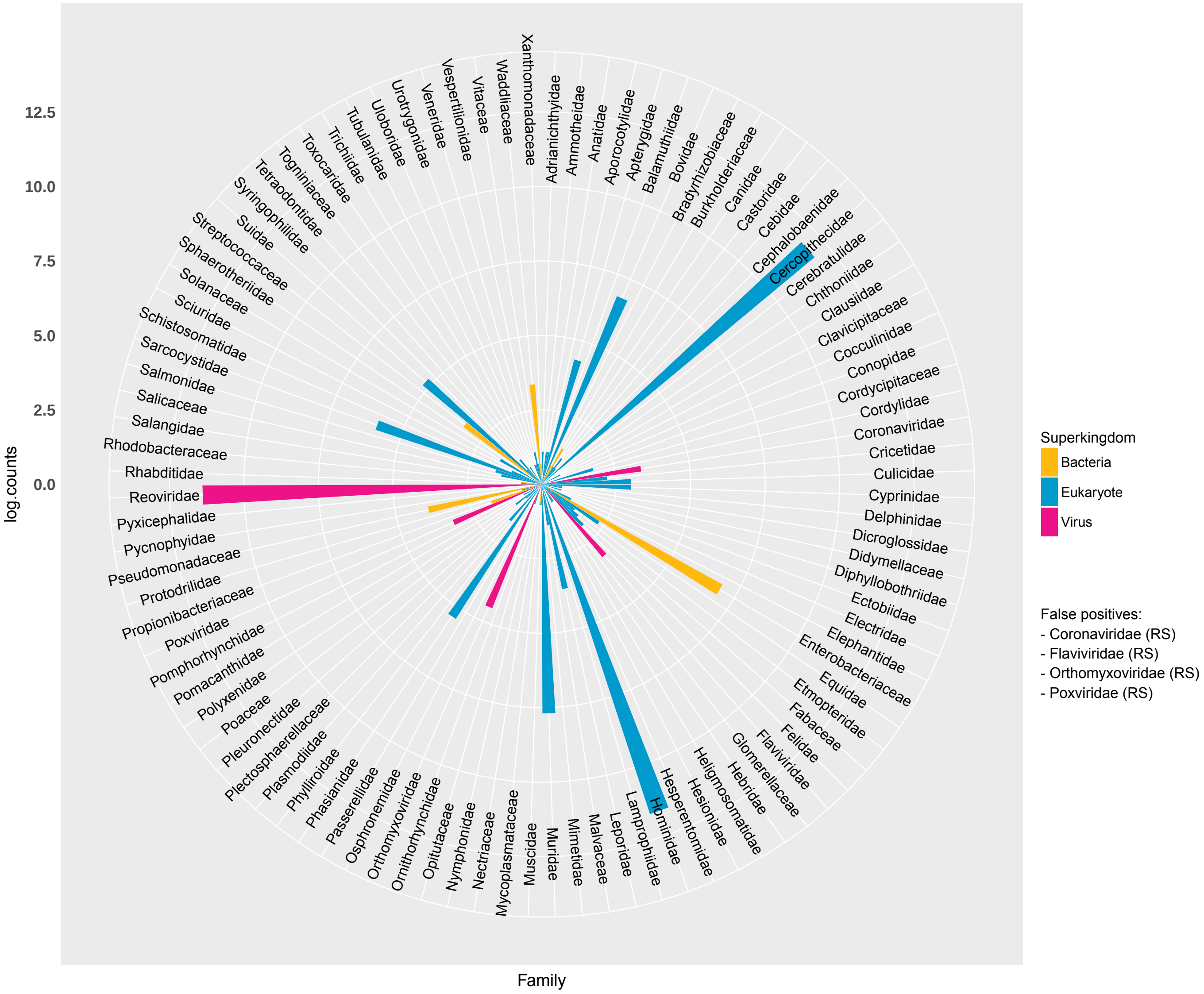

Figure 3: Graphical representation of read counts for the 100 most abundant families. In order to represent all detected superkingdoms, the displayed families are selected from the complete result applying the HighestAverages algorithm from the R package SciencesPo. Families are sorted alphabetically, color-coded according to superkingdoms. Note that the graph is in log-scale.

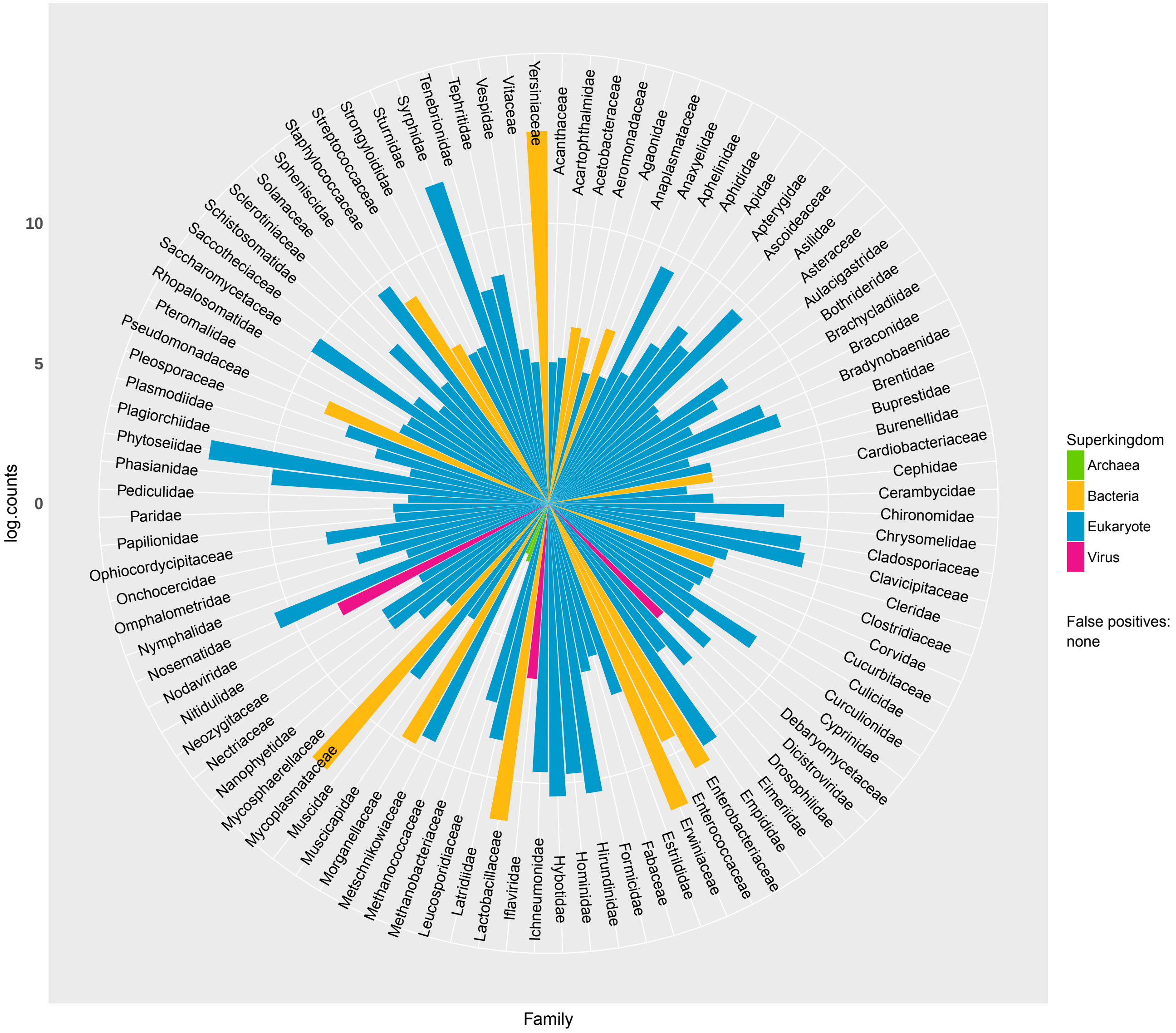

Figure 3: Graphical representation of read counts for the 100 most abundant families. In order to represent all detected superkingdoms, the displayed families are selected from the complete result applying the HighestAverages algorithm from the R package SciencesPo. Families are sorted alphabetically, color-coded according to superkingdoms. Note that the graph is in log-scale.

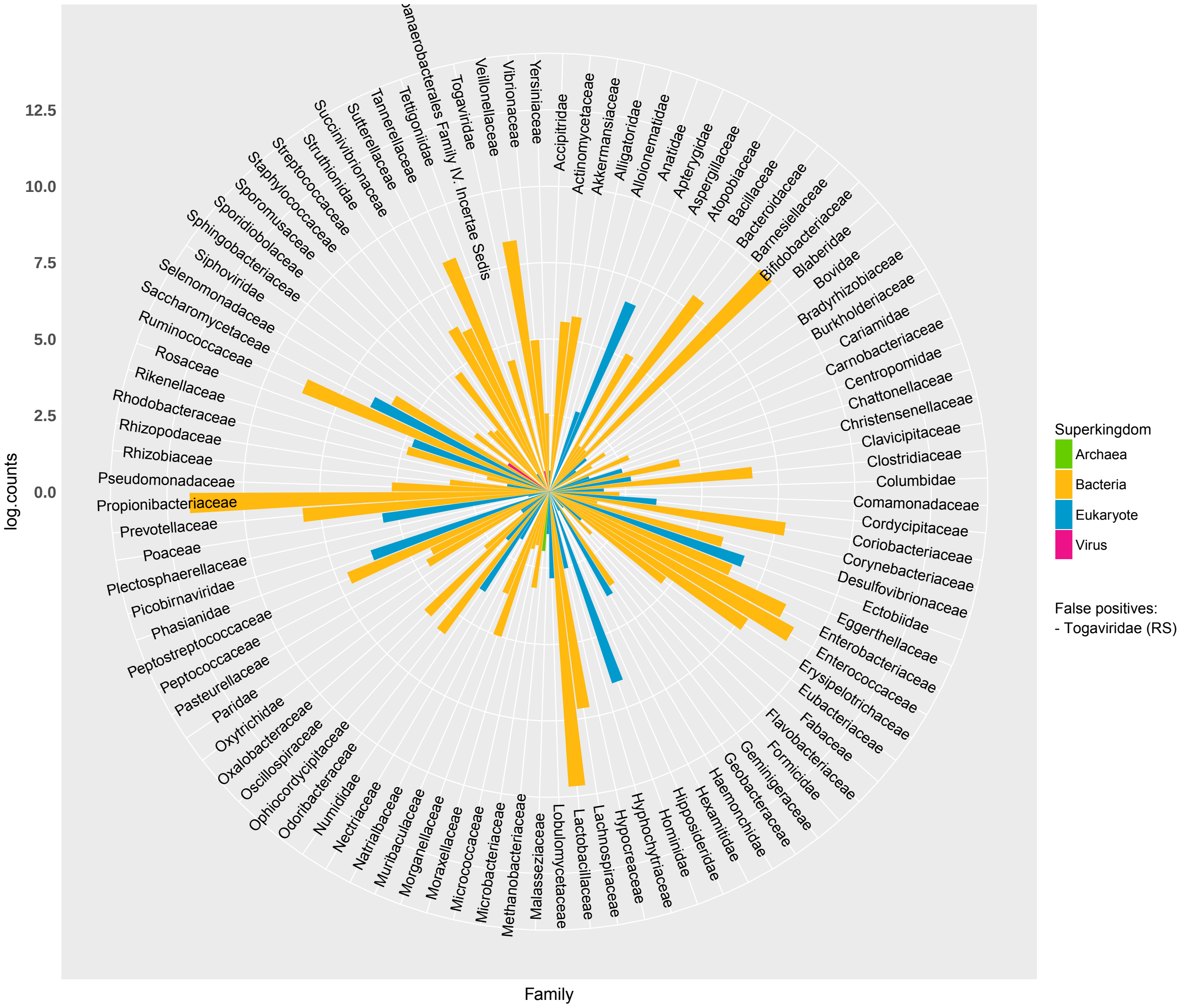

Figure 3: Graphical representation of read counts for the 100 most abundant families. In order to represent all detected superkingdoms, the displayed families are selected from the complete result applying the HighestAverages algorithm from the R package SciencesPo. Families are sorted alphabetically, color-coded according to superkingdoms. Note that the graph is in log-scale.
